# Getting Deeper into the Molecular Events of Heme Binding Mechanisms: A Comparative Multi-level Computational Study of HasAsm and HasAyp Hemophores

**DOI:** 10.1021/acs.inorgchem.2c02193

**Published:** 2022-10-17

**Authors:** Laura Tiessler-Sala, Giuseppe Sciortino, Lur Alonso-Cotchico, Laura Masgrau, Agustí Lledós, Jean-Didier Maréchal

**Affiliations:** † Insilichem, Departament de Química, Universitat Autònoma de Barcelona, 08193 Bellaterra, Barcelona, Spain; ‡ Institute of Chemical Research of Catalonia (ICIQ), The Barcelona Institute of Science and Technology, 43007 Tarragona, Spain; § Zymvol Biomodeling, Carrer Roc Boronat 117, 08018 Barcelona, Spain

## Abstract

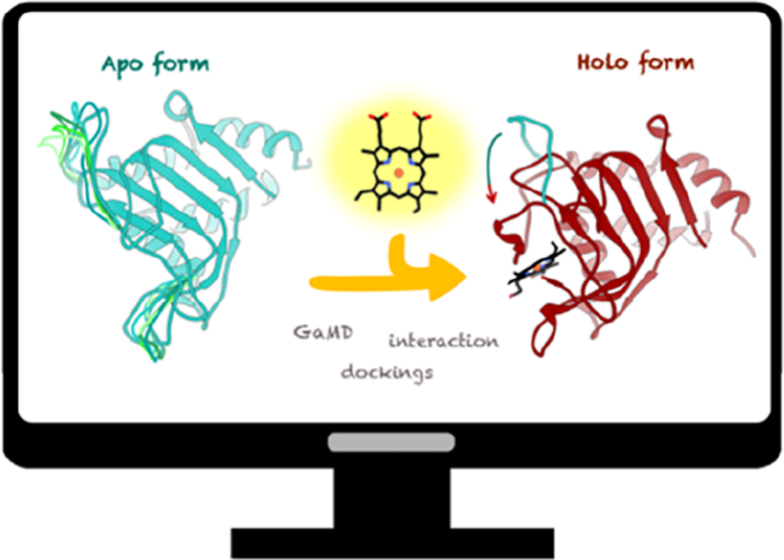

Many biological systems obtain their activity by the inclusion of metalloporphyrins into one or several binding pockets. However, decoding the molecular mechanism under which these compounds bind to their receptors is something that has not been widely explored and is a field with open questions. In the present work, we apply computational techniques to unravel and compare the mechanisms of two heme-binding systems, concretely the HasA hemophores from Gram negative bacteria *Serratiamarcescens* (HasAsm) and *Yersinia pestis* (HasAyp). Despite the high sequence identity between both systems, the comparison between the X-ray structures of their apo and holo forms suggests different heme-binding mechanisms. HasAyp has extremely similar structures for heme-free and heme-bound forms, while HasAsm presents a very large displacement of a loop that ultimately leads to an additional coordination to the metal with respect to HasAyp. We combined Gaussian accelerated molecular dynamics simulations (GaMDs) in explicit solvent and protein–ligand docking optimized for metalloligands. GaMDs were first carried out on heme-free forms of both hemophores. Then, protein–ligand dockings of the heme were performed on cluster representatives of these simulations and the best poses were then subjected to a new series of GaMDs. A series of analyses reveal the following: (1) HasAyp has a conformational landscape extremely similar between heme-bound and unbound states with no to limited impact on the binding of the cofactor, (2) HasAsm presents as a slightly broader conformational landscape in its apo state but can only visit conformations similar to the X-ray of the holo form when the heme has been bound. Such behavior results from a complex cascade of changes in interactions that spread from the heme-binding pocket to the flexible loop previously mentioned. This study sheds light on the diversity of molecular mechanisms of heme-binding and discusses the weight between the pre-organization of the receptor as well as the induced motions resulting in association.

## Abstract 2

Heme-containing enzymes and proteins are important for many biological and biotechnological processes. However, very little is known about heme-binding mechanisms. To shed light on this, we report a multi-level approach combining Gaussian accelerated molecular dynamics and protein−ligand dockings optimized for metallic moieties. The protocol unveils the difference in heme recruitment between HasAsm and HasAyp hemophores and shows its possible applicability to other heme-binding proteins.

## Introduction

The inclusion of inorganic moieties in proteins leads to unique structural and catalytic features that overcome the limitations of a purely organic living world. More than 40% of all proteins in the cells exploit one or several metals to perform their functions.^1^ Decoding the interactions between inorganic moieties and biological partners is therefore a fundamental question in understanding the origin of life as well as in opening new biotechnological routes, like in the design of artificial metalloenzymes obtained from the insertion of inorganic homogeneous catalysts into proteic hosts.^2−4^ However, metal-mediated binding processes are one of the most complex questions molecular biology could address. The biological–inorganic interplay overcomes standard knowledge of both chemical and biological sciences, and assessing the relative contributions of both partners in the recognition process is extremely challenging.^5^

**Iron protoporphyrin IX** or **heme *b*** is one of the most ubiquitous metal-containing ligands in nature. It is well known to contribute to crucial biological functions, like transport/storage of O_2_, electron transfer, or redox catalysis.^6^ More recently, heme has also been found to participate as a signaling molecule in cellular processes like transcription regulation, protein complex assembly, microRNA processing, or cell growth and differentiation.^7^ Therefore, heme is a prototypical system to study the binding of metallic cofactors to proteins, although there is no clear molecular description of heme uptake.

One could expect several scenarios in heme-binding mechanisms that include conformational selection and induced fit. Conformational selection sustains that several unbound states of the protein exist in equilibrium and the ligand binds preferably to one or several well pre-organized ones.^8^ Induced fit implies that the conformational change of the receptor is a product of the entrance of the ligand.^9^ Both phenomena are likely to participate in ligand binding with different weights depending on the system, and in some cases, one may prevail over the other.^10,11^ In spite of extensive research of substrate binding to hemoproteins as P450 or cytochromes,^12^ the molecular mechanism under which the heme binds to its receptor has as yet been rarely explored.^13^

Spectroscopic and crystallographic data on heme proteins have shown that the tertiary structure of the apo and holo forms are in general similar.^13^ Two main mechanisms seem to irrupt, though. In some systems like myoglobine,^14^ cytochrome *b*5,^15,16^ or cytochrome *b*562,^17,18^ there are some secondary structure rearrangements that occur because of the binding and generally in the proximal side of the heme. In others, only subtle rearrangements of the secondary structure are observed.^19^ The latter systems are generally referred to as transient heme-binding proteins because they allow fast association–dissociation mechanisms. Among those systems are heme-chaperones, like HemS systems,^19^ or hemophores from the HasA family. The crystal structures of HasA members present, however, some variability that could give further insights into more complex heme-binding mechanisms.

**Figure 1:**
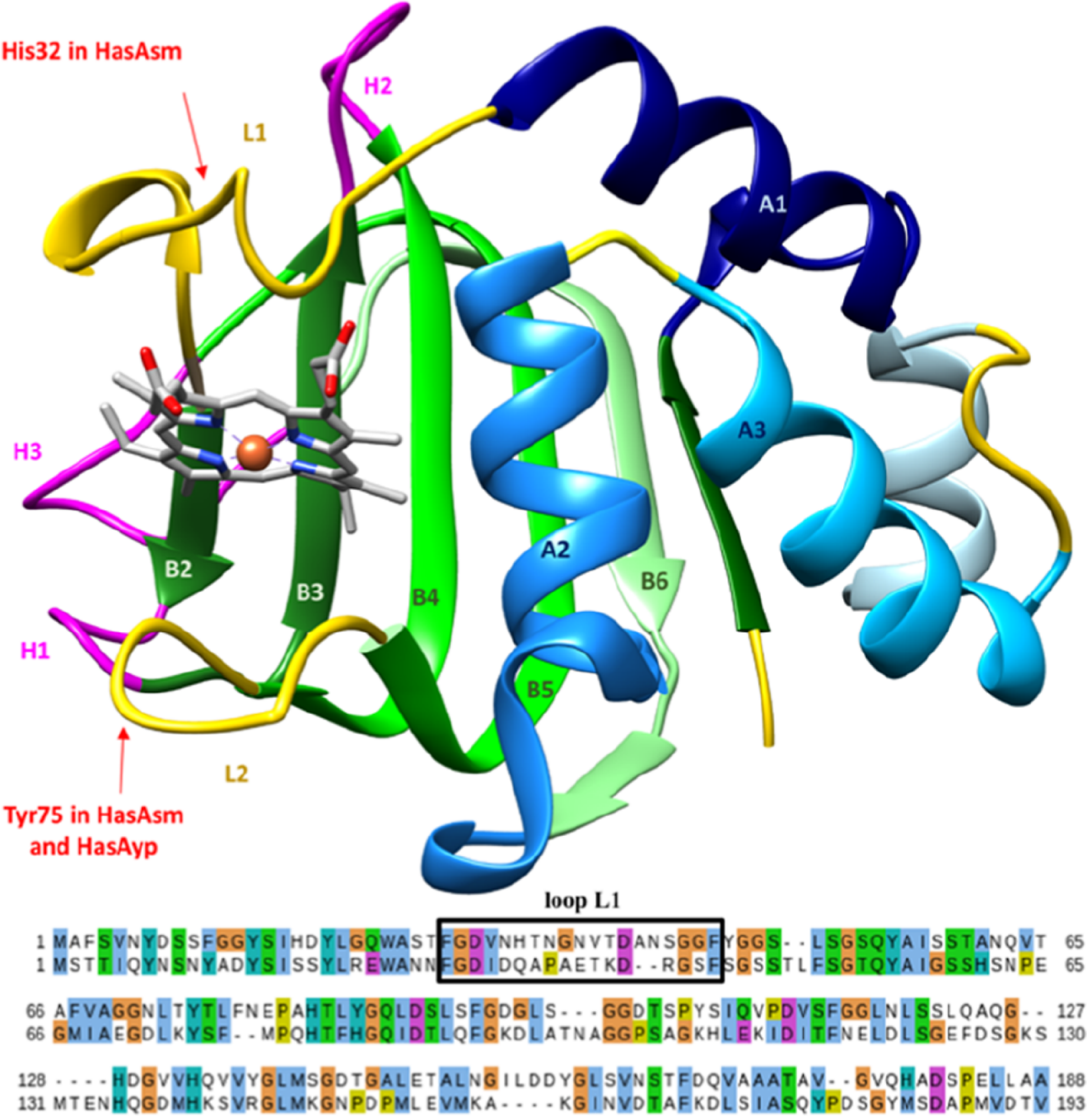
Overall structure of hemophore HasA with names of secondary structure regions and heme coordinators indicated. Sequence alignment between HasAsm (up) and HasAyp (down) in ClustalX colors.

HasAs are extracellular heme-binding proteins that Gram-negative bacteria use for their heme uptake. They are able to acquire **free** or **hemoprotein-bound heme** and to deliver it to a specific receptor at the cell surface (HasR), whereby the heme is internalized and used as an iron source.^20^ Interestingly, two hemophores from this family seem to have very distinctive heme-binding mechanisms: HasA from *Yersenia pestis* (HasAyp) and HasA from *Serratia marcescens* (HasAsm). HasA structure contains a α + β fold structure in which the heme is found between loops L1 (27–43) and L2 (74–84) at the interface of the α and β domains.^20,21^ Both have very similar structures and a sequence identity of 31% (Figure 1), with the less-conserved part being located at loop L1.^22^

**Figure 2:**
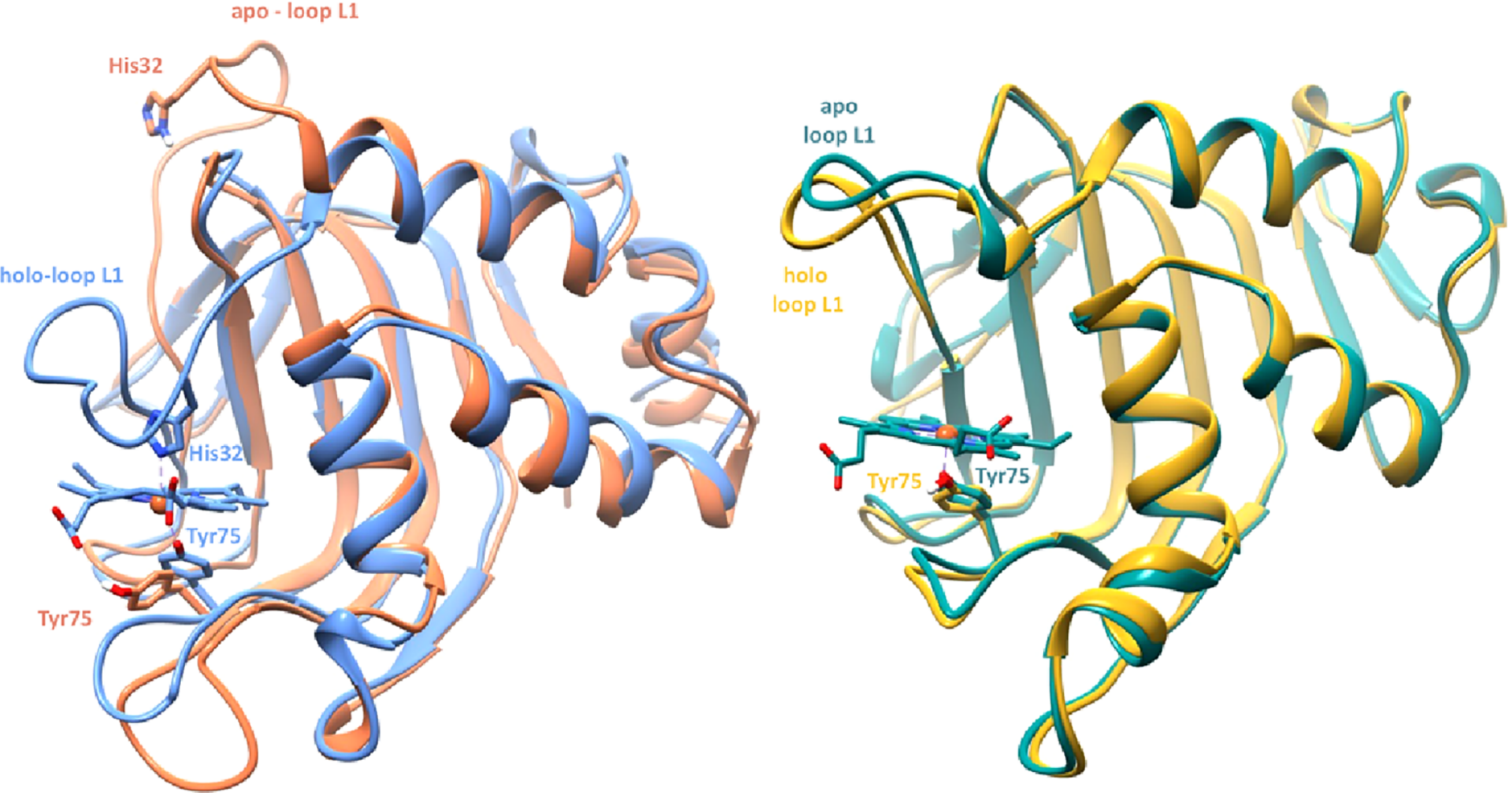
Structural overlap between apo (1YBJ) and holo (1DKH) forms of hemophore HasA from *S. marcescens* (left) and apo (4JES) and holo (4JET) forms from *Y. pestis* (right). Shifts between apo and holo forms in loop L1 are indicated.

Interestingly, experimental structures of the apo and holo forms of HasAyp and HasAsm show striking differences (Figure 2): (1) L1 position is almost invariant in HasAyp in apo and holo forms with a closed conformation upon the heme-binding pocket, while a very large conformational change is observed in HasAsm, with an opened form in the apo structure and a closed form in the holo one with a motion of about 15 Å, and (2) this is also related to a difference in axial coordination of the iron in the two holo forms; the iron is bound to Tyr75 for the L2 loop in HasAyp, whereas it binds to both Tyr75 (L2) and His32 (L1) in HasAsm. This shows that very distinct binding mechanisms could occur although spectroscopic data and the comparison between the apo and holo structures are not able to provide a clear molecular understanding. In this regard, computational tools could be a very valuable asset.

Most of the computational studies regarding hemophores have been performed on the hemophore HasA from *Pseudomonas aeruginosa*, a system that displays geometrical features similar to those of HasAsm. In their first work, Rivera et al. used targeted molecular dynamics simulations (TMD) and identified a series of interactions and motions in helices α2 and α3 upon heme binding that could be involved in the closing of loop L1.^23^ In a later study, the transition from the apo to the holo conformation of HasAp was not observed in a 100 ns classical MD. However, MD of the Arg33Ala mutant pointed that this residue could be important for controlling the closing of loop L1. Strikingly, Arg33 is not present in HasAsm.^24^ To date, the unique computational study on HasAsm also involves TMD simulations for the heme transfer to HasR, and no simulations have been reported on HasAyp.^25^ It is important to notice that TMD,^26^ highly informative for molecular mechanisms, implies that simulations are forced toward a defined final structure by using steering forces. Such restraints do not allow us to assess the conformational space that the apo-protein explores in standard conditions and how this is related to the heme-binding mechanism.

This study herein pretends to give further insights into the heme-binding process in hemophores considering that it represents a ground material for similar systems. The underlying methodology consists in providing a wide conformational exploration of the apo-hemophore structures, intermediate heme-bound complexes, and the final complex to evaluate the pre-organization degree and identifies possible induced effects. To do so, we bridge together all-atom MD with protein–ligand docking. In particular, we apply Gaussian accelerated MD (GaMD), which allows simulations of long-range motions and explores the conformational space of the system without imposing specific geometric restraints. Moreover, in both approaches, we use updated parameters for dealing with the metallic moieties, overcoming the challenges still present for metal ion description in force field-based approaches. The results give interesting insights into the heme-binding mechanisms in heme binding proteins that could better envision protein engineering processes for heme-containing enzymes and for artificial enzymes.

## Methodology

### Overall Pipeline

**Scheme 1:**
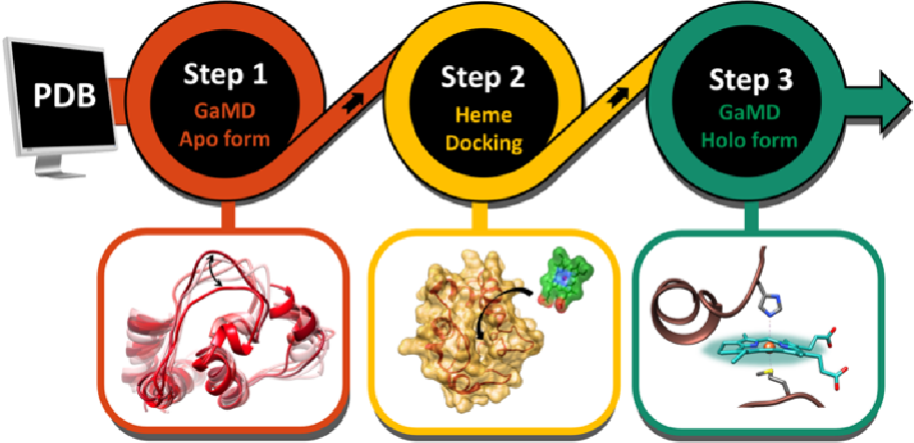
Multi-level Computational Protocol Followed

The computational framework for this work combines molecular dockings and GaMD simulations. It starts with a crystallographic heme-free form available at the Protein Data Bank (PDB) to run GaMD simulations. The analysis of the trajectories, including cluster analysis, is followed by dockings of heme. From the docking results, GaMD simulations on heme-bound intermediates are performed to study the effect of heme binding to the protein conformation (Scheme 1).

### System Setup

The X-ray structures of the apo forms of heme-containing proteins were obtained from the PDB.^27^ The PDB code 1YBJ was used for the apo form of HasAsm and 4JES for the apo form of HasAyp. Calculations on the experimental holo HasAsm were performed on the structure with the PDB code 1DKH. All structures were cleaned by removing crystallographic waters and small molecules present in the PDB file using UCSF Chimera.^28^ Hydrogen atoms were added using Chimera, and webserver H++^29^ was also used to double-check the protonation state of ionizable groups.

### Docking Calculations

Dockings were performed with GOLD5.2,^30^ using a simulation box of 10–15 Å and centered at the binding site of each protein. Side-chain flexibility on the binding site residues was considered when required using the default rotamer library. For higher accuracy, the minimum number of operations was set to 100,000, and the number of the Genetic algorithm (GA) runs to 50. An optimized version of GoldScore as a scoring function capable of predicting metal–protein interactions was used.^31^ All the solutions were analyzed using GaudiView.^32^

### GaMD Molecular Dynamics Simulations

Unconstrained enhanced sampling was performed with the Gaussian accelerated molecular dynamics (GaMD) method. This method consists in adding adaptively a harmonic boost potential to smoothen the potential energy surface and to explore massive conformational landscape without predetermined reaction coordinates or any kind of restraints. Furthermore, GaMD simulations provide speedup simulations and allow capturing events over a longer time. This method allows a larger exploration of the conformational space and allows us to identify different states of the biomolecules. A short conventional MD always proceeds with a GaMD calculation as a preparatory stage that collects potential statistics.^33^ This technique has been applied using AMBER18’s code using the coordinates extracted from a classical MD of 10–20 ns as a starting point.^34^ The parameter for the GaMD igamd was set to 3, which applies a force to dihedrals and to the total potential energy, and the threshold energy was set to the lower bound, IE = 1.

GaMD simulations were prepared with the xleap^35^ using the force field ff14SB for proteins, GAFF for non-standard residues, ions94.lid for ions, and TIP3P for water. In the case of metalloproteins, metal parameters were obtained using the MCPB.py approach.^36^ In MCPB.py, the charges were calculated using RESP, and the force constants and equilibrium parameters between the metal and the residues were calculated using the Seminario method.^37^ Optimization and frequency calculations of heme at the DFT level were performed with Gaussian09^38^ in water solvent (SMD continuum model).^39^ The B3LYP hybrid functional including Grimme’s dispersion D3^40^ was used with SDD + F (Fe) + 6-31G(d,p) as the main group.^41^ Different Fe-oxidation states (Fe^2+^ and Fe^3+^) and multiplicity (low and high) were taken into account. For the sake of the presentation of the work, the results presented in the main text correspond to calculations performed with the Fe-oxidation state, which was +3 high spin when pentacoordinated and low spin when hexacoordinated; those are apparently the most accepted oxidation and spin states from the literature. Still, Fe(II) simulations were also performed with the same spin state considerations (see ESI).

All GaMD simulations were set up solvating the protein using an explicit solvent approach, in which the protein was embedded into a cubic box adding as counter ions Na^+^, specifically 10 and 12 Na^+^ for HasAyp for apo and holo forms, respectively, and 13 and 15 Na^+^ for HasAsm for apo and holo forms, respectively. GaMD simulations were carried out under periodic boundary conditions using AMBER18.^34^ For the preparatory MD simulations, energy minimization was performed to avoid steric clashes and relax the system, followed by several equilibration steps in which the system was heated from 100 to 300 K. Finally, a production run of 100 ns was carried out. GaMD simulations started from coordinates of MD after 10–20 ns, and an equilibration of 50 ns was performed followed by a production run of 800 ns. In all cases, three replicas of each calculation were performed in order to assure convergence and a full exploration of the conformational space.

### Analysis of GaMD Simulations

All GaMD trajectories were processed using cpptraj implemented in Ambertools18,^34^ and cluster analysis was performed with MDtraj.^42^ The trajectories were considered converged by assessing the variation in root-mean-square deviation (rmsd) with respect to the initial structure, all-to-all rmsd, RSMF, PCA, and a clustering counting method.^43^ All calculations were performed using carbon alpha.

For interaction analysis, the getContacts.py^44^ script was used and tuned in order to analyze hydrogen bonds, salt bridges, π–stacking, and hydrophobic interactions along the GaMD simulations. All contact results from different replicas were combined to obtain the mean frequency of contacts through all replicates. To study interaction differences between simulation apo–holo pairs, the difference of interactions was calculated and normalized. The results were simulated into UCSF Chimera using pseudo-bond representation. To calculate free energies, GaMD reweighting was performed with the PyReweighting toolkit and the Maclaurin method.^45^

## Results and Discussion

In this section, first HasAyp and HasAsm hemophore are analyzed and discussed separately, and then, a comparison between the two hemophores is presented.

### HasA from *Y. pestis*

As previously mentioned, the apo and the holo crystallographic forms of hemophore HasAyp have similar structures, and loop L1 remains mostly static (Figure 2b). Still, we wanted to study better the conformational space and assess if changes in L1 could exist with respect to the X-ray structures. For example, would it be possible that the loop could eventually reach conformation like HasAsm in the absence of heme? Three replicas of 800 ns GaMD simulations were performed. The combination of five analysis tools (PCA, clustering, rmsd, all-to-all rmsd, and RMSF) revealed that after 800 ns, all three replica (2.4 μs) simulations had converged, and the conformational space sampled minimal changes in the overall structure (Figure S1).

**Figure 3:**
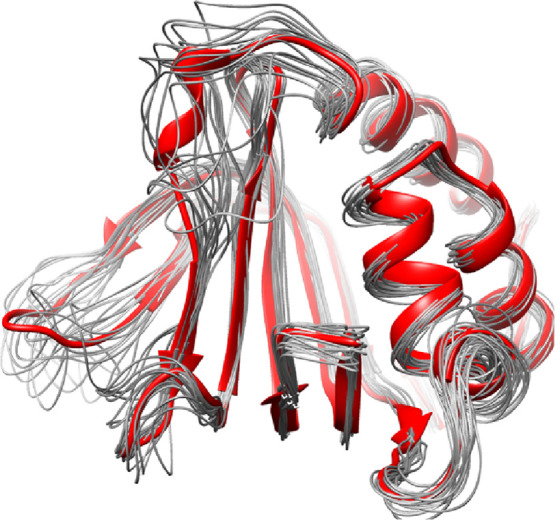
Main clusters of GaMD simulation of the apo form of HasAyp. In red is represented the first cluster, and the remaining clusters are in gray.

The visual inspection of the trajectory and clusters of the GaMD simulations revealed how the tertiary structure of the protein, the β-sheet, and α-helices displays very little conformational changes (Figure 3). The loop and hairpin regions only show some degree of flexibility, as confirmed by the RMSF analysis (Figure S2) in particular hairpin H3 (average RMSF 2.86 Å) and loop L1 (average RMSF 1.87 Å). The latest oscillated during the simulation around its resting position although with only small changes in its rearrangement and could partially acquire the conformation of a small turn or helix.

**Figure 4:**
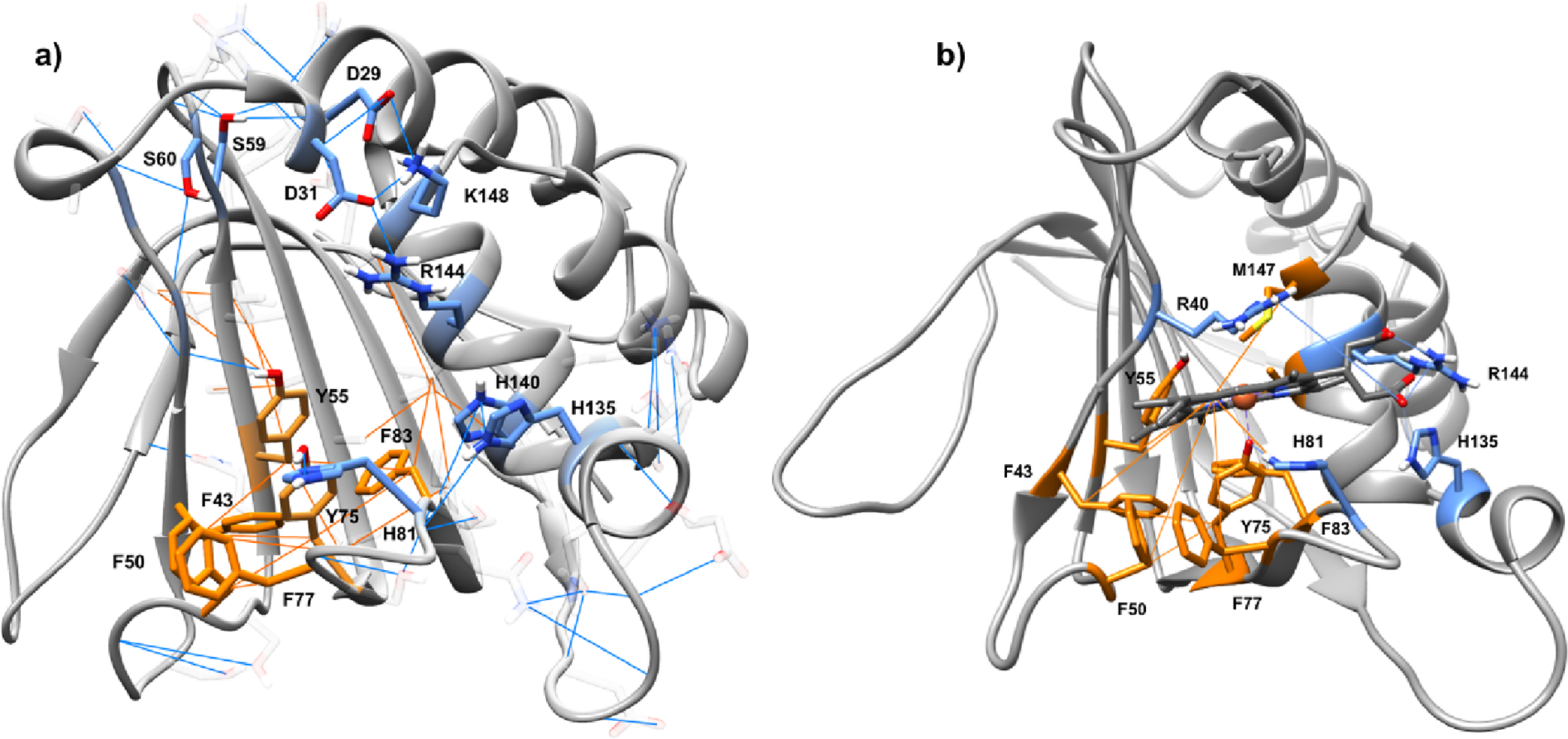
Representation of interactions during GaMD simulations in HasAyp. Hydrogen bonds in blue and hydrophobic contacts in orange with main residues involved highlighted. (a) Interactions extracted from GaMD apo from HasAyp. (b) Interactions between heme and HasAyp extracted from GaMD holo.

Only in very brief sections of the trajectory, loop L1 reaches either a more closed arrangement for which it could be foreseen that the heme could not be bound or a slightly opened but never reaching the open conformation observed in the structure of HasA of *S. marcescens*. In this “closed” arrangement, hydrophobic interactions and hydrogen bonds were observed during the whole simulation (>75%) in between the α-helices and β-sheets. Interestingly, a network of several π-contacts between aromatic residues of the pocket (Tyr55, Phe43, Phe83, Tyr75, Phe50, and Phe77) maintains the heme-binding region in a closed conformation in the absence of heme (Figure 4a). These interactions also may contribute to keeping the stability of the overall protein structure with almost no structural changes. When looking more in detail at loop L1, several interactions were identified as responsible for maintaining it in the crystallographic arrangement and preventing it from reaching an open form. Stable salt bridges and hydrogen bonds between residues Lys148 and Arg144 from helix-α A2 and residues Asp29 and 31 situated at the Nter of loop L1 (Figure 4a) were found during the entire simulation trajectory. Furthermore, 25–50% of the GaMD trajectory, hydrogen bonds between polar residues of loop L1 (Lys38, Arg40, Ser60), and the backbone or side chains of the same loop were found. Overall, the hairpin H3 was found to be the most flexible region in this hemophore due to the lack of strong intra-protein interactions.

To gain further insights into the heme-binding process, protein–ligand docking of the heme cofactor was performed using the most representative protein conformations of the GaMD trajectory. The calculations were performed with the updated GoldScore scoring function for metalloligands.^31^ Taking into account that the GaMD of the apo-form shows substantial similarity with the holo structure available in the PDB, it was not surprising that all dockings showed excellent scoring values for heme binding (ca. 90 GoldScore units). The resulting complexes showed a structural arrangement similar to the experimental structure, including the presence of a coordination bound between the metal and the Tyr75 in all the cases.

Finally, simulations with the heme bond were performed to ascertain how stable the heme–HasAyp complexes predicted in the dockings are. We envisioned that some of those complexes may differ from the X-ray structure and expected to see how the structure of the protein is affected by the binding of heme. Three replicas of GaMD simulations starting from the heme-docking position were undertaken.

**Figure 5:**
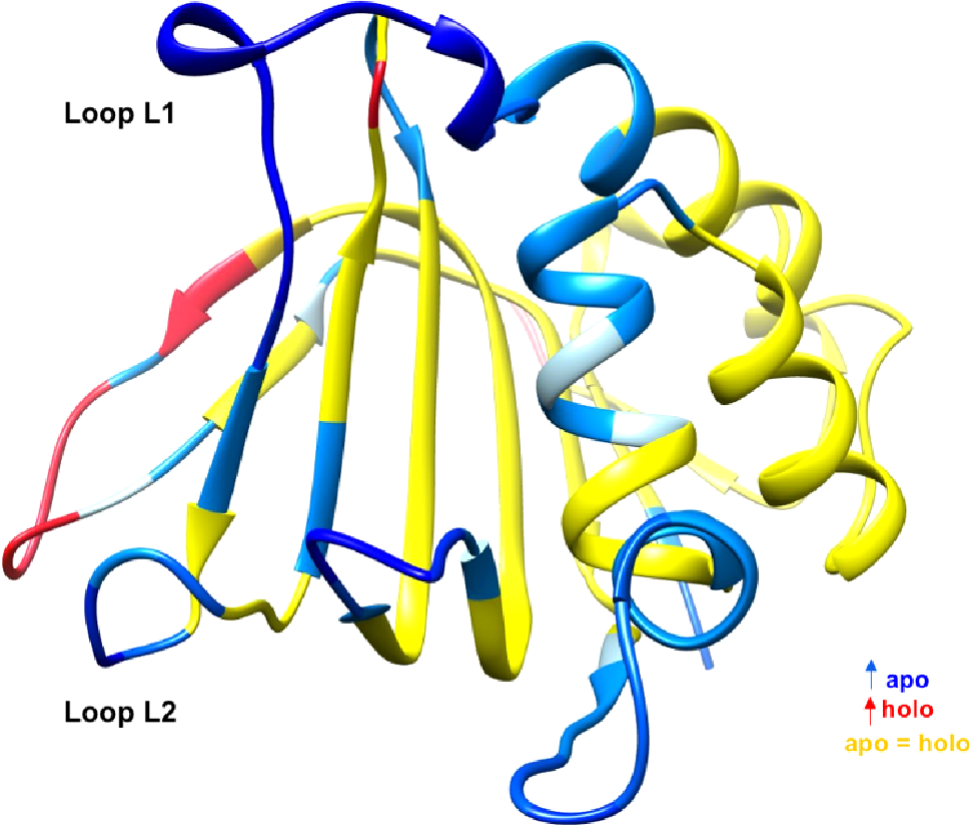
RMSF difference between apo and holo from GaMD of HasAyp. In blue, RMSF is higher in apo, and in red, RMSF is higher in holo. No significant RMSF differences are shown in yellow.

The analysis shows that the theoretical holo forms of HasAyp do not present significant conformational changes. The system tends to reach convergence only after 100 ns as demonstrated by the stability of rmsd, cluster counting, and PCA analysis (Figures S3–S6). Neither the entire tertiary structure nor secondary motives, including loop L1, presented significant variations. In general, the core structure of the protein showed again low flexibility and very high stability, finding the only differences in loops L1 and L2 and the helix α-2 (Figures S4 and S6). These appeared more flexible in the apo form than in the holo form, loop L1 being the one that showed the highest flexibility (average RMSF difference of 0.79 Å) (Figures 5 and S7). It can be concluded, therefore, that the presence of heme restricts the movement of loop L1. Interestingly, the only region which was found to be slightly more flexible in the heme bound form was the hairpin H3 (average difference RMSF 0.13 Å).

We ended our analysis comparing the network of interactions of the amino acids between the holo and apo forms. Although most hydrogen bond interactions are preserved in both systems, the holo form shows less frequent interactions inside loop L1 and between the loop and the protein than the apo form because these residues now interact with heme. Interestingly, despite the lack of coordination of the heme to any residue of L1, strong interactions appear between the prosthetic group and the loop, hence reducing its flexibility. Heme presents the main hydrogen bond and salt bridge interactions between the propionates of the heme and Arg144, Arg40, and His135 (Figure 4b). Since these interactions are maintained during the majority of the simulations, these may be responsible for maintaining loop L1 in a closed disposition even when heme is bound. In addition, the hydrophobic interactions found in the apo system involving Phe43, Phe83, and Tyr55 now interact directly with the heme. However, these do not seem to affect the loop L1 region.

Altogether, these calculations have revealed that the hydrophobic residues Tyr55,75 and Phe43,83,50,77 of the binding site are a platform for the binding of heme, while positively charged residues Arg144,40 and His135 stabilize through salt bridges and made propionates face the solvent.

### HasA from *S. marcescens*

In contrast to HasAyp, the apo form of HasAsm has a completely different conformation of loop L1, presenting an open conformation in the heme-free structure (Figure 2a). We aimed at assessing the nature of the interactions occurring in both the holo and apo forms of HasAsm and understanding the differences for heme uptake with respect to the HasAyp system. Following the same protocol, GaMD simulations were carried out on the apo form of HasAsm. Because His32, which is the heme axial ligand, could potentially be in different protonation states influencing the flexibility of loop L1, simulations were performed with different protonation states of this residue. No significant differences were observed, and here, the system with His32 at the neutral state with monoprotonation at Nδ will be described. The PCA and clustering analyses showed that the GaMDs converged by the end of the 800 ns (Figure S8).

**Figure 6:**
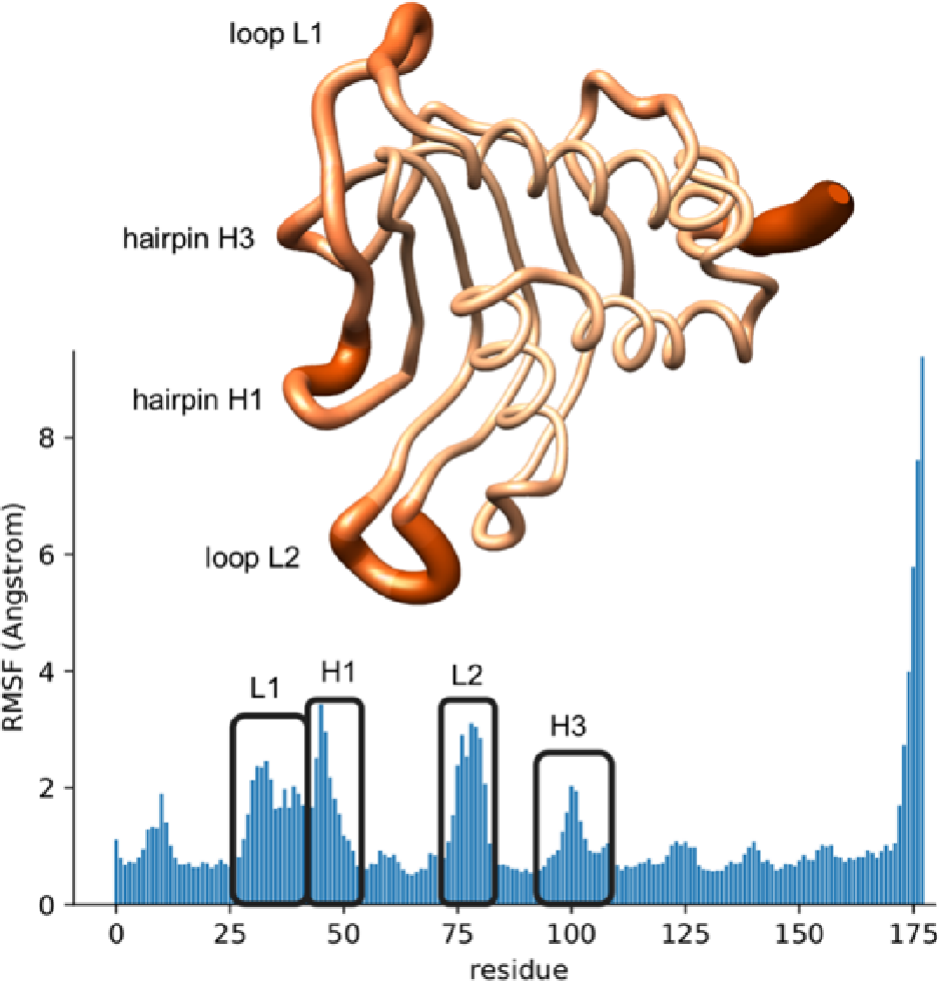
RMSF of apo GaMD from HasAsm with relevant regions.

No major conformational changes were observed in the α-helix or β-sheets of the system. However, some variations were observed in the loop and hairpin regions. The RMSF analysis showed the highest values for loop L1, L2 and hairpin H1, H3 (Figures 6 and S9). Even though the flexibility of loop L1 is quite high (average RMSF about 1.84 Å), no major movement of loop L1 was observed in any of the replicas. Because loop L1 always remained close to the initial open conformation, simulations did not show the transition of loop L1 toward more closed conformations. Therefore, nothing in the three converged replica simulations of 800 ns suggests that the apo form of HasAsm could naturally move toward a more closed conformation without the presence of the heme cofactor.

**Figure 7:**
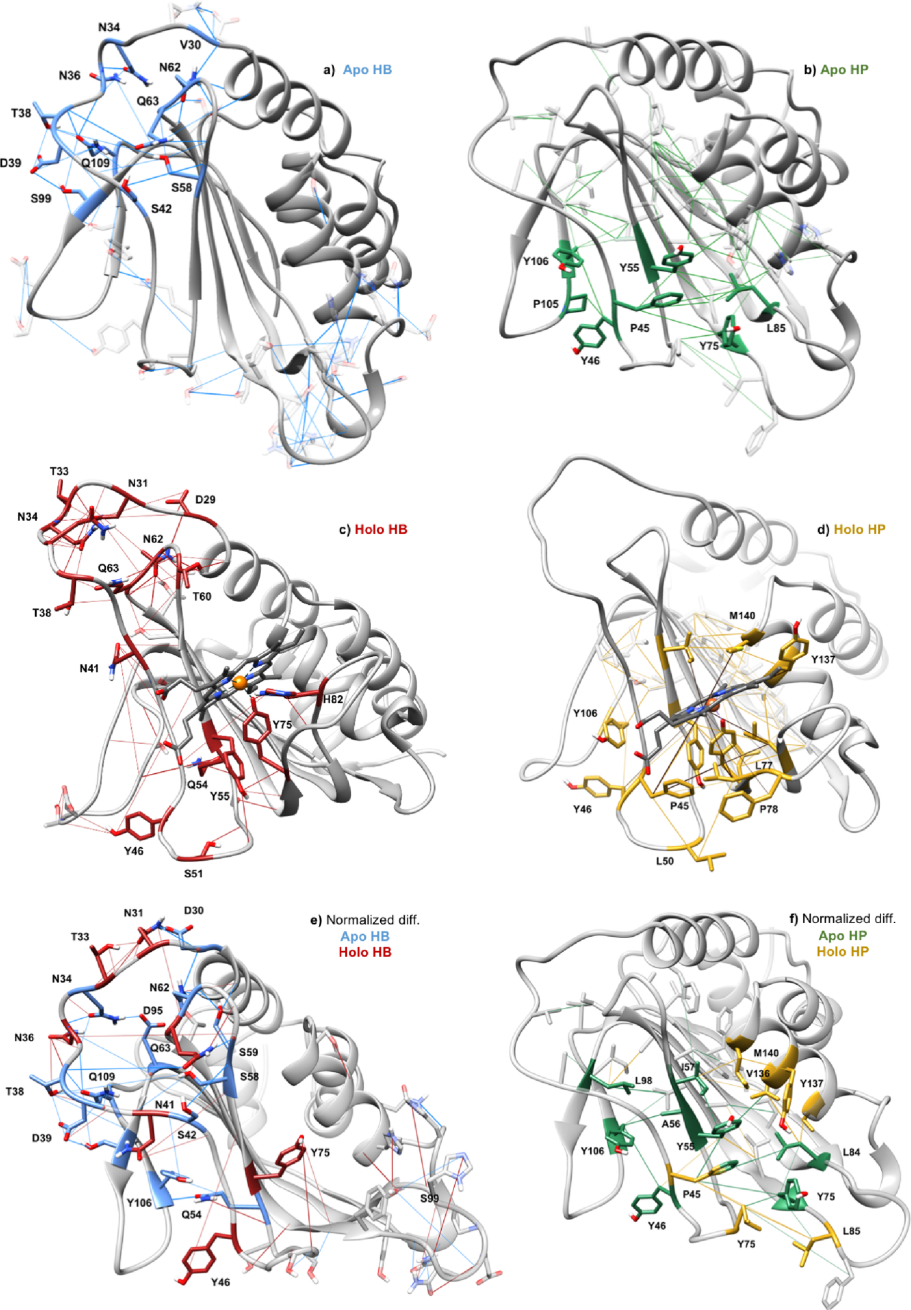
Representation of hydrogen bonds (HB) and hydrophobic (HP) interactions during GaMD of HasAsm in apo (a,b) and heme-bound before loop closing (c,d). Normalized difference between apo and holo forms of both hydrogen bonds (e) and hydrophobic interactions (f) is represented. See Figure S15 for Fe(II) analysis.

To better understand why L1 is not able to escape its open conformation and reach heme-bound like states, an analysis of the interactions during the GaMD simulations was carried out (Figure 7a). First, a network of hydrogen bonds can be observed between loop L1 and three other regions: (1) its Nter part is fixed by interactions between Val30, Asn36, and Thr38 with hairpin H2 (Asn62 and Gln63); (2) central residues Thr38 and Ser39 interact with β-sheets B5-6 (Ser99 or Gln109), which keeps the most flexible part of the loop fixed; and (3) its Cter has interaction of the backbone 40–44 with β-sheet B3 and the side chains of Ser42 with Ser58–59. All interactions keep loop L1 fixed close to the initial position and therefore in apo-like conformations. This is reinforced by a network of hydrogen bonds in the heme-binding regions involving histidine 83, 128, and 133. Regarding the hydrophobic interactions, in harpin H3, there are π–stacking interactions that stabilize close contacts with β-sheets B2–3 and loop L1 (Figure 7b). Those involve mainly Tyr46 and Ala56 from β-sheet B2 that interact with hairpin H3 or β-sheets B5-6 with Pro105, Tyr106, and Leu98. However, the most relevant hydrophobic interactions in the system are not related directly to loop L1 but are part of the heme-binding region. Importantly, there are several π–stacking contacts between the aromatic residues Phe45, Tyr75, Leu85, and Tyr55, making a very robust network of interactions.

As the GaMD simulations of the apo-HasAsm did not show any conformational changes of loop L1 consistent with the heme-bound structure, we hypothesized that it may have been induced by the binding of the heme. We therefore pursued with heme dockings followed by GaMD simulations. Similar to what is observed in HasAyp, docking predicted good binding affinities (GoldScore values of ca. 68) and similar binding poses as in the X-ray structure of the heme-bound form, showing a coordination bond between the heme and the oxygen of the side chain of Tyr75. Surprisingly, the best docking solutions showed that the propionate groups were facing the inner part of the protein, and it was only after an equilibration with classical MD simulations of 10–20 ns that the heme rotated toward the external part of the binding pocket and ended with a structure with excellent matching with the experimental holo form. These results highlight that binding of the heme to HasAsm could perfectly happen without the necessity of loop L1 to transit to the X-ray position and the His32 to coordinate the remaining axial site. These simulations were followed by three replicas of 800 ns GaMD simulations.

**Figure 8:**
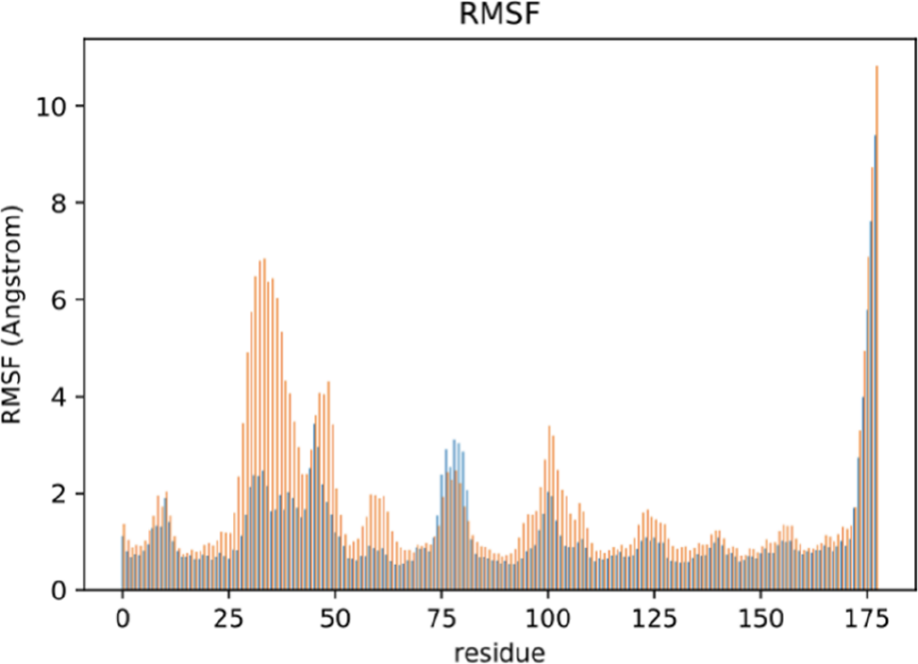
RMSF difference between apo in blue and holo in orange extracted from apo and holo GaMD of HasAsm.

All the statistical indicators on the GaMD replica simulations (clustering, PCA, rmsd) revealed convergence after 100–400 ns (Figures S10–S13). Several wells found in the PCA analysis showed that the system visited distinct conformations. In particular, the RMSF analysis showed that the most flexible part of the protein corresponded to the loops and hairpin regions, loop L1 being the most flexible one (Figure S13). When comparing the RMSF with the apo form, once again a rigid core structure of the protein was observed with no changes in the tertiary structure, except for loop L1 (RMSF diff. of 4.1 Å) and the β-sheets B3 (RMSF diff. 0.98 Å) (Figures 8 and S14). Only loop L2 is more flexible in the apo form. This result sustains that the binding of heme, with extremity ends at the opposite side of the protein and in its pocket, induces a conformational change at loop L1, initiating its closing mechanism.

**Figure 9:**
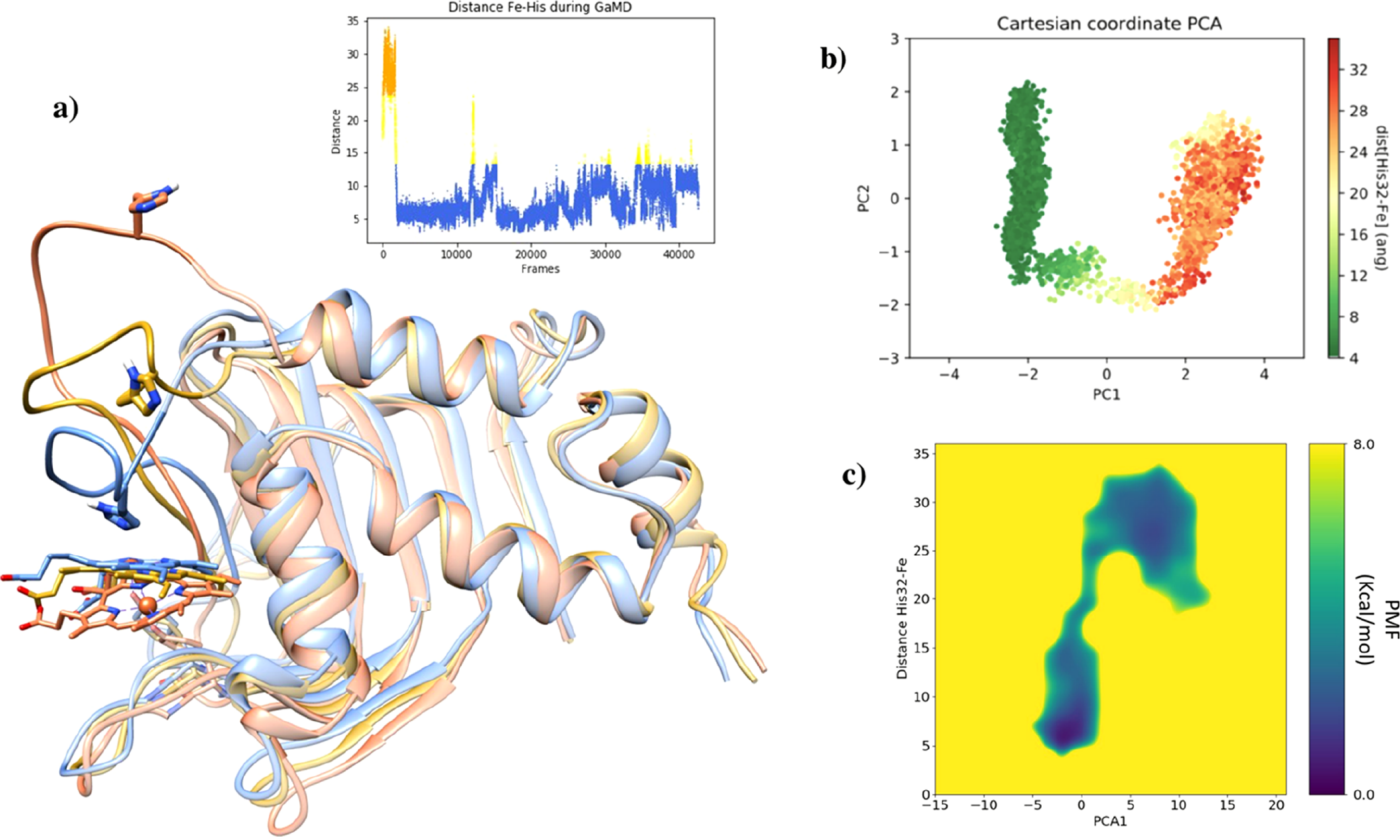
GaMD simulation of HasAsm with heme–Fe(III) bound. (a) Frames of GaMD showing the loop L1 closing process colored according to the distance between Fe and His32 during GaMD. Graphic of distance Fe–His32 represented. (b) Cartesian coordinate PCA analysis colored according to the distance between Fe and His32 during GaMD. (c) Reweighted PMF calculations in front of PCA1 and distance Fe–His32. (b,c) are obtained using the fragment of the trajectory in which the loop is closing.

Visual inspection of the trajectory of the three GaMD replicas shows a similar mechanism for loop L1 closing. Detailed analysis shows that at the beginning of the GaMD simulation, loop L1 tends to acquire an open conformation; it has separated from the region of hairpin H3 and β-sheets B5–6. Only after 40 ns up to 700 ns, depending on the replica simulation, the loop adopts a turn or small helix conformation and moves toward the heme-binding site and acquires a more closed disposition (Figure 9a).

For the rest of the GaMDs, the loop oscillates between different conformations, with some of them consistent with His32 facing the heme at distances consistent with coordination to the metal, while in others, the nitrogen faces outside of the binding site. Despite the closing motion of the loop L1 appears once the heme has bound to the HasAsm apo form, a stable holo structure with His32 as the 6th ligand of the iron is not maintained in the simulations. The distance of the coordinating nitrogen of His32 to the iron tends to swing between 3 and 12 Å depending on the replica with no stable position of His32 in a coordination mode. This is due to the force field conditions that only allow one coordination state at a time, and these simulations start with the sole Tyr75 bound and the corresponding parameters of a pentacoordinated first coordination sphere. Although some Fe(III) simulations show shorter distances than in Fe(II) calculations, this occurs very sporadically and indicates a limited impact on the net charge of the metal, and the absence of an explicit Fe–His bond in the parametrization is the origin of the fluctuation. To further investigate this point, additional GaMD simulations were performed. On one side, the holo X-ray structure (PDB code 1DKH) was submitted to GaMD simulations, with parameters for the Fe–Tyr coordination and no coordination term for the Fe–His bond. The histidine flies out of the heme binding pocket and fluctuates around 7.43 Å (going from 2.7 to 17.9); however, loop L1 remains mostly in a closed geometry (Figure S16). On the other hand, simulations starting from the snapshot of the GaMD of the HasAsm with the heme bound to the tyrosine and the smallest Fe–O_Tyr_ distance (2.9 Å) were performed using a set of parameters for the hexacoordinated metal with axial His-Fe-Tyr configuration. This simulation rapidly reaches a loop L1 conformation close to the experimental holo form (rmsd of 0.78 Å), and a stable His–Fe bond is observed during the simulation (Figure S16). This shows that reaching the final hexacoordinated structure requires the His coordination to be properly modeled.

Still, the major determinants of the loop closure motion are the fluctuations of the protein once the heme has bound its cavity. PCA analysis of fragment trajectory where loop L1 closes with respect to the Fe–His32 distance reveals how PC1 involves the movement of loop L1 (Figure 9b), and reweighted PMF calculations in front of the PCA1 distance Fe–His32 shows that the barrier of the system to transit from one state to another is less than 8 kcal/mol (Figures 9c and S17 and S18).

Analysis of interactions reveals how the binding of the heme clearly induces some changes in the network of hydrophobic and stacking interactions (Figure 7c,d). Basically, the binding disrupts all the previously mentioned π–stacking and hydrophobic interactions between aromatic residues of the heme binding site (Tyr75, Tyr137, His83, Tyr55, and Phe45). Instead of interacting between them, these residues now interact with the heme. Furthermore, the interaction between Tyr46 and Tyr106 decreases, allowing much more flexibility of hairpin H3 that separates from β-sheets B2–3 and from loop L1. This affects loop L1 and its hydrogen bond interactions; during the first half of the simulations, all hydrogen bonds between L1 and hairpin H3 drastically decrease, making it more flexible. Loop L1 can make more hydrogen bonds with β-sheets B5–6 and hairpin H2 (mainly with Thr60, Asn62, and Gln63). As mentioned before, the binding of the heme decreases the π–stacking interactions between Phe45 from loop L1 and the residues of the heme binding site; therefore, Phe45 can interact with residues Leu50 and Leu77 from hairpin H1. This change of interactions induces conformational changes in the region of hairpin H1 and Cter of loop L1. This could be the cause of the appearance of a turn or semi-helix in disposition on loop L1. At this point, the previously mentioned interactions with β-sheet B3 and H2 are substituted by a series of hydrogen bond interactions inside the same loop L1. This change also causes the separation of loop L1 from β-sheets B5-6, and loop L1 starts to move toward the heme-binding site, while at the same time, α-helix A1 moves toward α-helix A2. From this point, where the loop L1 is in closed disposition, there are a series of hydrogen bond interactions between loop L1 and α-helix A2 that maintain it in a close conformation. The main residues involved are Asn31 and Val30 from loop L1 with Ser141 from α-helix A2. If we compare the frequency of all these interactions with the apo form and normalize it, these tendencies can be clearly observed in Figure 7e,f.

### In-Depth Comparison between HasA from *Y. pestis* and *S. marcescens*

In this study, the combination of GaMD and docking highlights differing patterns of heme-binding mechanisms between the hemophores from *Y. pestis* and *S. marcescens*.

Simulations on the HasAyp clearly demonstrate that the apo form remains in a geometry similar to that of the holo form. The transition between a close and open conformation resulting from the movement of loop L1, as described in HasAsm experimental structures, is never observed despite GaMD simulations allowing extensive conformational sampling. Calculations show that this is due to specific salt bridges and hydrogen bonds between negatively charged residues of loop L1 (mostly Asp30 and Asp31) with positive residues from helix from the core (the most important ones being Lys148 and Arg144). The binding of the heme only slightly impacts some hydrophobic interactions but not sufficiently to disrupt the geometry of loop L1. It can be concluded that the mechanism of heme-binding would correspond to a very light conformational selection. This part of the study shows that the HasAyp hemophore presents a well pre-organized geometry of the receptor for heme binding as a classified transient heme-binding protein.

Simulations on HasAsm also show that both apo and holo structures are very stable. Interestingly, no structural rearrangement of loop L1 is observed in any of them. Simulations on the apo form show that the binding site of the heme is well pre-organized and that binding should occur naturally. Moreover, the motion of loop L1 and the coordination of His32 have no impact on the insertion of the heme in its cavity or the coordination of the oxygen of the side chain of Tyr75 to the heme. As a crucial result of this study, the movements of loop L1 are only observed after the heme process takes place. Analysis of contacts shows that the binding of heme disrupts the network of hydrophobic and π–stacking interactions at the heme-binding site and that this information is propagated to the other extreme of the protein. This change induces a conformational change in hairpin H3 that acquires more flexibility and loses interactions with residues of loop L1. This also leads to a disruption of the interactions between the C_Ter_ part of loop L1; both hydrophobic and hydrophobic contacts with hairpin H2 and β-sheets B5–6 are broken, which causes loop L1 to be more flexible. Once L1 has been liberated from this strong interaction, it becomes free to reorganize and move up to the heme-binding site. This ultimately leads to the correct organization of the loop so that His32 could find the heme and coordinate.

This mechanism sustains that the general transfer of structural information along the structure of the protein is similar to those observed on hemophore HasAp using targeted dynamics. In particular, it suggested that the interaction between residues from helix A2 with heme induces a tilting motion that perturbs interactions with helix A1 and loop L1, initiating its closing movement. Compared to previous studies, we are here able to simulate the transition from apo to holo without forcing or constraining the system. Long GaMD simulations of 800 ns simulations have been performed, which have allowed all systems to converge and observe significant conformational changes. Furthermore, performing three replicas for each case assured that these events are not casual. The conformational changes of L1 observed in the experimental structures of the apo and holo forms are, in reality, a subsequent process where long-range interactions are involved as a transmitted signal from the heme-binding pocket and can ultimately lead to an additional coordination with respect to other hemophores.

## Conclusions

In this study, we investigated the heme-binding mechanism of two hemophores, one from *Y. pestis* and the other from *S. marcescens*. Despite similar fold and biochemical functions, the former presents little conformation changes between apo and holo forms, while the latter presents an important conformational change characterized by a large movement of one loop, which ends in the formation of an additional coordination bound between an axial histidine and the metal. To decode the origin of such differences, we combine molecular dynamics experiments with protein–ligand dockings. We take advantage of the extensive conformational sampling that Gaussian accelerated molecular dynamics offer to ascertain if the receptors could easily reach sub-states compatible for heme-binding and unbinding processes as well as docking techniques that have been optimized to deal with metalloligands.

From this study, it can be concluded that in all cases, the apo forms are very stable. Loop L1 is kept fixed due to a strong network of hydrogen bonds with amino acids of surrounding loops and β-sheets. These unbound forms are also well pre-organized for the binding of the heme. In both cases, the binding of the heme occurs without any previous major conformational changes including the loop transition. The apo is therefore very well pre-organized for heme binding.

The main differences between both species come from the cascade of molecular events happening after the heme binds and the conformational changes associated to them. While very little perturbation of the overall map of contacts and interactions happens in *Y. pestis*, a series of changes take place in *S. marcescens*. The mechanism starts with modifications of hydrophobic contacts in the heme-binding pocket followed by rearrangements of hairpin and loop contacts on the opposite side of the protein that end up modifying the patch of the interactions that loop L1 is part of. As such, L1 acquires far wider flexibility. This ultimately leads to conformations where the coordinating histidine reaches the heme-binding pocket and forms an additional bond with the iron.

This study shows interesting information on heme recruitment by different families of hemophores. In all cases, it would appear that the good pre-organization of the heme pocket for the binding of the cofactor is consistent with transient mechanisms. However, for the *S. marcescens*, there is a subsequent induced fit effect that allows the system to reach its final holo that ultimately leads to an additional coordination bond. How the additional step observed in HasAsm could be an advantage or inconvenient for the organism from the evolutive point of view remains unclear. However, this study completes our knowledge on heme-binding complexity and how long-range interactions could be crucial for defining the heme-bound geometry and the mechanism of acquisition. In an age of increasing interest in the insertion of metal-based compounds into protein scaffold, this study highlights the importance of weighing pre-organization and induced fit effects, understanding their origin, and predicting their magnitude.

## Supporting Information Available

Additional conformational and explorational analysis of all GaMD simulations, interaction analysis, and force field parameters for heme groups (PDF)
