# Supplementary material for: Getting Deeper into the Molecular Events of Heme Binding Mechanisms: A Comparative Multi-level Computational Study of HasAsm and HasAyp Hemophores
Source: Inorg Chem. 2022 Oct 17;61(43):17068–79. doi: 10.1021/acs.inorgchem.2c02193 (PMC9627568; doi:10.1021/acs.inorgchem.2c02193)
Supplement: Supplementary file 1 — ic2c02193_si_001.pdf [file ic2c02193_si_001.pdf]

Electronic Supplementary Information for:

Getting deeper into the molecular events in heme binding mechanisms: A comparative multi-level computational study of HasAsm and HasAyp hemophores

Laura Tiessler-Sala,[1] Giuseppe Sciortino,[1,2] Lur Alonso-Cotchico,[1,3] Laura Masgrau,[1,3] Agustí Lledós,[1] Jean-Didier Maréchal[1*]

[1]Insilichem, Departament de Química, Universitat Autònoma de Barcelona, 08193 Bellaterra (Barcelona), Spain
[2] Institute of Chemical Research of Catalonia (ICIQ), The Barcelona Institute of Science and Technology, 43007 Tarragona, Spain
[3] Zymvol Biomodeling, carrer Roc Boronat 117, 08018 Barcelona, Spain

Corresponding author e-mail: jeandidier.marechal@uab.cat

Table of contents

**1.** Conformational and explorational analysis of HasAyp GaMD simulations	S2
**2.** Conformational and explorational analysis of HasAsm GaMD simulations	S9
**3.** Interaction analysis of HasAsm GaMD simulations for Fe(II)	S16
**4.** GaMD analysis of holo X-ray	S17
**5.** Analysis GaMD: Distance Fe-His, PCA and energetic analysis	S18
**6.** Force field parameters for heme	S20

1. Conformational and explorational analysis of HasAyp GaMD simulations

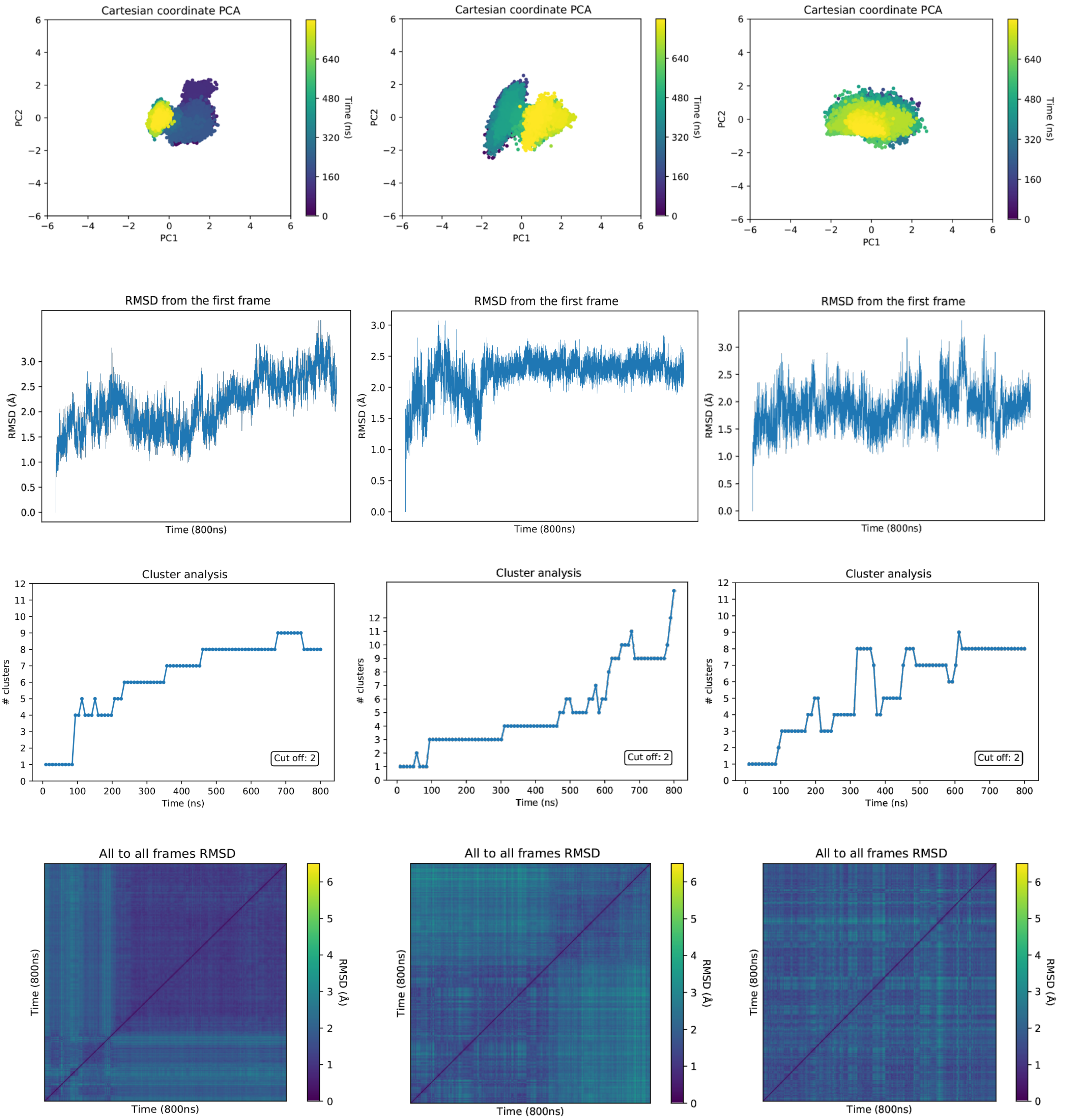

SI Figure 1: GaMD convergence analysis of HasA apo form of *Yersenia pestis* (800ns – three replicas): RMSD, all-to-all RMSD, PCA and cluster counting.

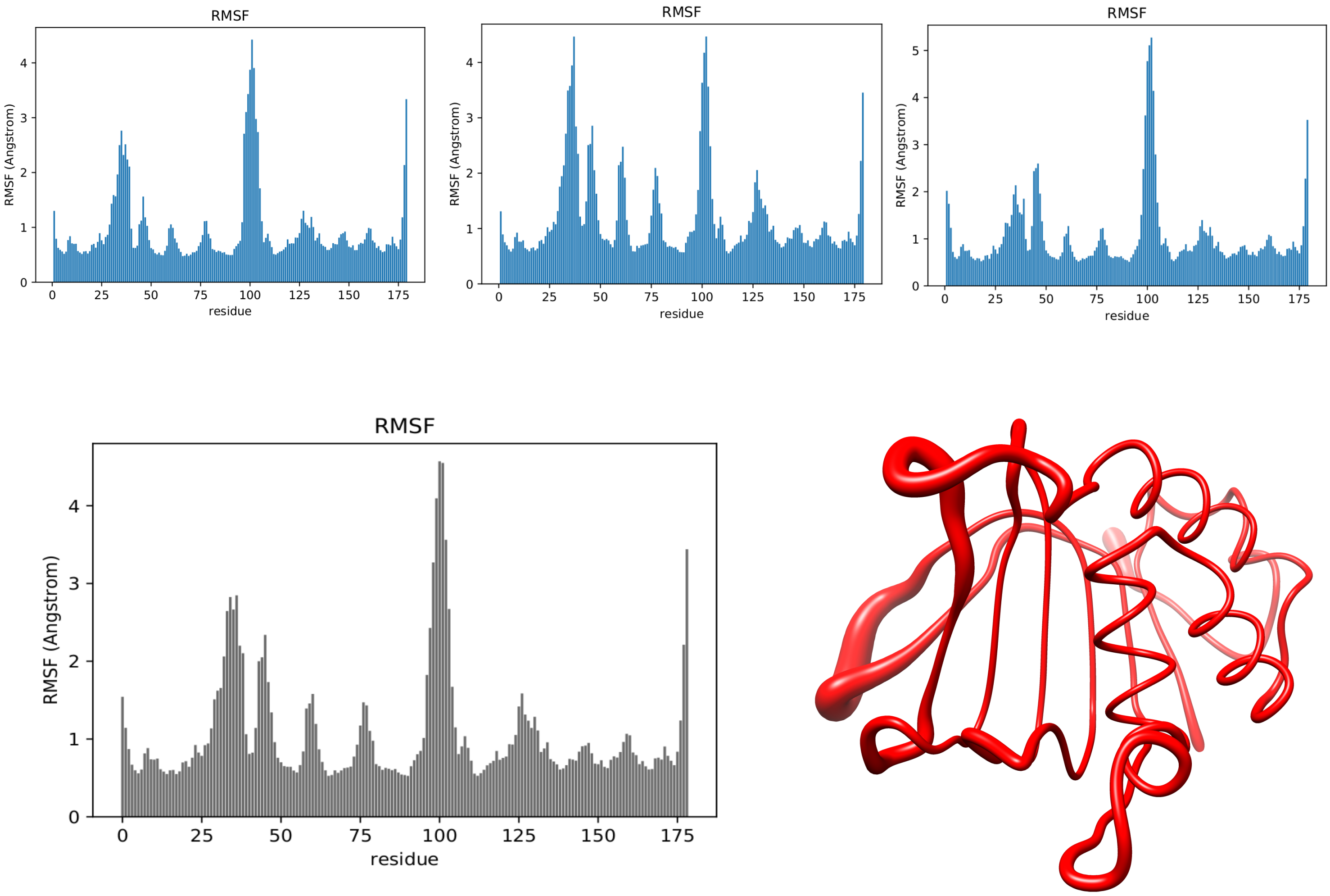

SI Figure 2: RMSF flexibility analysis of HasA apo form of *Yersenia pestis* (three replicas). Average RMSF across 3 replicas and representation into structure of HasAyp apo.

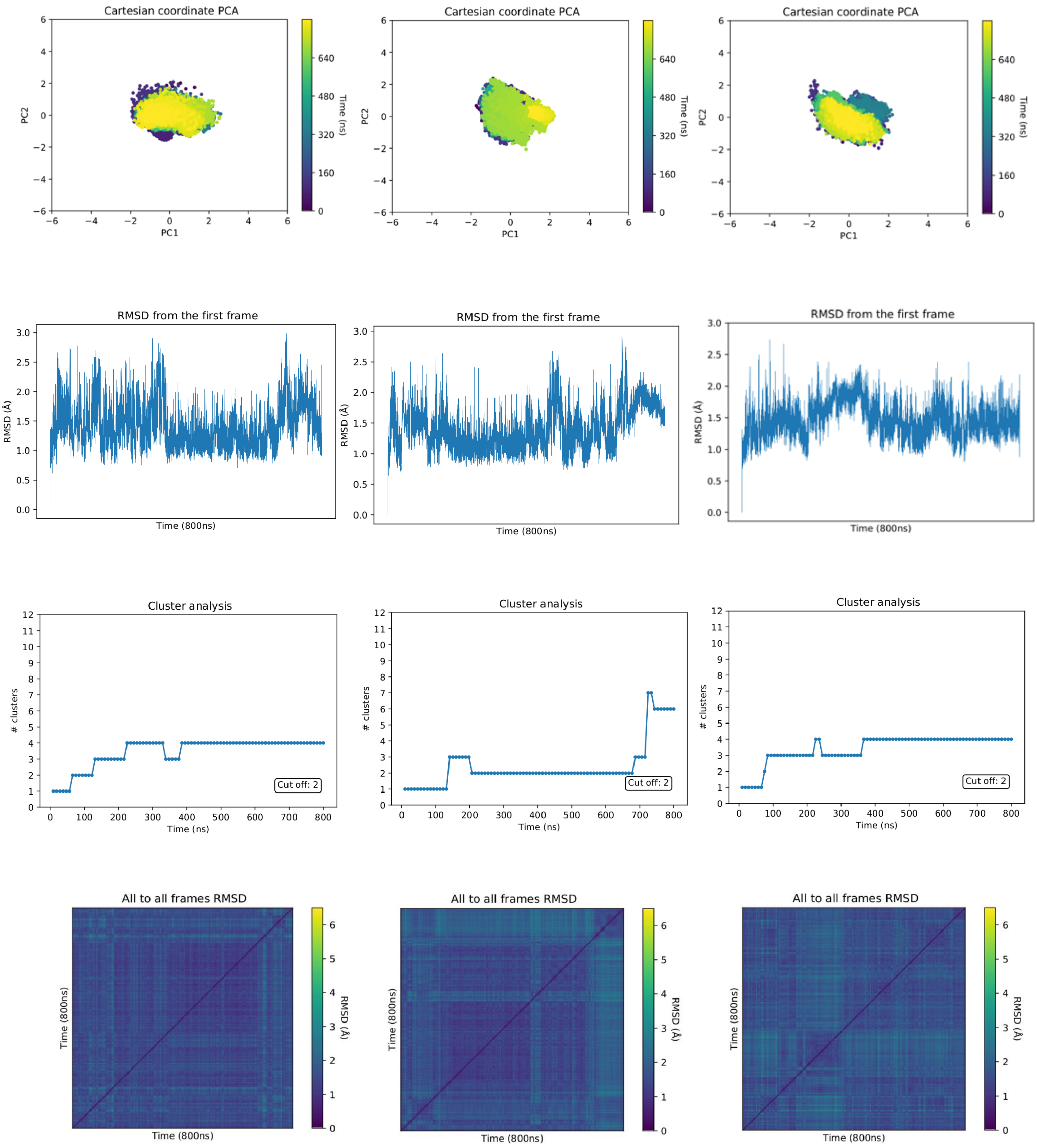

SI Figure 3: GaMD convergence analysis of HasA heme-Fe(III)-bound form of *Yersenia pestis* (800ns): RMSD, all-to-all RMSD, PCA and cluster counting.

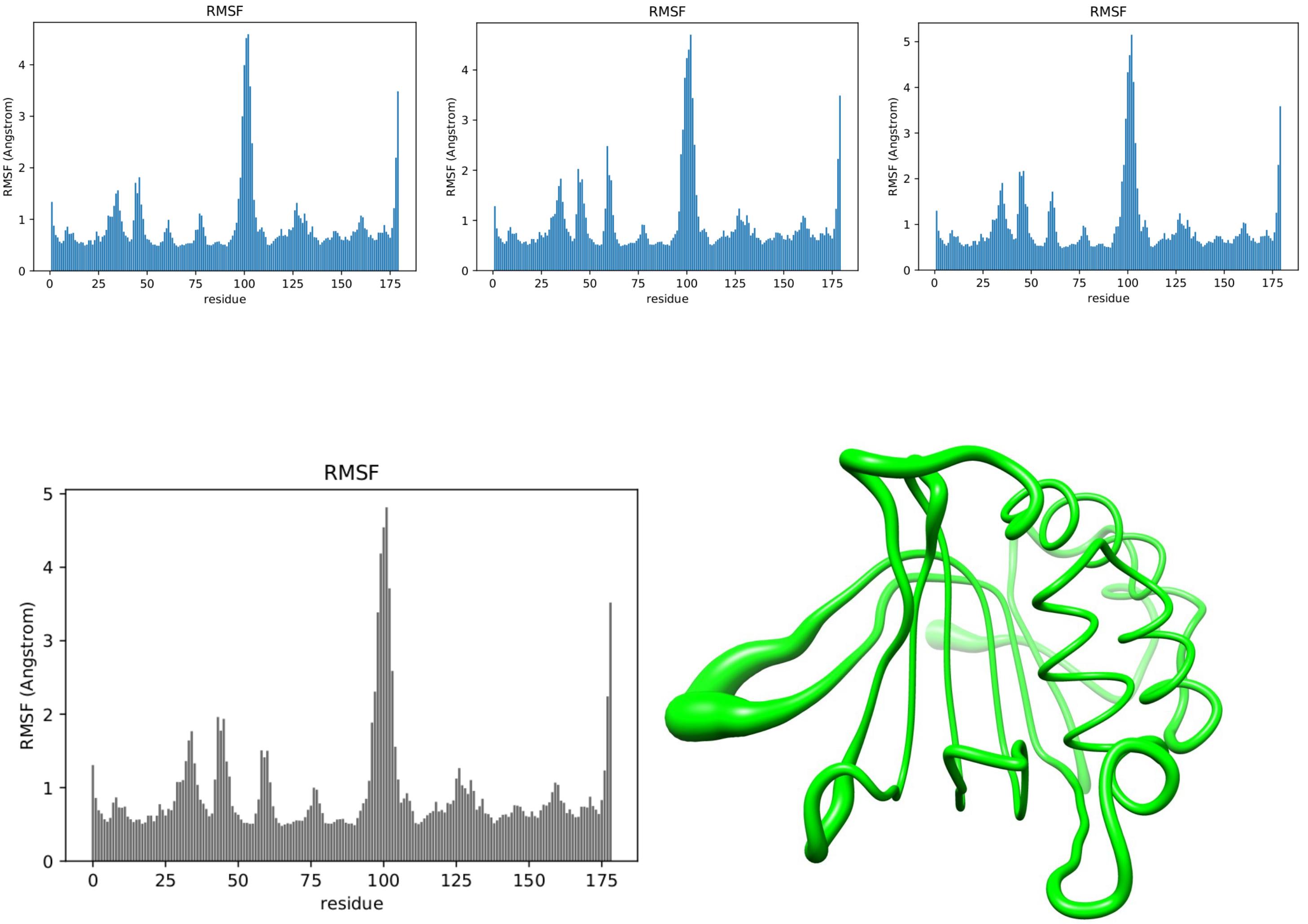

SI Figure 4: RMSF flexibility analysis of HasA heme-Fe(III)-bound form of *Yersenia pestis* (three replicas). Average RMSF across 3 replicas and representation into structure of HasAyp holo form.

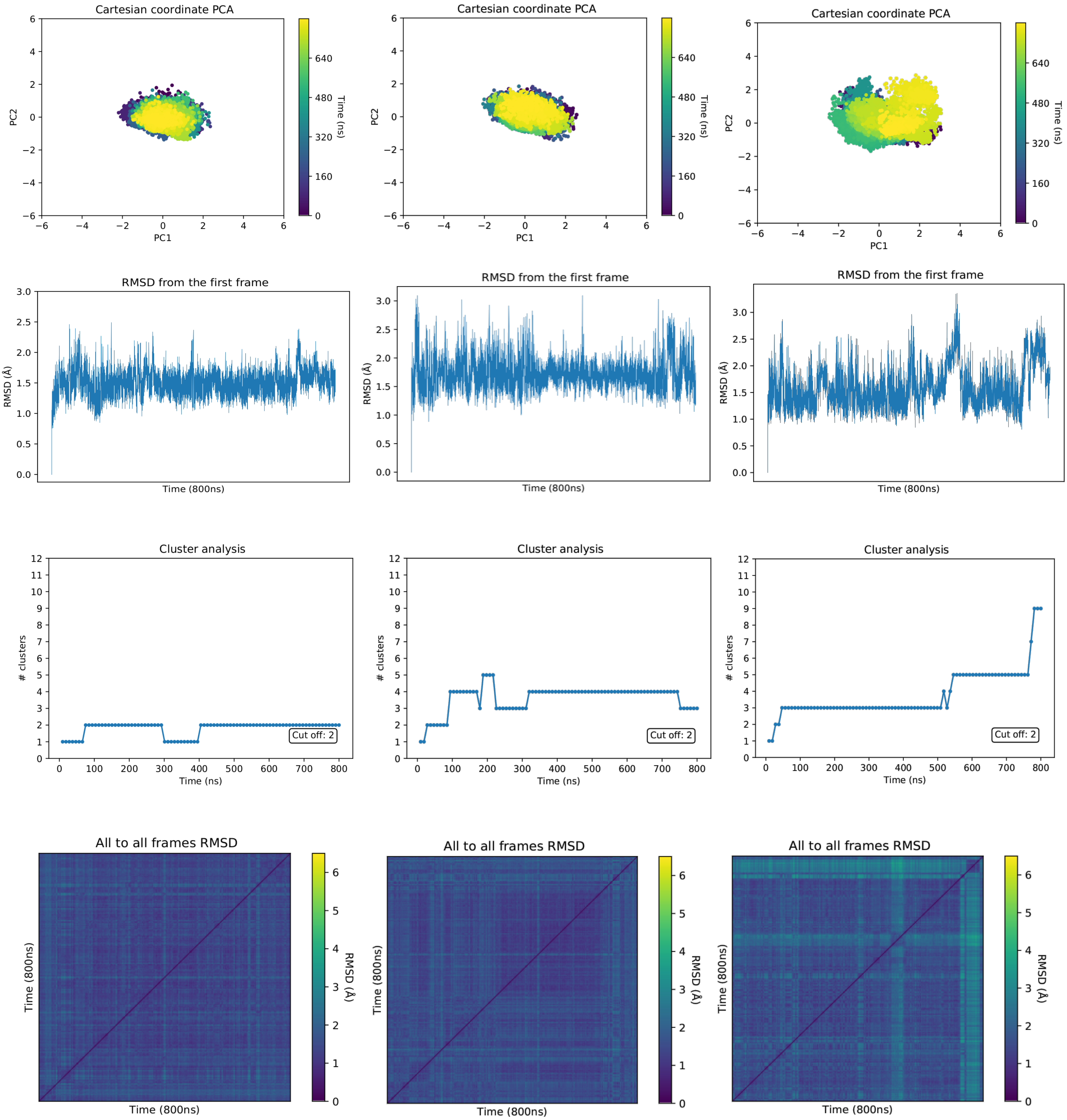

SI Figure 5: GaMD convergence analysis of HasA heme-Fe(II)-bound form of *Yersenia pestis* (800ns): RMSD, all-to-all RMSD, PCA and cluster counting.

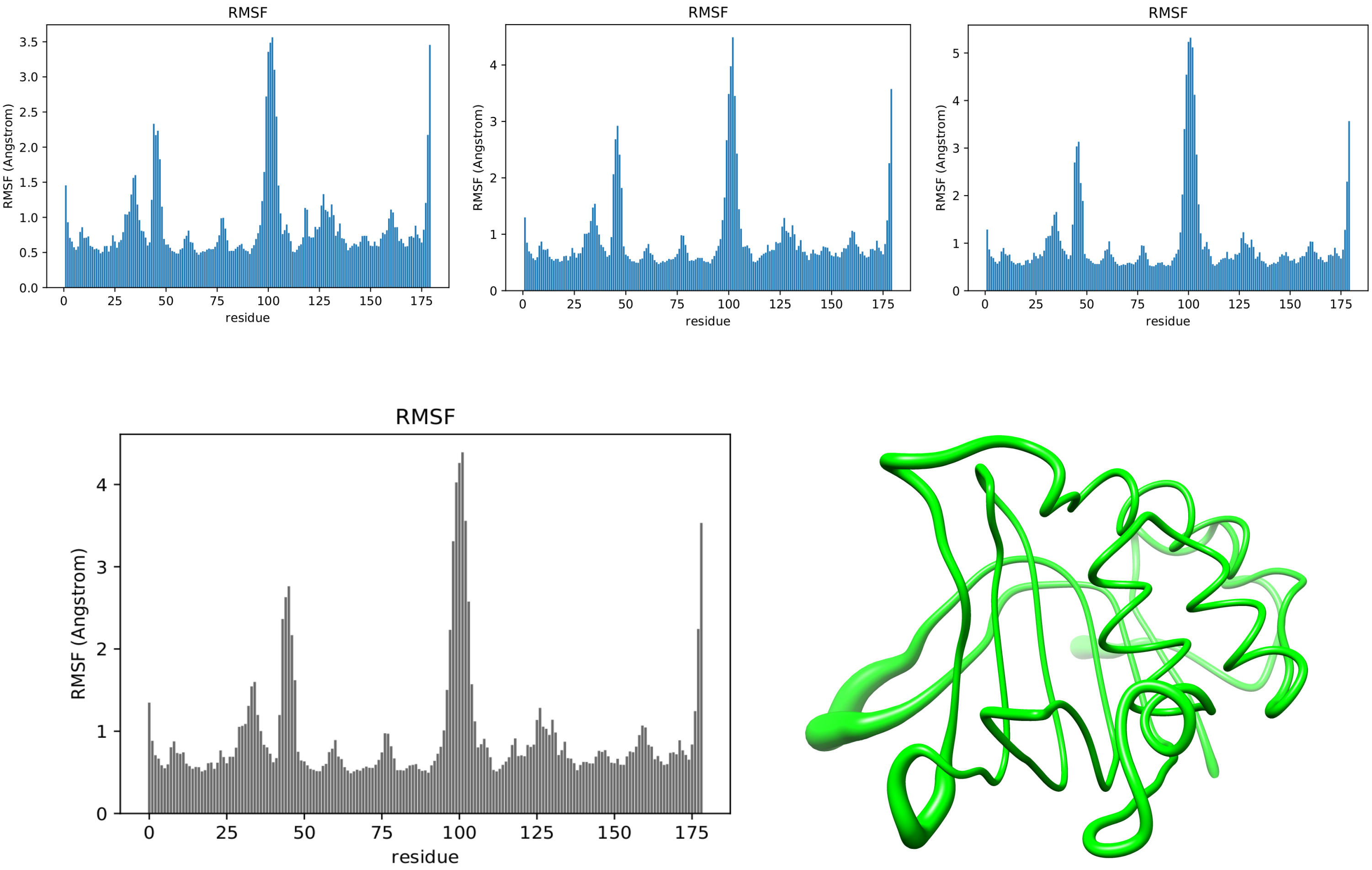

SI Figure 6: RMSF flexibility analysis of HasA heme-Fe(II)-bound form of *Yersenia pestis* (three replicas). Average RMSF across 3 replicas and representation into structure of HasAyp holo form.

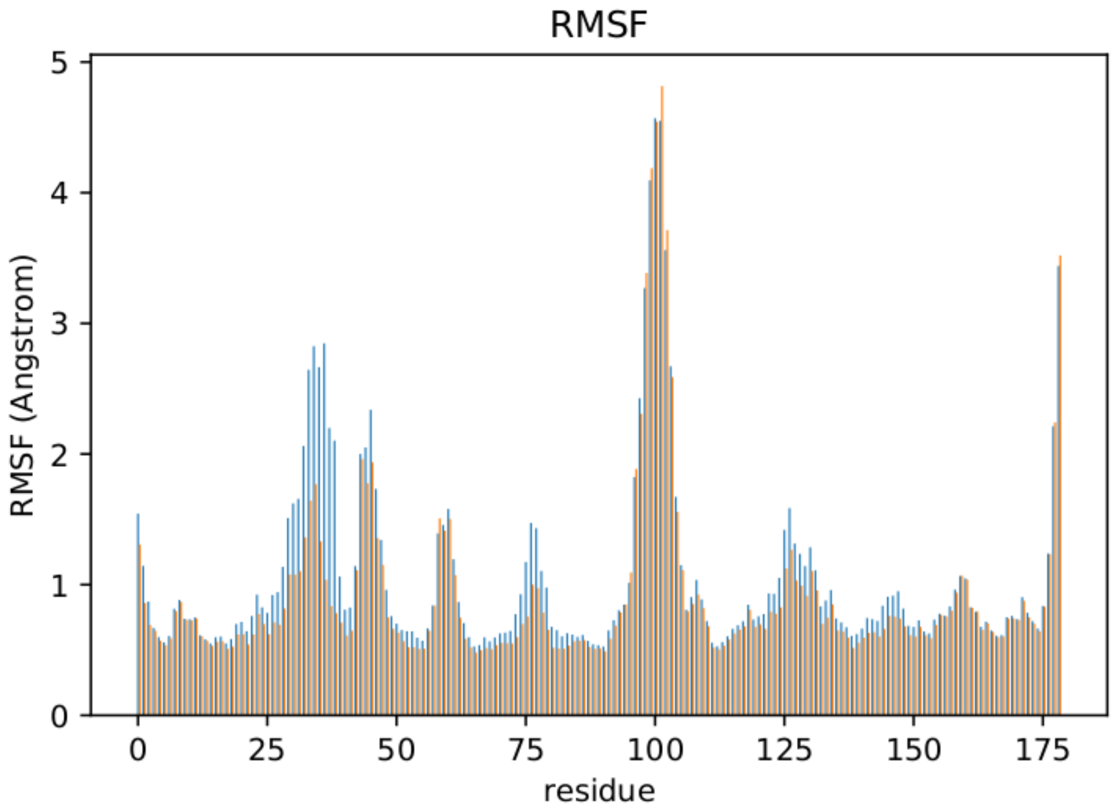

SI Figure 7: RMSF difference between apo and holo-Fe(III) from GaMD of HasAyp. Apo form is represented in blue and holo form is represented in red.

2. Conformational and explorational analysis of HasAsm GaMD simulations

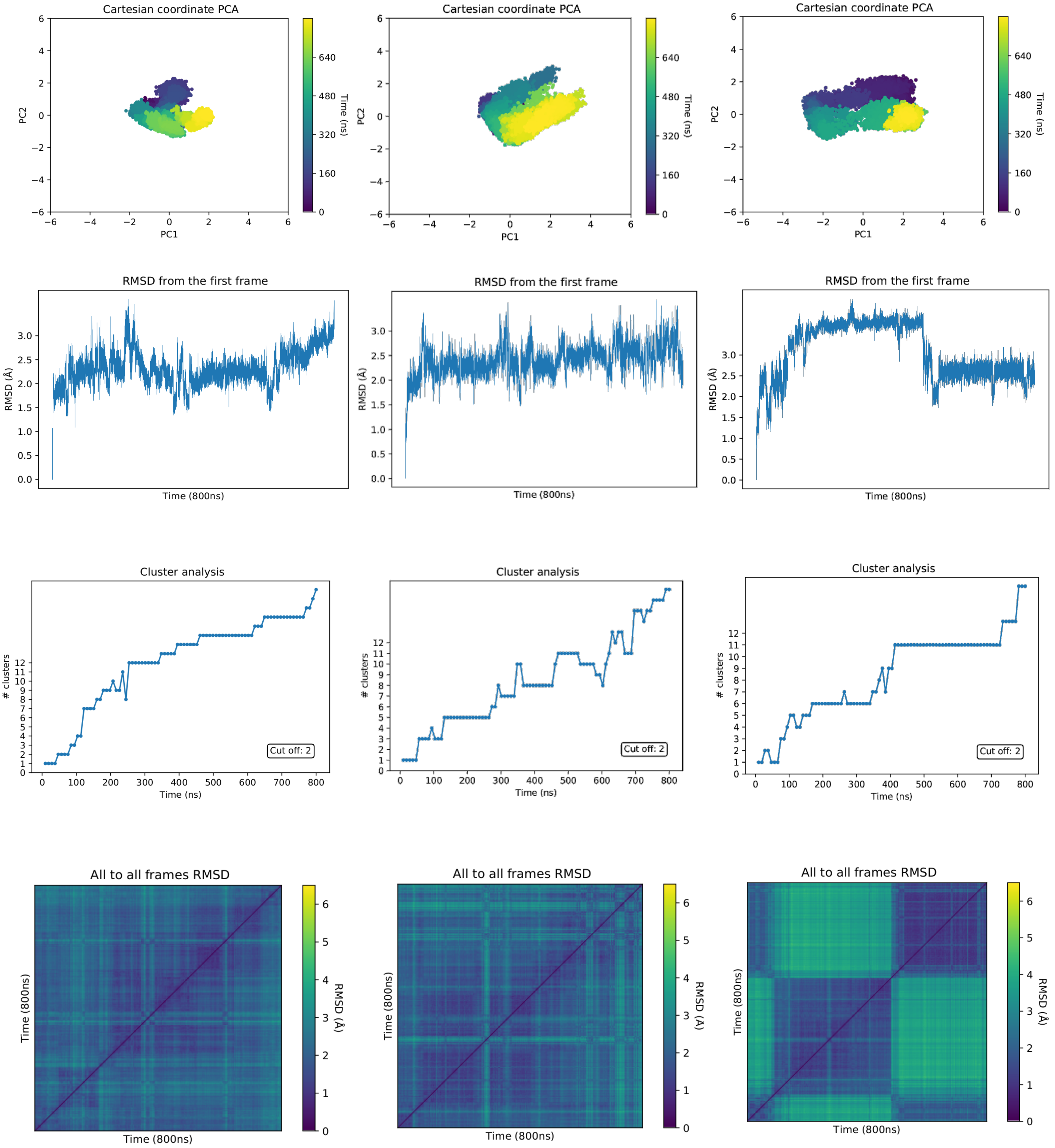

SI Figure 8: GaMD convergence analysis of HasA apo form of *Serratia marcens* (800ns – three replicas): RMSD, all-to-all RMSD, PCA andd cluster counting.

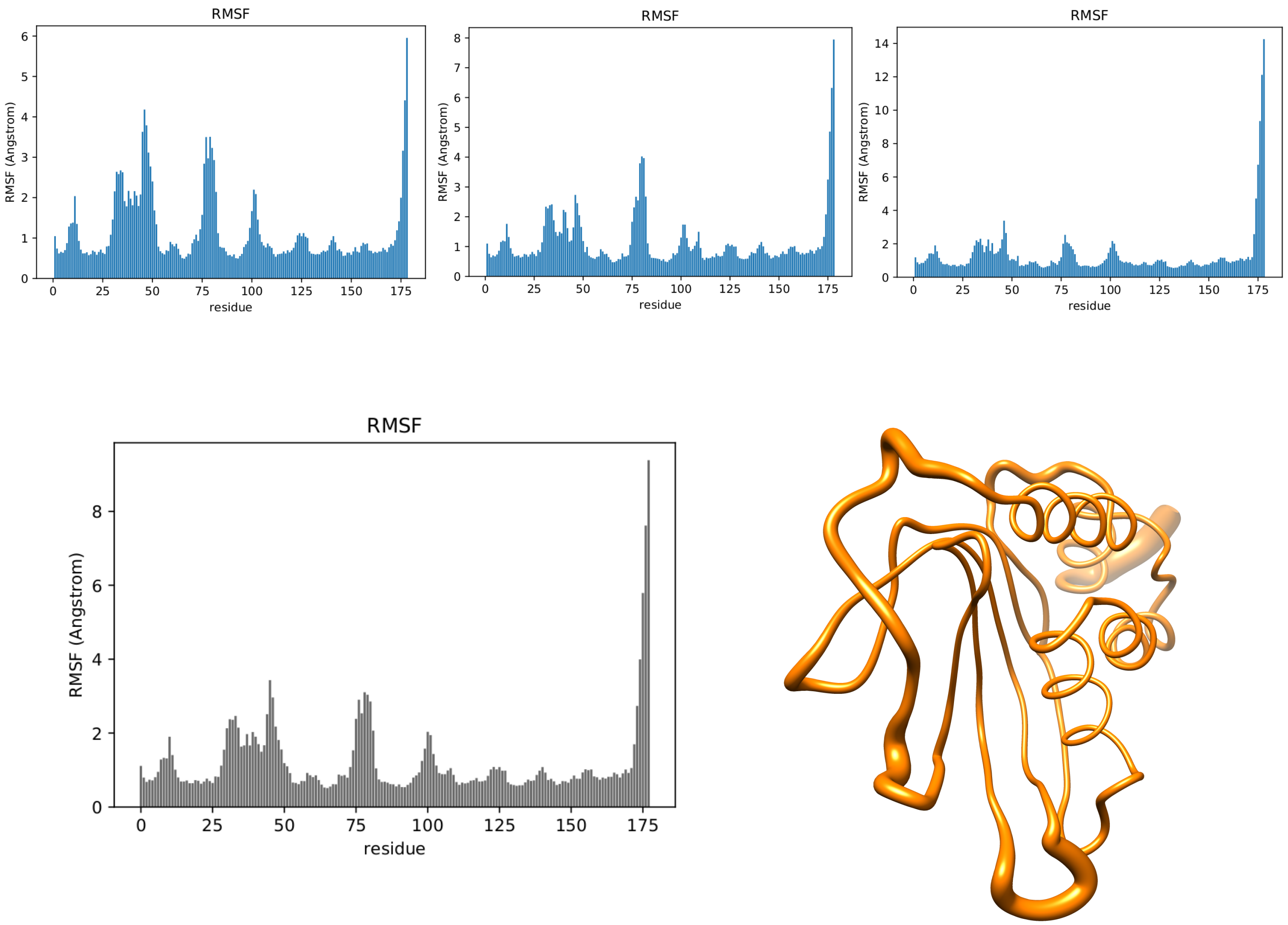

SI Figure 9: RMSF flexibility analysis of HasA apo form of *Serratia marcens* (three replicas). Average RMSF across 3 replicas and representation into structure of HasAsm apo.

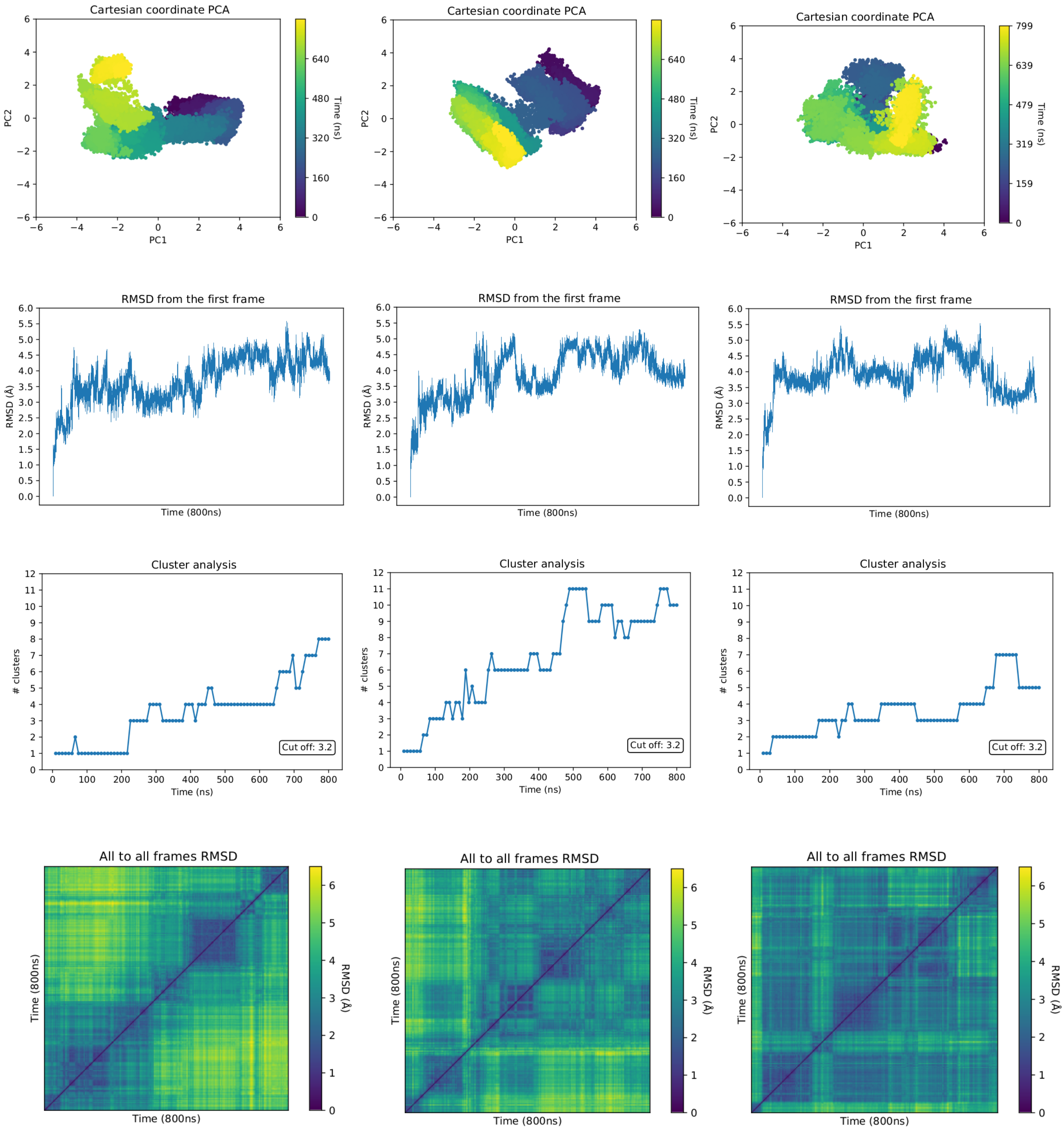

SI Figure 10: GaMD convergence analysis of HasA heme-Fe(III)-bound form of *Serratia marcens* (800ns – three replicas): RMSD, all-to-all RMSD, PCA and cluster counting.

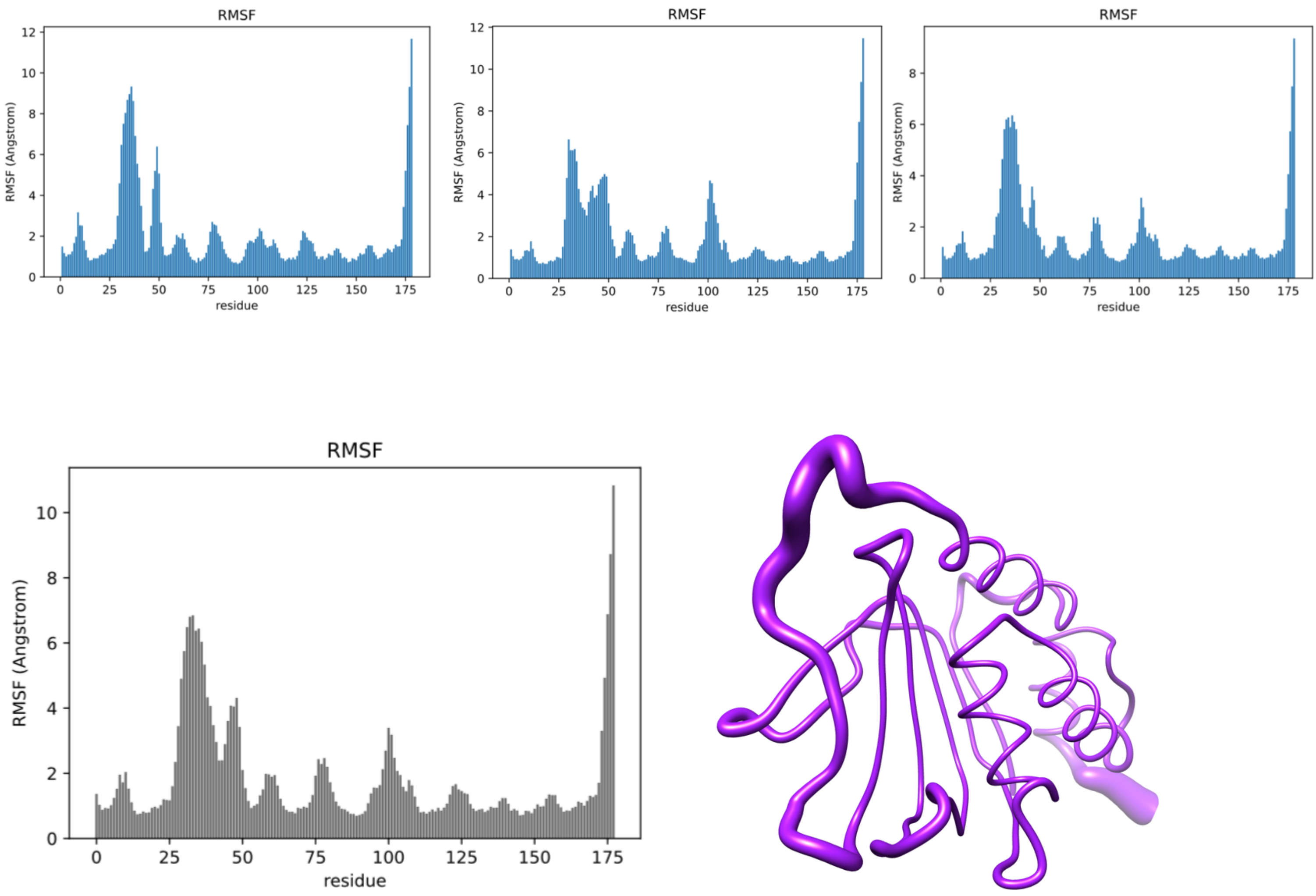

SI Figure 11: RMSF flexibility analysis of HasA heme-Fe(III)-bound form of *Serratia marcens* (three replicas). Average RMSF across 3 replicas and representation into structure of HasAsm holo.

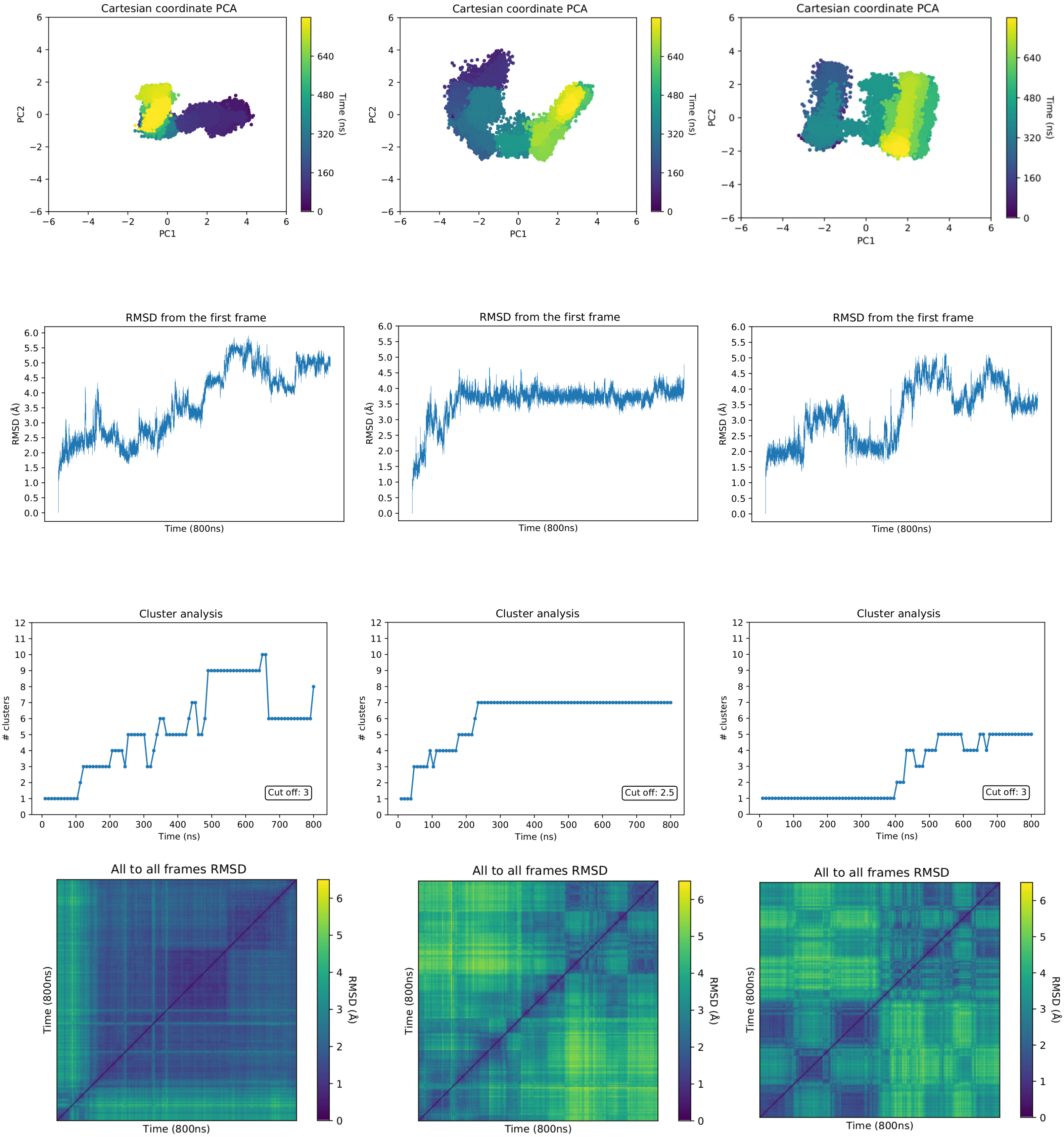

SI Figure 12: GaMD convergence analysis of HasA heme-Fe(II)-bound form of *Serratia marcens* (800ns – three replicas): RMSD, all-to-all RMSD, PCA and cluster counting.

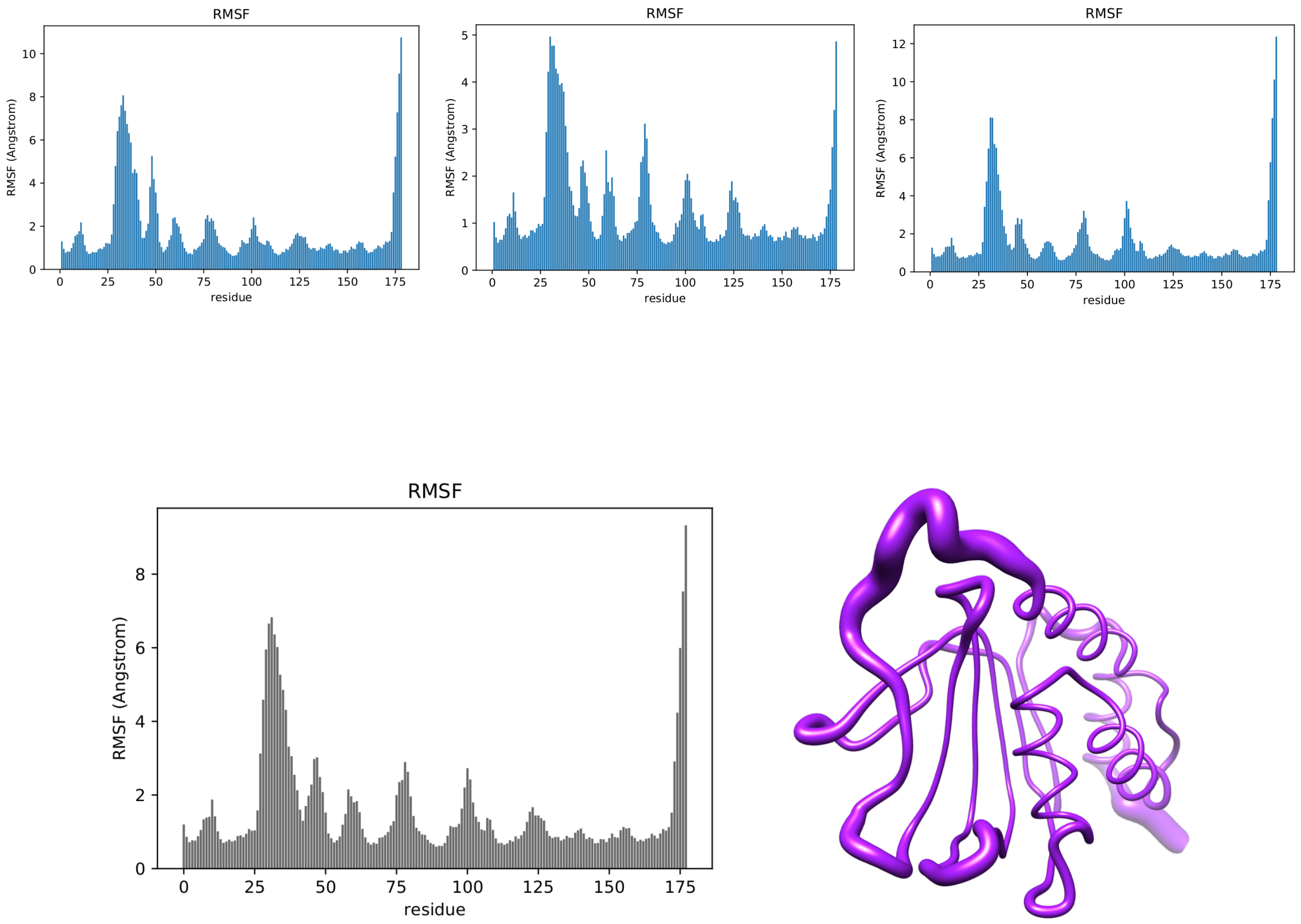

SI Figure 13: RMSF flexibility analysis of HasA heme-Fe(II)-bound form of *Serratia marcens* (three replicas). Average RMSF across 3 replicas and representation into structure of HasAsm holo.

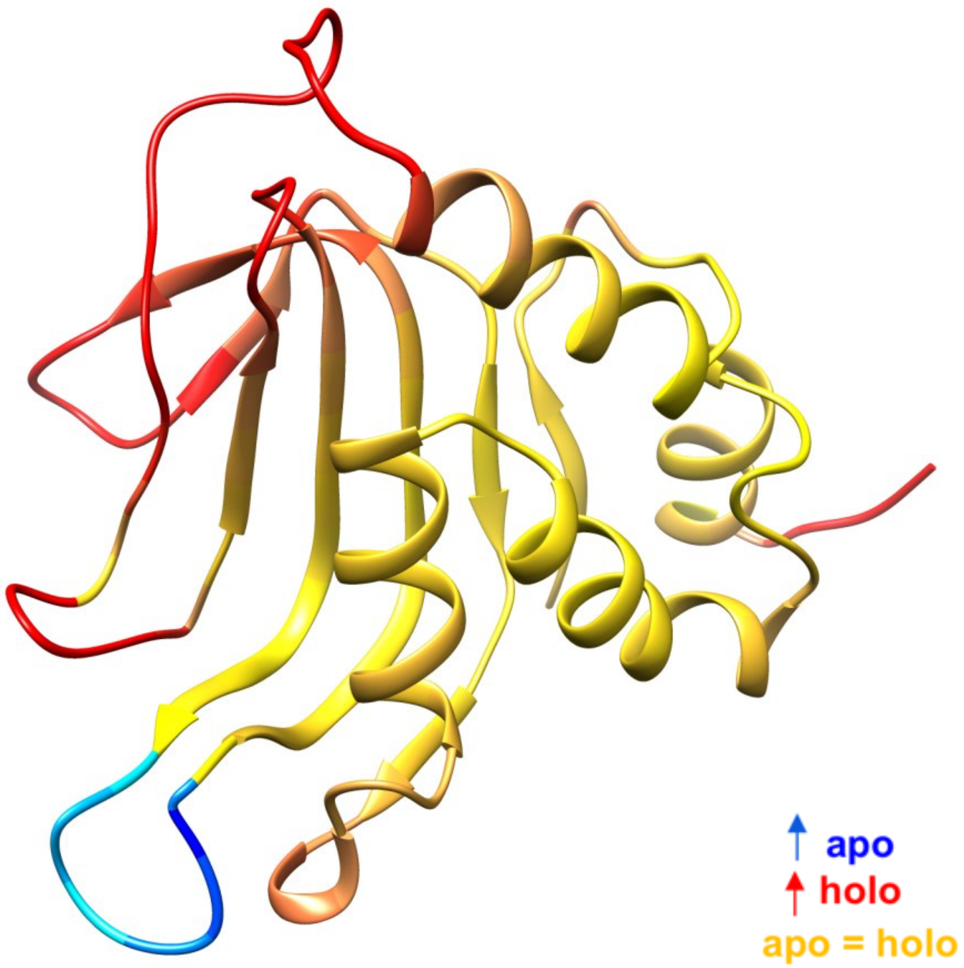

SI Figure 14: RMSF difference between apo and holo-Fe(III) from GaMD of HasAsm.

3. Interaction analysis of HasAsm GaMD simulations for Fe(II)

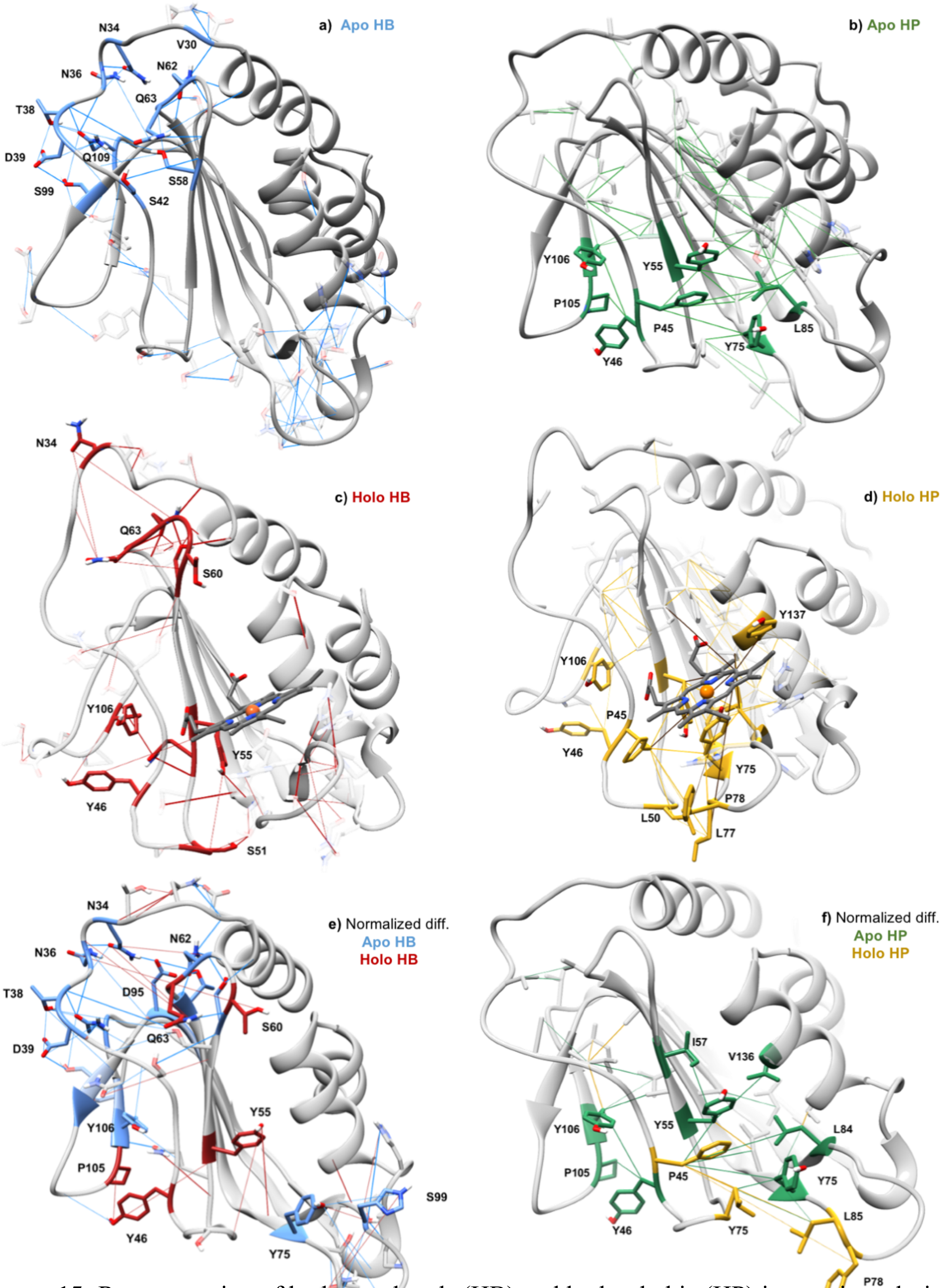

SI Figure 15: Representation of hydrogen bonds (HB) and hydrophobic (HP) interactions during GaMD of HasAsm in apo **(a)** and **(b)** and heme-Fe(II)-bound before loop L1 closing **(c)** and **(d)**. Normalized difference between apo and holo forms of both hydrogen bonds **(e)** and hydrophobic interactions **(f)** is represented.

4. GaMD analysis of holo X-ray

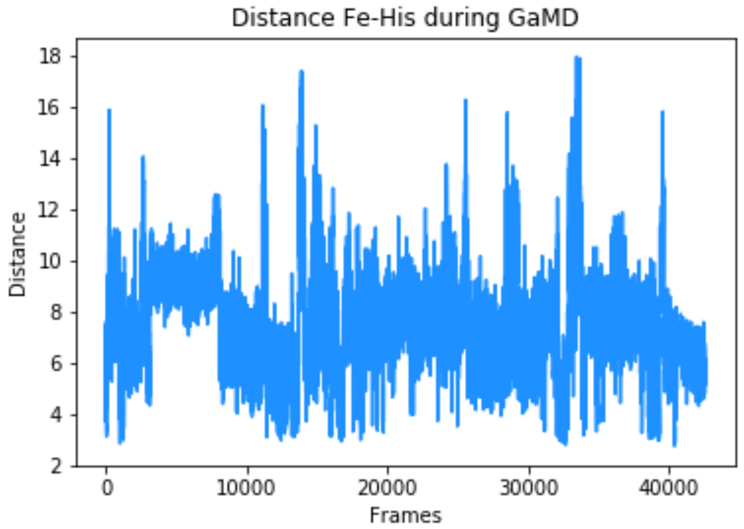

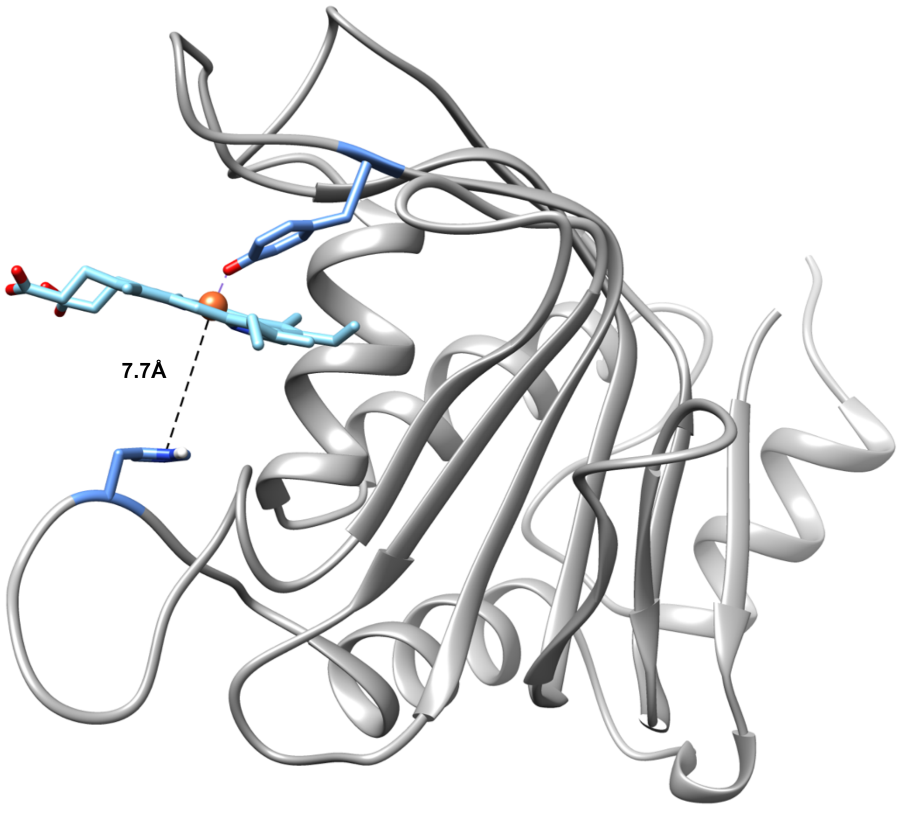

ESI Figure 16: GaMD simulation of X-ray holo (1dkh) from HasAsm with heme-Fe(III) bound.and only Tyr coordination **a)** Distance between Fe and His32 during GaMD **b)** Most representative cluster of GaMD simulation with distance between Fe and His32 displayed.

5. Analysis GaMD: Distance Fe-His, PCA and energetic analysis

<u>Results for Fe(III) replicas</u>

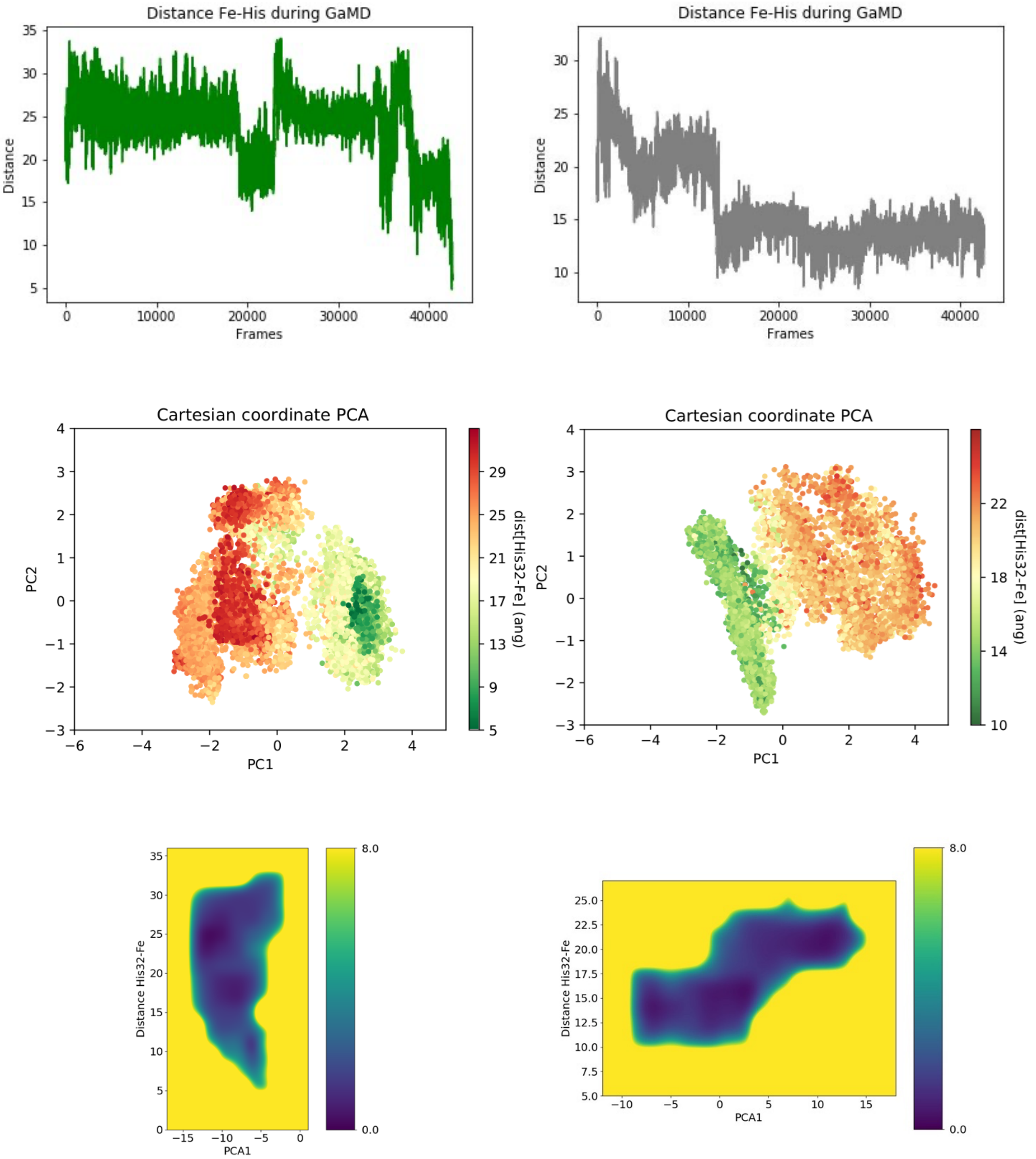

ESI Figure 17: GaMD simulation replicas of HasAsm with heme-Fe(III) bound. **a)** Distance between Fe and His32 during GaMD. **b)** Cartesian coordinate PCA analysis colored according to the distance between Fe and His32 during GaMD **c)** Reweighted PMF calculations in front of PCA1 and distance Fe-His32. b) and c) are obtained using the fragment of the trajectory in which the loop is closing

<u>Results for Fe(II) replicas</u>

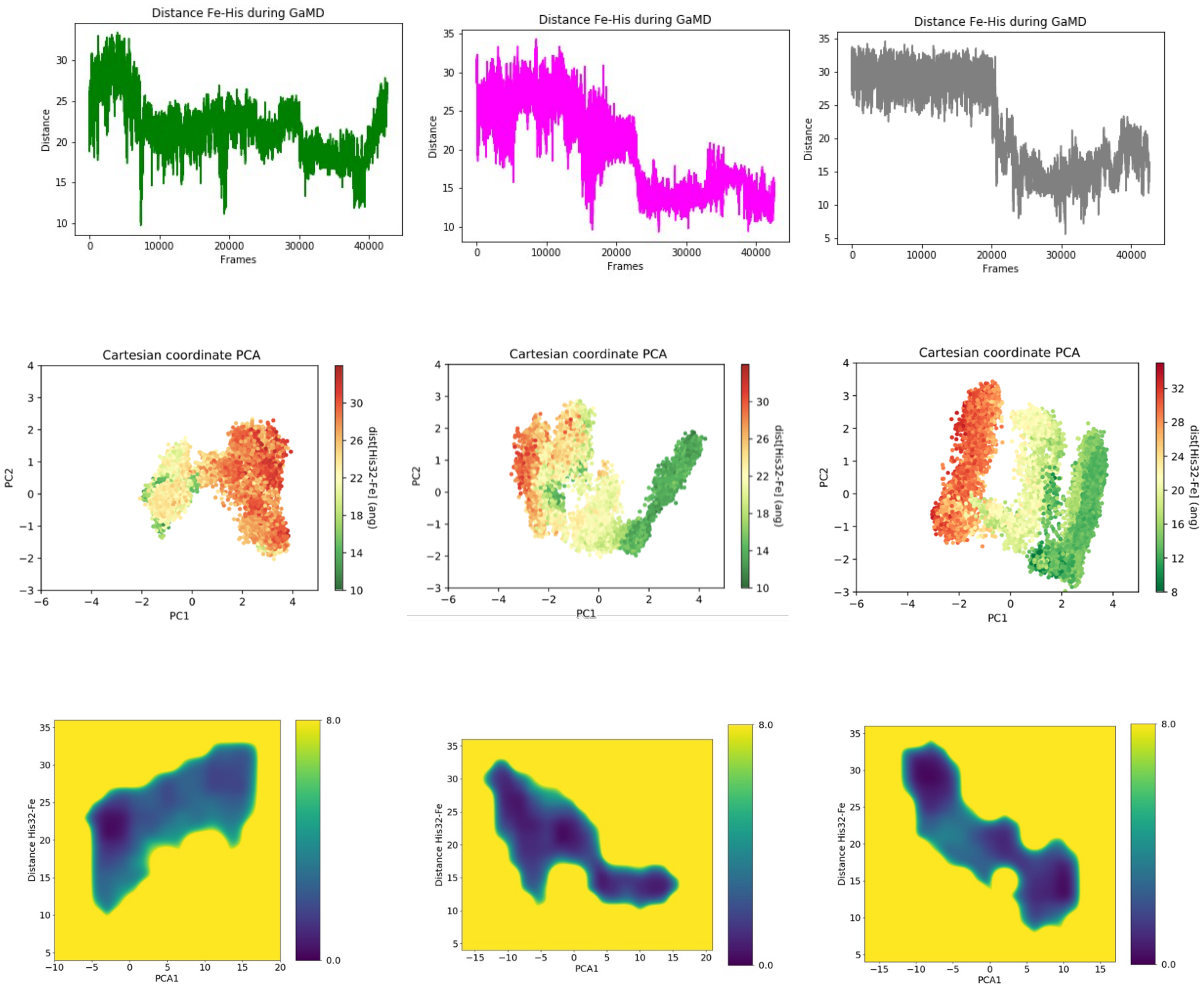

ESI Figure 18: GaMD simulation replicas of HasAsm with heme-Fe(III) bound. **a)** Distance between Fe and His32 during GaMD. **b)** Cartesian coordinate PCA analysis colored according to the distance between Fe and His32 during GaMD **c)** Reweighted PMF calculations in front of PCA1 and distance Fe-His32. b) and c) are obtained using the fragment of the trajectory in which the loop is closing

6. Force field parameters for heme

a) Parameters for Fe(II) in low spin bonded to TYR deprotonated

HM1.mol2

```
@<TRIPOS>MOLECULE
HM1
   72    76     1     0     0
SMALL
RESP Charge


@<TRIPOS>ATOM
      1 C1         19.6240   -3.7380   -6.2300 ce         1 HM1      -0.367058
      2 C2         17.0480   -2.6530   -2.2810 ce         1 HM1      -0.243416
      3 C3         13.0220   -4.2210   -4.4630 ce         1 HM1      -0.103634
      4 C4         15.6080   -5.3300   -8.4030 ce         1 HM1      -0.310608
      5 C5         19.2730   -3.3350   -4.9450 cc         1 HM1       0.312352
      6 C6         20.2280   -2.8540   -3.9520 cc         1 HM1      -0.308555
      7 C7         19.4940   -2.5400   -2.8380 cd         1 HM1       0.225608
      8 C8         18.1080   -2.8370   -3.1570 cd         1 HM1       0.079026
      9 C9         19.9750   -2.0000   -1.5240 c3         1 HM1      -0.473864
     10 C10        21.7060   -2.7570   -4.1850 c3         1 HM1       0.258293
     11 C11        22.5050   -4.0540   -3.9300 c3         1 HM1      -0.247832
     12 C12        22.1140   -5.2040   -4.9220 c          1 HM1       0.629995
     13 O1         21.0170   -5.7740   -4.7020 o          1 HM1      -0.704088
     14 O2         22.9670   -5.4580   -5.8180 o          1 HM1      -0.704088
     15 C13        15.7120   -2.9570   -2.5330 cc         1 HM1       0.055217
     16 C14        14.6370   -2.7560   -1.5860 cc         1 HM1       0.117035
     17 C15        13.4870   -3.2100   -2.2020 cd         1 HM1      -0.079801
     18 C16        13.8870   -3.6660   -3.5270 cd         1 HM1       0.052860
     19 C17        14.7860   -2.1300   -0.2330 c3         1 HM1      -0.301355
     20 C18        12.1120   -3.2280   -1.7080 cf         1 HM1      -0.048125
     21 C19        11.0290   -2.7070   -2.3090 c2         1 HM1      -0.449251
     22 C20        13.3700   -4.6800   -5.7300 cc         1 HM1      -0.141490
     23 C21        12.4350   -5.2540   -6.6730 cc         1 HM1       0.298007
     24 C22        13.1700   -5.5830   -7.7960 cd         1 HM1      -0.212941
     25 C23        14.5430   -5.1820   -7.5170 cd         1 HM1       0.243947
     26 C24        10.9610   -5.4010   -6.4500 c3         1 HM1      -0.345881
     27 C25        12.7270   -6.1670   -9.0590 cf         1 HM1      -0.004430
     28 C26        11.6750   -6.9810   -9.2510 c2         1 HM1      -0.500780
     29 C27        16.9260   -4.9620   -8.1670 cc         1 HM1       0.116759
     30 C28        18.0020   -5.1210   -9.1300 cc         1 HM1       0.214656
     31 C29        19.1440   -4.6570   -8.5280 cd         1 HM1      -0.248661
     32 C30        18.7470   -4.2290   -7.1910 cd         1 HM1       0.344629
     33 C31        17.8450   -5.6790  -10.5130 c3         1 HM1      -0.428314
     34 C32        20.5180   -4.5510   -9.1260 c3         1 HM1       0.019010
     35 C33        21.4690   -5.6870   -8.7000 c3         1 HM1       0.003276
     36 C34        21.5140   -6.9070   -9.6840 c          1 HM1       0.698197
     37 O3         22.3240   -6.7790  -10.6380 o          1 HM1      -0.742802
     38 O4         20.7480   -7.8720   -9.4270 o          1 HM1      -0.742802
     39 N1         17.9940   -3.3280   -4.4410 Y1         1 HM1      -0.482647
     40 N2         15.2400   -3.5050   -3.7060 Y2         1 HM1      -0.315850
     41 N3         14.6460   -4.6500   -6.2560 Y3         1 HM1      -0.355346
     42 N4         17.3960   -4.4130   -6.9940 Y4         1 HM1      -0.526492
     43 H1         20.6720   -3.6590   -6.5010 ha         1 HM1       0.239518
     44 H2         17.2860   -2.2460   -1.3040 ha         1 HM1       0.133119
     45 H3         11.9820   -4.3190   -4.1770 ha         1 HM1       0.108573
     46 H4         15.3930   -5.7750   -9.3680 ha         1 HM1       0.144771
     47 H5         21.8930   -2.4090   -5.2080 hc         1 HM1      -0.042703
     48 H6         22.1430   -2.0090   -3.5150 hc         1 HM1      -0.042703
     49 H7         23.5760   -3.8560   -4.0820 hc         1 HM1       0.040347
     50 H8         22.3940   -4.3950   -2.8940 hc         1 HM1       0.040347
     51 H9         11.9630   -3.7190   -0.7470 ha         1 HM1       0.101835
     52 H10        13.3230   -5.9060   -9.9330 ha         1 HM1       0.088764
     53 H11        20.4690   -4.5250  -10.2180 hc         1 HM1       0.008489
     54 H12        20.9780   -3.6020   -8.8320 hc         1 HM1       0.008489
     55 H13        22.4930   -5.3010   -8.5960 hc         1 HM1      -0.003401
     56 H14        21.1920   -6.0580   -7.7040 hc         1 HM1      -0.003401
     57 H15        10.0470   -2.7990   -1.8530 ha         1 HM1       0.130989
     58 H16        11.0820   -2.1600   -3.2450 ha         1 HM1       0.130989
```

```
     59 H17        11.0470   -7.3320   -8.4380 ha        1 HM1      0.155138
     60 H18        11.4300   -7.3420  -10.2460 ha        1 HM1      0.155138
     61 H19        19.5570   -1.0080   -1.3320 hc        1 HM1      0.105012
     62 H20        19.6820   -2.6470   -0.6880 hc        1 HM1      0.105012
     63 H21        21.0680   -1.9120   -1.4930 hc        1 HM1      0.105012
     64 H22        15.4400   -2.7240    0.4140 hc        1 HM1      0.066397
     65 H23        13.8240   -2.0280    0.2870 hc        1 HM1      0.066397
     66 H24        15.2170   -1.1280   -0.3140 hc        1 HM1      0.066397
     67 H25        10.7310   -6.2740   -5.8320 hc        1 HM1      0.078445
     68 H26        10.5550   -4.5180   -5.9460 hc        1 HM1      0.078445
     69 H27        10.4080   -5.5180   -7.3920 hc        1 HM1      0.078445
     70 H28        17.2240   -6.5790  -10.5040 hc        1 HM1      0.102003
     71 H29        18.8110   -5.9540  -10.9560 hc        1 HM1      0.102003
     72 H30        17.3740   -4.9590  -11.1920 hc        1 HM1      0.102003
@<TRIPOS>BOND
     1    1    5 1
     2    1   32 1
     3    1   43 1
     4    2    8 1
     5    2   15 1
     6    2   44 1
     7    3   18 1
     8    3   22 1
     9    3   45 1
    10    4   25 1
    11    4   29 1
    12    4   46 1
    13    5    6 1
    14    5   39 1
    15    6    7 1
    16    6   10 1
    17    7    8 1
    18    7    9 1
    19    8   39 1
    20    9   61 1
    21    9   62 1
    22    9   63 1
    23   10   11 1
    24   10   47 1
    25   10   48 1
    26   11   12 1
    27   11   49 1
    28   11   50 1
    29   12   13 1
    30   12   14 1
    31   15   16 1
    32   15   40 1
    33   16   17 1
    34   16   19 1
    35   17   18 1
    36   17   20 1
    37   18   40 1
    38   19   64 1
    39   19   65 1
    40   19   66 1
    41   20   21 1
    42   20   51 1
    43   21   57 1
    44   21   58 1
    45   22   23 1
    46   22   41 1
    47   23   24 1
    48   23   26 1
    49   24   25 1
    50   24   27 1
    51   25   41 1
    52   26   67 1
    53   26   68 1
    54   26   69 1
    55   27   28 1
    56   27   52 1
    57   28   59 1
    58   28   60 1
    59   29   30 1
    60   29   42 1
    61   30   31 1
    62   30   33 1
    63   31   32 1
    64   31   34 1
```

```
    65    32    42 1
    66    33    70 1
    67    33    71 1
    68    33    72 1
    69    34    35 1
    70    34    53 1
    71    34    54 1
    72    35    36 1
    73    35    55 1
    74    35    56 1
    75    36    37 1
    76    36    38 1
@<TRIPOS>SUBSTRUCTURE
     1 HM1         1 TEMP              0 ****  ****    0 ROOT
```

FE1.mol2

```
#Fe(II) in low spin
@<TRIPOS>MOLECULE
FE1
    1     0     1     0     0
SMALL
RESP Charge


@<TRIPOS>ATOM
      1 FE         16.3220   -3.9700   -5.3510 M1         1 FE1      0.933196
@<TRIPOS>BOND
@<TRIPOS>SUBSTRUCTURE
     1 FE1         1 TEMP              0 ****  ****    0 ROOT
```

TO1.mol2

```
@<TRIPOS>MOLECULE
TO1
20 20 1 0 0
SMALL
RESP Charge


@<TRIPOS>ATOM
      1 N1         12.7400   -8.5710    1.3560 n          1 TO11        -0.4157
      2 H1         13.2460   -8.1430    2.0950 hn         1 TO11         0.2384
      3 C1         13.2310   -8.3960   -0.0230 c3         1 TO11        -0.0014
      4 H2         12.4970   -8.7120   -0.7490 h1         1 TO11         0.0780
      5 C2         13.6480   -6.9450   -0.2350 c3         1 TO11        -0.3827
      6 H3         14.2160   -6.6040    0.6070 hc         1 TO11         0.1183
      7 H4         12.7580   -6.3520   -0.3080 hc         1 TO11         0.1183
      8 C3         14.3660   -6.7100   -1.4950 ca         1 TO11         0.1316
      9 C4         13.7310   -6.7820   -2.7200 ca         1 TO11        -0.2327
     10 H5         12.6540   -6.8850   -2.8300 ha         1 TO11         0.1291
     11 C5         14.4480   -6.5170   -3.9860 ca         1 TO11        -0.2850
     12 H6         13.8210   -6.3740   -4.8490 ha         1 TO11         0.1213
     13 C6         15.8180   -6.2650   -3.8820 ca         1 TO11         0.4124
     14 O1         16.5830   -6.0440   -4.9550 Y5         1 TO11        -0.4537
     15 C7         16.4980   -6.2870   -2.6170 ca         1 TO11        -0.2850
     16 H7         17.5350   -6.0700   -2.5260 ha         1 TO11         0.1213
     17 C8         15.7180   -6.4370   -1.4330 ca         1 TO11        -0.2327
     18 H8         16.1500   -6.2670   -0.4380 ha         1 TO11         0.1291
     19 C9         14.4090   -9.4100   -0.1740 c          1 TO11         0.5973
     20 O2         15.3260   -9.5730    0.6490 o          1 TO11        -0.5679
@<TRIPOS>BOND
     1     1     2 1
     2     1     3 1
     3     3     4 1
     4     3     5 1
     5     3    19 1
     6     5     6 1
     7     5     7 1
     8     9    10 1
     9    11    12 1
    10    13    14 1
    11    15    16 1
    12    17    18 1
    13    19    20 1
    14    11    13 1
    15    11     9 1
    16    17     8 1
```

```
    17    17    15 1
    18    13    15 1
    19     8     9 1
    20     8     5 1
@<TRIPOS>SUBSTRUCTURE
     1 TO11        1 RESIDUE           4 A     TO1     0 ROOT
```

MCPB.frcmod

```
#For heme-Fe(II) in low spin, generated by MCPB.py
MASS
M1 55.85                             Fe ion
Y1 14.01         0.530              Sp2 N in non-pure aromatic systems, identical to nc
Y2 14.01         0.530              Sp2 N in non-pure aromatic systems, identical to nc
Y3 14.01         0.530              Sp2 N in non-pure aromatic systems, identical to nc
Y4 14.01         0.530              Sp2 N in non-pure aromatic systems, identical to nc
Y5 16.00         0.434              Oxygen with one connected atom

BOND
Y1-M1   53.1    2.0256      Created by Seminario method using MCPB.py
Y2-M1   74.1    2.0298      Created by Seminario method using MCPB.py
Y3-M1   55.7    2.0297      Created by Seminario method using MCPB.py
Y4-M1   50.6    2.0257      Created by Seminario method using MCPB.py
Y5-M1   86.5    1.9738      Created by Seminario method using MCPB.py
ca-Y5  598.1    1.2358      SOURCE4_SOURCE5      17  0.0088
cc-Y1  525.4    1.3172      SOURCE3_SOURCE5    4612  0.0083
cc-Y2  525.4    1.3172      SOURCE3_SOURCE5    4612  0.0083
cc-Y3  525.4    1.3172      SOURCE3_SOURCE5    4612  0.0083
cc-Y4  525.4    1.3172      SOURCE3_SOURCE5    4612  0.0083
cd-Y1  441.1    1.3694      SOURCE1_SOURCE5    2269  0.0086
cd-Y2  441.1    1.3694      SOURCE1_SOURCE5    2269  0.0086
cd-Y3  441.1    1.3694      SOURCE1_SOURCE5    2269  0.0086
cd-Y4  441.1    1.3694      SOURCE1_SOURCE5    2269  0.0086

ANGL
Y1-M1-Y2   128.94      89.62    Created by Seminario method using MCPB.py
Y1-M1-Y3    93.27     169.19    Created by Seminario method using MCPB.py
Y1-M1-Y4   140.46      89.46    Created by Seminario method using MCPB.py
Y1-M1-Y5    77.02      88.13    Created by Seminario method using MCPB.py
Y2-M1-Y3   133.70      89.49    Created by Seminario method using MCPB.py
Y2-M1-Y4   110.39     169.63    Created by Seminario method using MCPB.py
Y2-M1-Y5    87.70      99.71    Created by Seminario method using MCPB.py
Y3-M1-Y4   141.53      89.48    Created by Seminario method using MCPB.py
Y3-M1-Y5    85.72     102.64    Created by Seminario method using MCPB.py
Y4-M1-Y5    75.83      90.58    Created by Seminario method using MCPB.py
ca-Y5-M1    88.48     136.60    Created by Seminario method using MCPB.py
cc-Y1-M1   142.35     127.17    Created by Seminario method using MCPB.py
cc-Y2-M1   175.20     126.26    Created by Seminario method using MCPB.py
cc-Y3-M1   143.02     127.18    Created by Seminario method using MCPB.py
cc-Y4-M1   133.53     126.86    Created by Seminario method using MCPB.py
cd-Y1-M1   141.32     127.05    Created by Seminario method using MCPB.py
cd-Y2-M1   176.44     127.74    Created by Seminario method using MCPB.py
cd-Y3-M1   143.12     126.94    Created by Seminario method using MCPB.py
cd-Y4-M1   135.78     127.47    Created by Seminario method using MCPB.py
Y1-cc-cc    71.6      112.56              SOURCE3          141    4.2871
Y2-cc-cc    71.6      112.56              SOURCE3          141    4.2871
Y3-cc-cc    71.6      112.56              SOURCE3          141    4.2871
Y4-cc-cc    71.6      112.56              SOURCE3          141    4.2871
ca-ca-Y5    71.4      123.26      SOURCE4_SOURCE5           35    1.2620
cc-Y1-cd    71.8      105.49         CORR_SOURCE5         1810    1.9032
cc-Y2-cd    71.8      105.49         CORR_SOURCE5         1810    1.9032
cc-Y3-cd    71.8      105.49         CORR_SOURCE5         1810    1.9032
cc-Y4-cd    71.8      105.49         CORR_SOURCE5         1810    1.9032
cd-cd-Y1    67.6      121.98         CORR_SOURCE5          141    1.9633
cd-cd-Y2    67.6      121.98         CORR_SOURCE5          141    1.9633
cd-cd-Y3    67.6      121.98         CORR_SOURCE5          141    1.9633
cd-cd-Y4    67.6      121.98         CORR_SOURCE5          141    1.9633
ce-cc-Y1    68.1      121.70         CORR_SOURCE5           58    1.4179
ce-cc-Y2    68.1      121.70         CORR_SOURCE5           58    1.4179
ce-cc-Y3    68.1      121.70         CORR_SOURCE5           58    1.4179
ce-cc-Y4    68.1      121.70         CORR_SOURCE5           58    1.4179
ce-cd-Y1    68.7      123.98      SOURCE4_SOURCE5           10    2.4097
ce-cd-Y2    68.7      123.98      SOURCE4_SOURCE5           10    2.4097
ce-cd-Y3    68.7      123.98      SOURCE4_SOURCE5           10    2.4097
ce-cd-Y4    68.7      123.98      SOURCE4_SOURCE5           10    2.4097

DIHE
X -cc-Y1-X     2         9.5      180.0   2.0      statistic value from parm94
X -cc-Y2-X     2         9.5      180.0   2.0      statistic value from parm94
```

```
X -cc-Y3-X      2        9.5      180.0   2.0      statistic value from parm94
X -cc-Y4-X      2        9.5      180.0   2.0      statistic value from parm94
X -cd-Y1-X      2        9.5      180.0   2.0      statistiv value from parm94
X -cd-Y2-X      2        9.5      180.0   2.0      statistiv value from parm94
X -cd-Y3-X      2        9.5      180.0   2.0      statistiv value from parm94
X -cd-Y4-X      2        9.5      180.0   2.0      statistiv value from parm94
M1-Y1-cc-cc     3       0.00       0.00   3.0    Treat as zero by MCPB.py
M1-Y2-cc-cc     3       0.00       0.00   3.0    Treat as zero by MCPB.py
M1-Y3-cc-cc     3       0.00       0.00   3.0    Treat as zero by MCPB.py
M1-Y4-cc-cc     3       0.00       0.00   3.0    Treat as zero by MCPB.py
Y1-M1-Y5-ca     3       0.00       0.00   3.0    Treat as zero by MCPB.py
Y2-M1-Y5-ca     3       0.00       0.00   3.0    Treat as zero by MCPB.py
Y2-cc-ce-cd     4        4.0      180.0   2.0      same as X -ce-ce-X , penalty
score=136.0
Y3-M1-Y5-ca     3       0.00       0.00   3.0    Treat as zero by MCPB.py
Y3-cc-ce-cd     4        4.0      180.0   2.0      same as X -ce-ce-X , penalty
score=136.0
Y4-M1-Y5-ca     3       0.00       0.00   3.0    Treat as zero by MCPB.py
Y4-cc-ce-cd     4        4.0      180.0   2.0      same as X -ce-ce-X , penalty
score=136.0
Y4-cd-ce-cc     4       26.6      180.0   2.0      same as X -ce-cf-X , penalty
score=136.0
ca-ca-Y5-M1     3       0.00       0.00   3.0    Treat as zero by MCPB.py
cc-Y1-M1-Y2     3       0.00       0.00   3.0    Treat as zero by MCPB.py
cc-Y1-M1-Y3     3       0.00       0.00   3.0    Treat as zero by MCPB.py
cc-Y1-M1-Y4     3       0.00       0.00   3.0    Treat as zero by MCPB.py
cc-Y1-M1-Y5     3       0.00       0.00   3.0    Treat as zero by MCPB.py
cc-Y2-M1-Y1     3       0.00       0.00   3.0    Treat as zero by MCPB.py
cc-Y2-M1-Y3     3       0.00       0.00   3.0    Treat as zero by MCPB.py
cc-Y2-M1-Y4     3       0.00       0.00   3.0    Treat as zero by MCPB.py
cc-Y2-M1-Y5     3       0.00       0.00   3.0    Treat as zero by MCPB.py
cc-Y3-M1-Y1     3       0.00       0.00   3.0    Treat as zero by MCPB.py
cc-Y3-M1-Y2     3       0.00       0.00   3.0    Treat as zero by MCPB.py
cc-Y3-M1-Y4     3       0.00       0.00   3.0    Treat as zero by MCPB.py
cc-Y3-M1-Y5     3       0.00       0.00   3.0    Treat as zero by MCPB.py
cc-Y4-M1-Y1     3       0.00       0.00   3.0    Treat as zero by MCPB.py
cc-Y4-M1-Y2     3       0.00       0.00   3.0    Treat as zero by MCPB.py
cc-Y4-M1-Y3     3       0.00       0.00   3.0    Treat as zero by MCPB.py
cc-Y4-M1-Y5     3       0.00       0.00   3.0    Treat as zero by MCPB.py
cc-ce-cd-Y1     4       26.6      180.0   2.0      same as X -ce-cf-X , penalty
score=136.0
cc-ce-cd-Y2     4       26.6      180.0   2.0      same as X -ce-cf-X , penalty
score=136.0
cc-ce-cd-Y3     4       26.6      180.0   2.0      same as X -ce-cf-X , penalty
score=136.0
cd-Y1-M1-Y2     3       0.00       0.00   3.0    Treat as zero by MCPB.py
cd-Y1-M1-Y3     3       0.00       0.00   3.0    Treat as zero by MCPB.py
cd-Y1-M1-Y4     3       0.00       0.00   3.0    Treat as zero by MCPB.py
cd-Y1-M1-Y5     3       0.00       0.00   3.0    Treat as zero by MCPB.py
cd-Y2-M1-Y1     3       0.00       0.00   3.0    Treat as zero by MCPB.py
cd-Y2-M1-Y3     3       0.00       0.00   3.0    Treat as zero by MCPB.py
cd-Y2-M1-Y4     3       0.00       0.00   3.0    Treat as zero by MCPB.py
cd-Y2-M1-Y5     3       0.00       0.00   3.0    Treat as zero by MCPB.py
cd-Y3-M1-Y1     3       0.00       0.00   3.0    Treat as zero by MCPB.py
cd-Y3-M1-Y2     3       0.00       0.00   3.0    Treat as zero by MCPB.py
cd-Y3-M1-Y4     3       0.00       0.00   3.0    Treat as zero by MCPB.py
cd-Y3-M1-Y5     3       0.00       0.00   3.0    Treat as zero by MCPB.py
cd-Y4-M1-Y1     3       0.00       0.00   3.0    Treat as zero by MCPB.py
cd-Y4-M1-Y2     3       0.00       0.00   3.0    Treat as zero by MCPB.py
cd-Y4-M1-Y3     3       0.00       0.00   3.0    Treat as zero by MCPB.py
cd-Y4-M1-Y5     3       0.00       0.00   3.0    Treat as zero by MCPB.py
cd-cd-Y1-M1     3       0.00       0.00   3.0    Treat as zero by MCPB.py
cd-cd-Y2-M1     3       0.00       0.00   3.0    Treat as zero by MCPB.py
cd-cd-Y3-M1     3       0.00       0.00   3.0    Treat as zero by MCPB.py
cd-cd-Y4-M1     3       0.00       0.00   3.0    Treat as zero by MCPB.py
cd-ce-cc-Y1     4        4.0      180.0   2.0      same as X -ce-ce-X , penalty
score=136.0
ce-cc-Y1-M1     3       0.00       0.00   3.0    Treat as zero by MCPB.py
ce-cc-Y2-M1     3       0.00       0.00   3.0    Treat as zero by MCPB.py
ce-cc-Y3-M1     3       0.00       0.00   3.0    Treat as zero by MCPB.py
ce-cc-Y4-M1     3       0.00       0.00   3.0    Treat as zero by MCPB.py
ce-cd-Y1-M1     3       0.00       0.00   3.0    Treat as zero by MCPB.py
ce-cd-Y2-M1     3       0.00       0.00   3.0    Treat as zero by MCPB.py
ce-cd-Y3-M1     3       0.00       0.00   3.0    Treat as zero by MCPB.py
ce-cd-Y4-M1     3       0.00       0.00   3.0    Treat as zero by MCPB.py
ha-ce-cc-Y1     4        4.0      180.0   2.0      same as X -ce-ce-X , penalty
score=136.0
ha-ce-cc-Y2     4        4.0      180.0   2.0      same as X -ce-ce-X , penalty
score=136.0
```

```
ha-ce-cc-Y3    4        4.0      180.0   2.0      same as X -ce-ce-X , penalty
score=136.0
ha-ce-cc-Y4    4        4.0      180.0   2.0      same as X -ce-ce-X , penalty
score=136.0
ha-ce-cd-Y1    4       16.0      180.0   2.0      same as X -cc-cd-X , penalty
score=136.0
ha-ce-cd-Y2    4       16.0      180.0   2.0      same as X -cc-cd-X , penalty
score=136.0
ha-ce-cd-Y3    4       16.0      180.0   2.0      same as X -cc-cd-X , penalty
score=136.0
ha-ce-cd-Y4    4       16.0      180.0   2.0      same as X -cc-cd-X , penalty
score=136.0

IMPR
Y3-cd-cd-ce         1.1          180.0         2.0          Using the default value
Y4-cc-cc-ce         1.1          180.0         2.0          Using the default value
Y1-cd-cd-ce         1.1          180.0         2.0          Using the default value
Y2-cd-cd-ce         1.1          180.0         2.0          Using the default value
Y5-ca-ca-ca         1.1          180.0         2.0          Using the default value
Y4-cd-cd-ce         1.1          180.0         2.0          Using the default value
Y2-cc-cc-ce         1.1          180.0         2.0          Using the default value
Y3-cc-cc-ce         1.1          180.0         2.0          Using the default value
Y1-cc-cc-ce         1.1          180.0         2.0          Using the default value

NONB
  M1          1.4090  0.0172100000       IOD set for Fe2+ ion from Li et al. JCTC, 2013,
9, 2733
  Y1          1.8240  0.1700             OPLS
  Y2          1.8240  0.1700             OPLS
  Y3          1.8240  0.1700             OPLS
  Y4          1.8240  0.1700             OPLS
  Y5          1.6612  0.2100             OPLS
```

HM1.frcmod

```
#For heme
MASS

BOND

ANGLE

DIHE
cd-cd-ce-cc   4   26.600       180.000           2.000      same as X -ce-cf-X , penalty
score=136.0
nd-cd-ce-cc   4   26.600       180.000           2.000      same as X -ce-cf-X , penalty
score=136.0
cc-cc-ce-cd   4    4.000       180.000           2.000      same as X -ce-ce-X , penalty
score=136.0
cc-cc-ce-ha   4    4.000       180.000           2.000      same as X -ce-ce-X , penalty
score=136.0
cd-cd-ce-ha   4   16.000       180.000           2.000      same as X -cc-cd-X , penalty
score=136.0
nd-cc-ce-cd   4    4.000       180.000           2.000      same as X -ce-ce-X , penalty
score=136.0
cc-cd-cf-c2   4    4.000       180.000           2.000      same as X -cf-cf-X , penalty
score=136.0
cc-cd-cf-ha   4    4.000       180.000           2.000      same as X -cf-cf-X , penalty
score=136.0
cd-cd-cf-c2   4    4.000       180.000           2.000      same as X -cf-cf-X , penalty
score=136.0
cd-cd-cf-ha   4    4.000       180.000           2.000      same as X -cf-cf-X , penalty
score=136.0
nd-cc-ce-ha   4    4.000       180.000           2.000      same as X -ce-ce-X , penalty
score=136.0
nd-cd-ce-ha   4   16.000       180.000           2.000      same as X -cc-cd-X , penalty
score=136.0

IMPROPER
cc-cd-ce-ha         1.1          180.0         2.0          Same as X -X -ca-ha, penalty
score= 46.8 (use general term))
cc-ce-cc-nd         1.1          180.0         2.0          Using the default value
c3-cc-cc-cd         1.1          180.0         2.0          Using the default value
c3-cc-cd-cd         1.1          180.0         2.0          Using the default value
cd-ce-cd-nd         1.1          180.0         2.0          Using the default value
c3-o -c -o          1.1          180.0         2.0          Using general improper
torsional angle  X- o- c- o, penalty score=  3.0)
```

```
cc-cd-cd-cf         1.1          180.0          2.0          Same as c2-ca-ca-ca, penalty
score=304.0)
c2-cd-cf-ha         1.1          180.0          2.0          Same as X -X -ca-ha, penalty
score= 46.8 (use general term))
cf-ha-c2-ha         1.1          180.0          2.0          Same as X -X -ca-ha, penalty
score= 47.1 (use general term))

NONBON
```

TO1.frcmod

```
#For tyr deprotonated
MASS

BOND
c-N    427.6    1.3789          SOURCE1_SOURCE5    9463  0.0137
C-n    427.6    1.3789          SOURCE1_SOURCE5    9463  0.0137

ANGLE
O -C -n    74.22      123.05          SOURCE3_SOURCE5      8454    1.5552
o -c -n    74.22      123.05          SOURCE3_SOURCE5      8454    1.5552
C -n -c3   63.39      120.69          SOURCE3_SOURCE5      4556    2.1510
c -N -CX   66.79      115.18          SOURCE3_SOURCE5      2997    1.3885
C3-C -n    66.79      115.18          SOURCE3_SOURCE5      2997    1.3885
c -N -H    48.33      117.55          SOURCE3_SOURCE5      5866    1.6058
C -n -hn   48.33      117.55          SOURCE3_SOURCE5      5866    1.6058
CX-C -n    66.79      115.18          SOURCE3_SOURCE5      2997    1.3885
N -c -o    74.22      123.05          SOURCE3_SOURCE5      8454    1.5552
c3-c -N    66.79      115.18          SOURCE3_SOURCE5      2997    1.3885
cc-c -n     69.1      112.70          SOURCE3_SOURCE5      1124    1.8431
c -n -c    63.74      127.08          SOURCE4_SOURCE5      1415    2.1363
C -N -c    63.74      127.08          SOURCE4_SOURCE5      1415    2.1363
n -c -n    72.90      113.56          SOURCE4_SOURCE5      1747    1.4619
n -C -N    72.90      113.56          SOURCE4_SOURCE5      1747    1.4619

DIHE
O -C -n -c3  1    2.500       180.000           2.000      same as X -c -n -X
O -C -n -hn  1    2.500       180.000           2.000      same as X -c -n -X
CX-C -n -c3  1    2.500       180.000           2.000      same as X -c -n -X
CX-C -n -hn  1    2.500       180.000           2.000      same as X -c -n -X
o -c -N -H   1    2.500       180.000           2.000      same as X -c -n -X
o -c -N -CX  1    2.500       180.000           2.000      same as X -c -n -X
c3-c -N -H   1    2.500       180.000           2.000      same as X -c -n -X
c3-c -N -CX  1    2.500       180.000           2.000      same as X -c -n -X
cc-c -N -CX  1    2.500       180.000           2.000      same as X -c -n -X
cc-c -N -H   1    2.500       180.000           2.000      same as X -c -n -X
C -N -c -c3  1    2.500       180.000           2.000      same as X -c -n -X
C -N -c -o   1    2.500       180.000           2.000      same as X -c -n -X
c3-n -C -N   1    2.500       180.000           2.000      same as X -c -n -X
hn-n -C -N   1    2.500       180.000           2.000      same as X -c -n -X

IMPROPER
ca-ca-ca-ha         1.1          180.0        2.0          Using general improper
torsional angle  X- X-ca-ha, penalty score=  6.0)
ca-ca-ca-o          1.1          180.0        2.0          Using the default value
c3-n -c -o         10.5          180.0        2.0          Using general improper
torsional angle  X- X- c- o, penalty score=  6.0)
c -hn-n -hn         1.1          180.0        2.0          Using general improper
torsional angle  X- X- n-hn, penalty score=  6.0)
c3-n -c -o         10.5          180.0        2.0          Using general improper
torsional angle  X- X- c- o, penalty score=  6.0)

NONBON
```

b) Parameters for Fe(II) in high spin bonded to TYR deprotonated

HM1.mol2

```
@<TRIPOS>MOLECULE
HM1
   72    76     1     0     0
SMALL
RESP Charge


@<TRIPOS>ATOM
      1 C1         19.6240   -3.7380   -6.2300 ce         1 HM1      -0.357370
      2 C2         17.0480   -2.6530   -2.2810 ce         1 HM1      -0.239192
      3 C3         13.0220   -4.2210   -4.4630 ce         1 HM1      -0.231550
      4 C4         15.6080   -5.3300   -8.4030 ce         1 HM1      -0.284616
      5 C5         19.2730   -3.3350   -4.9450 cc         1 HM1       0.375943
      6 C6         20.2280   -2.8540   -3.9520 cc         1 HM1      -0.240740
      7 C7         19.4940   -2.5400   -2.8380 cd         1 HM1       0.150807
      8 C8         18.1080   -2.8370   -3.1570 cd         1 HM1       0.160640
      9 C9         19.9750   -2.0000   -1.5240 c3         1 HM1      -0.432750
     10 C10        21.7060   -2.7570   -4.1850 c3         1 HM1       0.177715
     11 C11        22.5050   -4.0540   -3.9300 c3         1 HM1      -0.249514
     12 C12        22.1140   -5.2040   -4.9220 c          1 HM1       0.646793
     13 O1         21.0170   -5.7740   -4.7020 o          1 HM1      -0.675178
     14 O2         22.9670   -5.4580   -5.8180 o          1 HM1      -0.675178
     15 C13        15.7120   -2.9570   -2.5330 cc         1 HM1       0.083961
     16 C14        14.6370   -2.7560   -1.5860 cc         1 HM1       0.131978
     17 C15        13.4870   -3.2100   -2.2020 cd         1 HM1      -0.127048
     18 C16        13.8870   -3.6660   -3.5270 cd         1 HM1       0.195174
     19 C17        14.7860   -2.1300   -0.2330 c3         1 HM1      -0.308055
     20 C18        12.1120   -3.2280   -1.7080 cf         1 HM1      -0.029072
     21 C19        11.0290   -2.7070   -2.3090 c2         1 HM1      -0.433413
     22 C20        13.3700   -4.6800   -5.7300 cc         1 HM1       0.038107
     23 C21        12.4350   -5.2540   -6.6730 cc         1 HM1       0.194400
     24 C22        13.1700   -5.5830   -7.7960 cd         1 HM1      -0.163481
     25 C23        14.5430   -5.1820   -7.5170 cd         1 HM1       0.234569
     26 C24        10.9610   -5.4010   -6.4500 c3         1 HM1      -0.296958
     27 C25        12.7270   -6.1670   -9.0590 cf         1 HM1      -0.006719
     28 C26        11.6750   -6.9810   -9.2510 c2         1 HM1      -0.452733
     29 C27        16.9260   -4.9620   -8.1670 cc         1 HM1       0.181977
     30 C28        18.0020   -5.1210   -9.1300 cc         1 HM1       0.122294
     31 C29        19.1440   -4.6570   -8.5280 cd         1 HM1      -0.190372
     32 C30        18.7470   -4.2290   -7.1910 cd         1 HM1       0.353586
     33 C31        17.8450   -5.6790  -10.5130 c3         1 HM1      -0.293950
     34 C32        20.5180   -4.5510   -9.1260 c3         1 HM1      -0.046835
     35 C33        21.4690   -5.6870   -8.7000 c3         1 HM1      -0.011628
     36 C34        21.5140   -6.9070   -9.6840 c          1 HM1       0.657778
     37 O3         22.3240   -6.7790  -10.6380 o          1 HM1      -0.689237
     38 O4         20.7480   -7.8720   -9.4270 o          1 HM1      -0.689237
     39 N1         17.9940   -3.3280   -4.4410 Y2         1 HM1      -0.544520
     40 N2         15.2400   -3.5050   -3.7060 Y3         1 HM1      -0.388275
     41 N3         14.6460   -4.6500   -6.2560 Y4         1 HM1      -0.418371
     42 N4         17.3960   -4.4130   -6.9940 Y5         1 HM1      -0.512466
     43 H1         20.6720   -3.6590   -6.5010 ha         1 HM1       0.243786
     44 H2         17.2860   -2.2460   -1.3040 ha         1 HM1       0.134613
     45 H3         11.9820   -4.3190   -4.1770 ha         1 HM1       0.116049
     46 H4         15.3930   -5.7750   -9.3680 ha         1 HM1       0.131701
     47 H5         21.8930   -2.4090   -5.2080 hc         1 HM1      -0.016880
     48 H6         22.1430   -2.0090   -3.5150 hc         1 HM1      -0.016880
     49 H7         23.5760   -3.8560   -4.0820 hc         1 HM1       0.042037
     50 H8         22.3940   -4.3950   -2.8940 hc         1 HM1       0.042037
     51 H9         11.9630   -3.7190   -0.7470 ha         1 HM1       0.104846
     52 H10        13.3230   -5.9060   -9.9330 ha         1 HM1       0.101563
     53 H11        20.4690   -4.5250  -10.2180 hc         1 HM1       0.048289
     54 H12        20.9780   -3.6020   -8.8320 hc         1 HM1       0.048289
     55 H13        22.4930   -5.3010   -8.5960 hc         1 HM1       0.005377
     56 H14        21.1920   -6.0580   -7.7040 hc         1 HM1       0.005377
     57 H15        10.0470   -2.7990   -1.8530 ha         1 HM1       0.152911
     58 H16        11.0820   -2.1600   -3.2450 ha         1 HM1       0.152911
     59 H17        11.0470   -7.3320   -8.4380 ha         1 HM1       0.158855
     60 H18        11.4300   -7.3420  -10.2460 ha         1 HM1       0.158855
     61 H19        19.5570   -1.0080   -1.3320 hc         1 HM1       0.109633
     62 H20        19.6820   -2.6470   -0.6880 hc         1 HM1       0.109633
     63 H21        21.0680   -1.9120   -1.4930 hc         1 HM1       0.109633
     64 H22        15.4400   -2.7240    0.4140 hc         1 HM1       0.079233
     65 H23        13.8240   -2.0280    0.2870 hc         1 HM1       0.079233
     66 H24        15.2170   -1.1280   -0.3140 hc         1 HM1       0.079233
```

```
     67 H25        10.7310   -6.2740   -5.8320 hc         1 HM1       0.080382
     68 H26        10.5550   -4.5180   -5.9460 hc         1 HM1       0.080382
     69 H27        10.4080   -5.5180   -7.3920 hc         1 HM1       0.080382
     70 H28        17.2240   -6.5790  -10.5040 hc         1 HM1       0.080262
     71 H29        18.8110   -5.9540  -10.9560 hc         1 HM1       0.080262
     72 H30        17.3740   -4.9590  -11.1920 hc         1 HM1       0.080262
@<TRIPOS>BOND
     1    1    5 1
     2    1   32 1
     3    1   43 1
     4    2    8 1
     5    2   15 1
     6    2   44 1
     7    3   18 1
     8    3   22 1
     9    3   45 1
    10    4   25 1
    11    4   29 1
    12    4   46 1
    13    5    6 1
    14    5   39 1
    15    6    7 1
    16    6   10 1
    17    7    8 1
    18    7    9 1
    19    8   39 1
    20    9   61 1
    21    9   62 1
    22    9   63 1
    23   10   11 1
    24   10   47 1
    25   10   48 1
    26   11   12 1
    27   11   49 1
    28   11   50 1
    29   12   13 1
    30   12   14 1
    31   15   16 1
    32   15   40 1
    33   16   17 1
    34   16   19 1
    35   17   18 1
    36   17   20 1
    37   18   40 1
    38   19   64 1
    39   19   65 1
    40   19   66 1
    41   20   21 1
    42   20   51 1
    43   21   57 1
    44   21   58 1
    45   22   23 1
    46   22   41 1
    47   23   24 1
    48   23   26 1
    49   24   25 1
    50   24   27 1
    51   25   41 1
    52   26   67 1
    53   26   68 1
    54   26   69 1
    55   27   28 1
    56   27   52 1
    57   28   59 1
    58   28   60 1
    59   29   30 1
    60   29   42 1
    61   30   31 1
    62   30   33 1
    63   31   32 1
    64   31   34 1
    65   32   42 1
    66   33   70 1
    67   33   71 1
    68   33   72 1
    69   34   35 1
    70   34   53 1
    71   34   54 1
    72   35   36 1
```

```
    73   35   55 1
    74   35   56 1
    75   36   37 1
    76   36   38 1
@<TRIPOS>SUBSTRUCTURE
     1 HM1         1 TEMP              0 ****  ****    0 ROOT
```

FE1.mol2

```
#FeIII in high spin
@<TRIPOS>MOLECULE
FE1
    1     0     1     0     0
SMALL
RESP Charge


@<TRIPOS>ATOM
      1 FE         16.3220   -3.9700   -5.3510 M1         1 FE1      1.051855
@<TRIPOS>BOND
@<TRIPOS>SUBSTRUCTURE
     1 FE1         1 TEMP              0 ****  ****    0 ROOT
```

TO1.mol2

```
@<TRIPOS>MOLECULE
TO1
   20    20     1     0     0
SMALL
RESP Charge


@<TRIPOS>ATOM
      1 N1         12.7400   -8.5710    1.3560 n          1 TO1     -0.415700
      2 H1         13.2460   -8.1430    2.0950 hn         1 TO1      0.265665
      3 C1         13.2310   -8.3960   -0.0230 c3         1 TO1     -0.001400
      4 H2         12.4970   -8.7120   -0.7490 h1         1 TO1      0.042733
      5 C2         13.6480   -6.9450   -0.2350 c3         1 TO1      0.040596
      6 H3         14.2160   -6.6040    0.6070 hc         1 TO1     -0.002333
      7 H4         12.7580   -6.3520   -0.3080 hc         1 TO1     -0.002333
      8 C3         14.3660   -6.7100   -1.4950 ca         1 TO1      0.081868
      9 C4         13.7310   -6.7820   -2.7200 ca         1 TO1     -0.229735
     10 H5         12.6540   -6.8850   -2.8300 ha         1 TO1      0.149112
     11 C5         14.4480   -6.5170   -3.9860 ca         1 TO1     -0.267687
     12 H6         13.8210   -6.3740   -4.8490 ha         1 TO1      0.152435
     13 C6         15.8180   -6.2650   -3.8820 ca         1 TO1      0.406802
     14 O1         16.5830   -6.0440   -4.9550 Y1         1 TO1     -0.484933
     15 C7         16.4980   -6.2870   -2.6170 ca         1 TO1     -0.267687
     16 H7         17.5350   -6.0700   -2.5260 ha         1 TO1      0.152435
     17 C8         15.7180   -6.4370   -1.4330 ca         1 TO1     -0.229735
     18 H8         16.1500   -6.2670   -0.4380 ha         1 TO1      0.149112
     19 C9         14.4090   -9.4100   -0.1740 c          1 TO1      0.597300
     20 O2         15.3260   -9.5730    0.6490 o          1 TO1     -0.567900
@<TRIPOS>BOND
     1    1    2 1
     2    1    3 1
     3    3    4 1
     4    3    5 1
     5    3   19 1
     6    5    6 1
     7    5    7 1
     8    5    8 1
     9    8    9 1
    10    8   17 1
    11    9   10 1
    12    9   11 1
    13   11   12 1
    14   11   13 1
    15   13   14 1
    16   13   15 1
    17   15   16 1
    18   15   17 1
    29   17   18 1
    20   19   20 1
@<TRIPOS>SUBSTRUCTURE
     1 TO1         1 TEMP              0 ****  ****    0 ROOT
```

MCPB.frcmod

```
#For heme-Fe(III) in high spin, generated by MCPB.py
MASS
M1 55.85                            Fe ion
Y1 16.00          0.434             Oxygen with one connected atom
Y2 14.01          0.530             Sp2 N in non-pure aromatic systems, identical to nc
Y3 14.01          0.530             Sp2 N in non-pure aromatic systems, identical to nc
Y4 14.01          0.530             Sp2 N in non-pure aromatic systems, identical to nc
Y5 14.01          0.530             Sp2 N in non-pure aromatic systems, identical to nc

BOND
M1-Y2   47.1    2.0968      Created by Seminario method using MCPB.py
M1-Y3   42.4    2.1204      Created by Seminario method using MCPB.py
M1-Y4   46.6    2.1016      Created by Seminario method using MCPB.py
M1-Y5   51.4    2.0900      Created by Seminario method using MCPB.py
Y1-M1  117.6    1.8568      Created by Seminario method using MCPB.py
ca-Y1  598.1    1.2358      SOURCE4_SOURCE5      17  0.0088
cc-Y2  525.4    1.3172      SOURCE3_SOURCE5    4612  0.0083
cc-Y3  525.4    1.3172      SOURCE3_SOURCE5    4612  0.0083
cc-Y4  525.4    1.3172      SOURCE3_SOURCE5    4612  0.0083
cc-Y5  525.4    1.3172      SOURCE3_SOURCE5    4612  0.0083
cd-Y2  441.1    1.3694      SOURCE1_SOURCE5    2269  0.0086
cd-Y3  441.1    1.3694      SOURCE1_SOURCE5    2269  0.0086
cd-Y4  441.1    1.3694      SOURCE1_SOURCE5    2269  0.0086
cd-Y5  441.1    1.3694      SOURCE1_SOURCE5    2269  0.0086

ANGL
M1-Y2-cc    61.36     126.04    Created by Seminario method using MCPB.py
M1-Y2-cd    68.99     126.22    Created by Seminario method using MCPB.py
M1-Y3-cc    64.43     125.12    Created by Seminario method using MCPB.py
M1-Y3-cd    50.20     126.69    Created by Seminario method using MCPB.py
M1-Y4-cc    89.73     126.13    Created by Seminario method using MCPB.py
M1-Y4-cd    90.30     125.60    Created by Seminario method using MCPB.py
M1-Y5-cc    79.46     125.56    Created by Seminario method using MCPB.py
M1-Y5-cd    70.56     126.26    Created by Seminario method using MCPB.py
Y1-M1-Y2    23.60     100.19    Created by Seminario method using MCPB.py
Y1-M1-Y3    28.10     102.16    Created by Seminario method using MCPB.py
Y1-M1-Y4    27.28     106.50    Created by Seminario method using MCPB.py
Y1-M1-Y5    29.19     103.83    Created by Seminario method using MCPB.py
Y3-M1-Y2    47.15      86.88    Created by Seminario method using MCPB.py
Y4-M1-Y2    43.11     153.30    Created by Seminario method using MCPB.py
Y4-M1-Y3    33.04      86.69    Created by Seminario method using MCPB.py
Y5-M1-Y2    41.94      87.11    Created by Seminario method using MCPB.py
Y5-M1-Y3    46.09     153.97    Created by Seminario method using MCPB.py
Y5-M1-Y4    45.53      87.40    Created by Seminario method using MCPB.py
ca-Y1-M1    34.88     152.79    Created by Seminario method using MCPB.py
Y2-cc-cc    71.6      112.56              SOURCE3          141    4.2871
Y3-cc-cc    71.6      112.56              SOURCE3          141    4.2871
Y4-cc-cc    71.6      112.56              SOURCE3          141    4.2871
Y5-cc-cc    71.6      112.56              SOURCE3          141    4.2871
ca-ca-Y1    71.4      123.26      SOURCE4_SOURCE5           35    1.2620
cc-Y2-cd    71.8      105.49         CORR_SOURCE5         1810    1.9032
cc-Y3-cd    71.8      105.49         CORR_SOURCE5         1810    1.9032
cc-Y4-cd    71.8      105.49         CORR_SOURCE5         1810    1.9032
cc-Y5-cd    71.8      105.49         CORR_SOURCE5         1810    1.9032
cd-cd-Y2    67.6      121.98         CORR_SOURCE5          141    1.9633
cd-cd-Y3    67.6      121.98         CORR_SOURCE5          141    1.9633
cd-cd-Y4    67.6      121.98         CORR_SOURCE5          141    1.9633
cd-cd-Y5    67.6      121.98         CORR_SOURCE5          141    1.9633
ce-cc-Y2    68.1      121.70         CORR_SOURCE5           58    1.4179
ce-cc-Y3    68.1      121.70         CORR_SOURCE5           58    1.4179
ce-cc-Y4    68.1      121.70         CORR_SOURCE5           58    1.4179
ce-cc-Y5    68.1      121.70         CORR_SOURCE5           58    1.4179
ce-cd-Y2    68.7      123.98      SOURCE4_SOURCE5           10    2.4097
ce-cd-Y3    68.7      123.98      SOURCE4_SOURCE5           10    2.4097
ce-cd-Y4    68.7      123.98      SOURCE4_SOURCE5           10    2.4097
ce-cd-Y5    68.7      123.98      SOURCE4_SOURCE5           10    2.4097

DIHE
X -cc-Y2-X     2        9.5      180.0   2.0      statistic value from parm94
X -cc-Y3-X     2        9.5      180.0   2.0      statistic value from parm94
X -cc-Y4-X     2        9.5      180.0   2.0      statistic value from parm94
X -cc-Y5-X     2        9.5      180.0   2.0      statistic value from parm94
X -cd-Y2-X     2        9.5      180.0   2.0      statistiv value from parm94
X -cd-Y3-X     2        9.5      180.0   2.0      statistiv value from parm94
X -cd-Y4-X     2        9.5      180.0   2.0      statistiv value from parm94
X -cd-Y5-X     2        9.5      180.0   2.0      statistiv value from parm94
M1-Y2-cc-cc    3       0.00       0.00   3.0    Treat as zero by MCPB.py
```

M1-Y2-cc-ce	3	0.00	0.00	3.0	Treat as zero by MCPB.py
M1-Y2-cd-cd	3	0.00	0.00	3.0	Treat as zero by MCPB.py
M1-Y2-cd-ce	3	0.00	0.00	3.0	Treat as zero by MCPB.py
M1-Y3-cc-cc	3	0.00	0.00	3.0	Treat as zero by MCPB.py
M1-Y3-cc-ce	3	0.00	0.00	3.0	Treat as zero by MCPB.py
M1-Y3-cd-cd	3	0.00	0.00	3.0	Treat as zero by MCPB.py
M1-Y3-cd-ce	3	0.00	0.00	3.0	Treat as zero by MCPB.py
M1-Y4-cc-cc	3	0.00	0.00	3.0	Treat as zero by MCPB.py
M1-Y4-cc-ce	3	0.00	0.00	3.0	Treat as zero by MCPB.py
M1-Y4-cd-cd	3	0.00	0.00	3.0	Treat as zero by MCPB.py
M1-Y4-cd-ce	3	0.00	0.00	3.0	Treat as zero by MCPB.py
M1-Y5-cc-cc	3	0.00	0.00	3.0	Treat as zero by MCPB.py
M1-Y5-cc-ce	3	0.00	0.00	3.0	Treat as zero by MCPB.py
M1-Y5-cd-cd	3	0.00	0.00	3.0	Treat as zero by MCPB.py
M1-Y5-cd-ce	3	0.00	0.00	3.0	Treat as zero by MCPB.py
Y1-M1-Y2-cc	3	0.00	0.00	3.0	Treat as zero by MCPB.py
Y1-M1-Y2-cd	3	0.00	0.00	3.0	Treat as zero by MCPB.py
Y1-M1-Y3-cc	3	0.00	0.00	3.0	Treat as zero by MCPB.py
Y1-M1-Y3-cd	3	0.00	0.00	3.0	Treat as zero by MCPB.py
Y1-M1-Y4-cc	3	0.00	0.00	3.0	Treat as zero by MCPB.py
Y1-M1-Y4-cd	3	0.00	0.00	3.0	Treat as zero by MCPB.py
Y1-M1-Y5-cc	3	0.00	0.00	3.0	Treat as zero by MCPB.py
Y1-M1-Y5-cd	3	0.00	0.00	3.0	Treat as zero by MCPB.py
Y3-M1-Y2-cc	3	0.00	0.00	3.0	Treat as zero by MCPB.py
Y3-M1-Y2-cd	3	0.00	0.00	3.0	Treat as zero by MCPB.py
Y3-cc-ce-cd	4	4.0	180.0	2.0	same as X -ce-ce-X , penalty score=136.0
Y4-M1-Y2-cc	3	0.00	0.00	3.0	Treat as zero by MCPB.py
Y4-M1-Y2-cd	3	0.00	0.00	3.0	Treat as zero by MCPB.py
Y4-M1-Y3-cc	3	0.00	0.00	3.0	Treat as zero by MCPB.py
Y4-M1-Y3-cd	3	0.00	0.00	3.0	Treat as zero by MCPB.py
Y4-cc-ce-cd	4	4.0	180.0	2.0	same as X -ce-ce-X , penalty score=136.0
Y5-M1-Y2-cc	3	0.00	0.00	3.0	Treat as zero by MCPB.py
Y5-M1-Y2-cd	3	0.00	0.00	3.0	Treat as zero by MCPB.py
Y5-M1-Y3-cc	3	0.00	0.00	3.0	Treat as zero by MCPB.py
Y5-M1-Y3-cd	3	0.00	0.00	3.0	Treat as zero by MCPB.py
Y5-M1-Y4-cc	3	0.00	0.00	3.0	Treat as zero by MCPB.py
Y5-M1-Y4-cd	3	0.00	0.00	3.0	Treat as zero by MCPB.py
Y5-cc-ce-cd	4	4.0	180.0	2.0	same as X -ce-ce-X , penalty score=136.0
Y5-cd-ce-cc	4	26.6	180.0	2.0	same as X -ce-cf-X , penalty score=136.0
ca-Y1-M1-Y2	3	0.00	0.00	3.0	Treat as zero by MCPB.py
ca-Y1-M1-Y3	3	0.00	0.00	3.0	Treat as zero by MCPB.py
ca-Y1-M1-Y4	3	0.00	0.00	3.0	Treat as zero by MCPB.py
ca-Y1-M1-Y5	3	0.00	0.00	3.0	Treat as zero by MCPB.py
ca-ca-Y1-M1	3	0.00	0.00	3.0	Treat as zero by MCPB.py
cc-Y3-M1-Y2	3	0.00	0.00	3.0	Treat as zero by MCPB.py
cc-Y4-M1-Y2	3	0.00	0.00	3.0	Treat as zero by MCPB.py
cc-Y4-M1-Y3	3	0.00	0.00	3.0	Treat as zero by MCPB.py
cc-Y5-M1-Y2	3	0.00	0.00	3.0	Treat as zero by MCPB.py
cc-Y5-M1-Y3	3	0.00	0.00	3.0	Treat as zero by MCPB.py
cc-Y5-M1-Y4	3	0.00	0.00	3.0	Treat as zero by MCPB.py
cc-ce-cd-Y2	4	26.6	180.0	2.0	same as X -ce-cf-X , penalty score=136.0
cc-ce-cd-Y3	4	26.6	180.0	2.0	same as X -ce-cf-X , penalty score=136.0
cc-ce-cd-Y4	4	26.6	180.0	2.0	same as X -ce-cf-X , penalty score=136.0
cd-Y3-M1-Y2	3	0.00	0.00	3.0	Treat as zero by MCPB.py
cd-Y4-M1-Y2	3	0.00	0.00	3.0	Treat as zero by MCPB.py
cd-Y4-M1-Y3	3	0.00	0.00	3.0	Treat as zero by MCPB.py
cd-Y5-M1-Y2	3	0.00	0.00	3.0	Treat as zero by MCPB.py
cd-Y5-M1-Y3	3	0.00	0.00	3.0	Treat as zero by MCPB.py
cd-Y5-M1-Y4	3	0.00	0.00	3.0	Treat as zero by MCPB.py
cd-ce-cc-Y2	4	4.0	180.0	2.0	same as X -ce-ce-X , penalty score=136.0
ha-ce-cc-Y2	4	4.0	180.0	2.0	same as X -ce-ce-X , penalty score=136.0
ha-ce-cc-Y3	4	4.0	180.0	2.0	same as X -ce-ce-X , penalty score=136.0
ha-ce-cc-Y4	4	4.0	180.0	2.0	same as X -ce-ce-X , penalty score=136.0
ha-ce-cc-Y5	4	4.0	180.0	2.0	same as X -ce-ce-X , penalty score=136.0
ha-ce-cd-Y2	4	16.0	180.0	2.0	same as X -cc-cd-X , penalty score=136.0

```
ha-ce-cd-Y3    4       16.0      180.0   2.0      same as X -cc-cd-X , penalty
score=136.0
ha-ce-cd-Y4    4       16.0      180.0   2.0      same as X -cc-cd-X , penalty
score=136.0
ha-ce-cd-Y5    4       16.0      180.0   2.0      same as X -cc-cd-X , penalty
score=136.0

IMPR
Y1-ca-ca-ca         1.1          180.0         2.0          Using the default value
Y2-cc-cc-ce         1.1          180.0         2.0          Using the default value
Y5-cd-cd-ce         1.1          180.0         2.0          Using the default value
Y2-cd-cd-ce         1.1          180.0         2.0          Using the default value
Y3-cc-cc-ce         1.1          180.0         2.0          Using the default value
Y3-cd-cd-ce         1.1          180.0         2.0          Using the default value
Y4-cc-cc-ce         1.1          180.0         2.0          Using the default value
Y4-cd-cd-ce         1.1          180.0         2.0          Using the default value
Y5-cc-cc-ce         1.1          180.0         2.0          Using the default value

NONB
  M1          1.4000  0.0157074900       IOD set for Fe3+ ion OPC water from Li et al.
JCTC, 2021, 17, 2342
  Y1          1.6612  0.2100             OPLS
  Y2          1.8240  0.1700             OPLS
  Y3          1.8240  0.1700             OPLS
  Y4          1.8240  0.1700             OPLS
  Y5          1.8240  0.1700             OPLS
```

HM1.frcmod

```
#For heme
MASS

BOND

ANGLE

DIHE
cd-cd-ce-cc   4   26.600       180.000           2.000      same as X -ce-cf-X , penalty
score=136.0
nd-cd-ce-cc   4   26.600       180.000           2.000      same as X -ce-cf-X , penalty
score=136.0
cc-cc-ce-cd   4    4.000       180.000           2.000      same as X -ce-ce-X , penalty
score=136.0
cc-cc-ce-ha   4    4.000       180.000           2.000      same as X -ce-ce-X , penalty
score=136.0
cd-cd-ce-ha   4   16.000       180.000           2.000      same as X -cc-cd-X , penalty
score=136.0
nd-cc-ce-cd   4    4.000       180.000           2.000      same as X -ce-ce-X , penalty
score=136.0
cc-cd-cf-c2   4    4.000       180.000           2.000      same as X -cf-cf-X , penalty
score=136.0
cc-cd-cf-ha   4    4.000       180.000           2.000      same as X -cf-cf-X , penalty
score=136.0
cd-cd-cf-c2   4    4.000       180.000           2.000      same as X -cf-cf-X , penalty
score=136.0
cd-cd-cf-ha   4    4.000       180.000           2.000      same as X -cf-cf-X , penalty
score=136.0
nd-cc-ce-ha   4    4.000       180.000           2.000      same as X -ce-ce-X , penalty
score=136.0
nd-cd-ce-ha   4   16.000       180.000           2.000      same as X -cc-cd-X , penalty
score=136.0

IMPROPER
cc-cd-ce-ha         1.1          180.0         2.0          Same as X -X -ca-ha, penalty
score= 46.8 (use general term))
cc-ce-cc-nd         1.1          180.0         2.0          Using the default value
c3-cc-cc-cd         1.1          180.0         2.0          Using the default value
c3-cc-cd-cd         1.1          180.0         2.0          Using the default value
cd-ce-cd-nd         1.1          180.0         2.0          Using the default value
c3-o -c -o          1.1          180.0         2.0          Using general improper
torsional angle  X- o- c- o, penalty score=  3.0)
cc-cd-cd-cf         1.1          180.0         2.0          Same as c2-ca-ca-ca, penalty
score=304.0)
c2-cd-cf-ha         1.1          180.0         2.0          Same as X -X -ca-ha, penalty
score= 46.8 (use general term))
cf-ha-c2-ha         1.1          180.0         2.0          Same as X -X -ca-ha, penalty
score= 47.1 (use general term))

NONBON
```

<u>TO1.frcmod</u>

```
#For TYR deprotonated
MASS

BOND
c-N    427.6    1.3789          SOURCE1_SOURCE5    9463  0.0137
C-n    427.6    1.3789          SOURCE1_SOURCE5    9463  0.0137

ANGLE
O -C -n    74.22      123.05          SOURCE3_SOURCE5      8454    1.5552
o -c -n    74.22      123.05          SOURCE3_SOURCE5      8454    1.5552
C -n -c3   63.39      120.69          SOURCE3_SOURCE5      4556    2.1510
c -N -CX   66.79      115.18          SOURCE3_SOURCE5      2997    1.3885
C3-C -n    66.79      115.18          SOURCE3_SOURCE5      2997    1.3885
c -N -H    48.33      117.55          SOURCE3_SOURCE5      5866    1.6058
C -n -hn   48.33      117.55          SOURCE3_SOURCE5      5866    1.6058
CX-C -n    66.79      115.18          SOURCE3_SOURCE5      2997    1.3885
N -c -o    74.22      123.05          SOURCE3_SOURCE5      8454    1.5552
c3-c -N    66.79      115.18          SOURCE3_SOURCE5      2997    1.3885
cc-c -n     69.1      112.70      SOURCE3_SOURCE5         1124    1.8431
c -n -c    63.74      127.08          SOURCE4_SOURCE5      1415    2.1363
C -N -c    63.74      127.08          SOURCE4_SOURCE5      1415    2.1363
n -c -n    72.90      113.56          SOURCE4_SOURCE5      1747    1.4619
n -C -N    72.90      113.56          SOURCE4_SOURCE5      1747    1.4619

DIHE
O -C -n -c3  1    2.500       180.000           2.000      same as X -c -n -X
O -C -n -hn  1    2.500       180.000           2.000      same as X -c -n -X
CX-C -n -c3  1    2.500       180.000           2.000      same as X -c -n -X
CX-C -n -hn  1    2.500       180.000           2.000      same as X -c -n -X
o -c -N -H   1    2.500       180.000           2.000      same as X -c -n -X
o -c -N -CX  1    2.500       180.000           2.000      same as X -c -n -X
c3-c -N -H   1    2.500       180.000           2.000      same as X -c -n -X
c3-c -N -CX  1    2.500       180.000           2.000      same as X -c -n -X
cc-c -N -CX  1    2.500       180.000           2.000      same as X -c -n -X
cc-c -N -H   1    2.500       180.000           2.000      same as X -c -n -X
C -N -c -c3  1    2.500       180.000           2.000      same as X -c -n -X
C -N -c -o   1    2.500       180.000           2.000      same as X -c -n -X
c3-n -C -N   1    2.500       180.000           2.000      same as X -c -n -X
hn-n -C -N   1    2.500       180.000           2.000      same as X -c -n -X

IMPROPER
ca-ca-ca-ha         1.1          180.0         2.0          Using general improper
torsional angle  X- X-ca-ha, penalty score=  6.0)
ca-ca-ca-o          1.1          180.0         2.0          Using the default value
c3-n -c -o         10.5          180.0         2.0          Using general improper
torsional angle  X- X- c- o, penalty score=  6.0)
c -hn-n -hn         1.1          180.0         2.0          Using general improper
torsional angle  X- X- n-hn, penalty score=  6.0)
c3-n -c -o         10.5          180.0         2.0          Using general improper
torsional angle  X- X- c- o, penalty score=  6.0)

NONBON
```

c) Parameters for Fe(III) in low spin bonded to HIS and TYR deprotonated

HM1.mol2

```
@<TRIPOS>MOLECULE
HM1
   72    76     1     0     0
SMALL
RESP Charge


@<TRIPOS>ATOM
      1 CHA        18.1470    1.5370   -3.5330 ce        1 HM1     -0.261767
      2 CHB        13.7730   -0.2540   -2.8320 ce        1 HM1     -0.059493
      3 CHC        13.7940    1.2740    1.7120 ce        1 HM1     -0.078405
      4 CHD        18.2350    3.0830    1.0900 ce        1 HM1     -0.175064
      5 C1A        16.9580    0.7380   -3.7720 cc        1 HM1      0.125382
      6 C2A        16.6350    0.0130   -4.9150 cc        1 HM1     -0.237065
      7 C3A        15.3670   -0.5120   -4.7720 cd        1 HM1      0.259926
      8 C4A        14.9930   -0.0210   -3.4660 cd        1 HM1     -0.155727
      9 CMA        14.5920   -1.4740   -5.6650 c3        1 HM1     -0.446828
     10 CAA        17.4720   -0.2900   -6.1450 c3        1 HM1      0.030467
     11 CBA        17.8160    1.0110   -7.0190 c3        1 HM1     -0.165013
     12 CGA        18.2700    0.7410   -8.4010 c         1 HM1      0.697749
     13 O1A        19.1810   -0.0310   -8.5890 o         1 HM1     -0.712101
     14 O2A        17.6740    1.3090   -9.3310 o         1 HM1     -0.712101
     15 C1B        13.2620    0.0740   -1.5030 cc        1 HM1     -0.151546
     16 C2B        12.1580   -0.5060   -0.7870 cc        1 HM1      0.166437
     17 C3B        12.2430   -0.1830    0.5340 cd        1 HM1     -0.050842
     18 C4B        13.3270    0.7360    0.5460 cd        1 HM1     -0.048760
     19 CMB        11.2730   -1.5980   -1.4750 c3        1 HM1     -0.309989
     20 CAB        11.4210   -0.6810    1.5610 cf        1 HM1     -0.061036
     21 CBB        11.7360   -0.8990    2.8130 c2        1 HM1     -0.416189
     22 C1C        15.0360    1.9550    1.9440 cc        1 HM1     -0.184524
     23 C2C        15.5350    2.3950    3.2080 cc        1 HM1      0.264660
     24 C3C        16.8720    2.8740    3.1040 cd        1 HM1     -0.131182
     25 C4C        17.0300    2.7910    1.6970 cd        1 HM1      0.077844
     26 CMC        14.7130    2.1930    4.5240 c3        1 HM1     -0.359027
     27 CAC        17.8360    3.4290    4.0880 cf        1 HM1     -0.038956
     28 CBC        17.4290    3.9780    5.2710 c2        1 HM1     -0.473286
     29 C1D        18.5210    2.9330   -0.3530 cc        1 HM1     -0.105858
     30 C2D        19.6590    3.5220   -1.0320 cc        1 HM1      0.267954
     31 C3D        19.5990    3.1500   -2.3230 cd        1 HM1     -0.302159
     32 C4D        18.5100    2.2220   -2.4450 cd        1 HM1      0.168903
     33 CMD        20.5110    4.5600   -0.4120 c3        1 HM1     -0.428588
     34 CAD        20.4040    3.5600   -3.4770 c3        1 HM1      0.099838
     35 CBD        21.5470    2.6220   -3.8360 c3        1 HM1     -0.072299
     36 CGD        22.4280    3.1190   -5.0090 c         1 HM1      0.638236
     37 O1D        23.5810    2.7280   -5.1890 o         1 HM1     -0.694772
     38 O2D        21.9790    4.0100   -5.7140 o         1 HM1     -0.694772
     39 NA         15.9600    0.7600   -2.9110 Y2        1 HM1     -0.077950
     40 NB         14.0190    0.8080   -0.6540 Y3        1 HM1     -0.068461
     41 NC         15.9550    2.2380    1.0170 Y4        1 HM1     -0.182160
     42 ND         17.8100    2.1670   -1.2400 Y5        1 HM1     -0.129762
     43 HHA        18.7660    1.6880   -4.4150 ha        1 HM1      0.233380
     44 HHB        13.1640   -0.8560   -3.5030 ha        1 HM1      0.116538
     45 HHC        13.2300    1.3030    2.6420 ha        1 HM1      0.097950
     46 HHD        18.9960    3.5360    1.7230 ha        1 HM1      0.124708
     47 HAA2       16.9050   -0.9420   -6.8210 hc        1 HM1      0.035666
     48 HAA3       18.4120   -0.7260   -5.7870 hc        1 HM1      0.035666
     49 HBA2       16.8960    1.5910   -7.1570 hc        1 HM1      0.020524
     50 HBA3       18.6710    1.5360   -6.5760 hc        1 HM1      0.020524
     51 HAB        10.3950   -0.8640    1.2500 ha        1 HM1      0.101991
     52 HAC        18.9010    3.4540    3.8700 ha        1 HM1      0.114301
     53 HAD2       19.7410    3.6090   -4.3490 hc        1 HM1      0.001523
     54 HAD3       20.7870    4.5720   -3.2960 hc        1 HM1      0.001523
     55 HBD2       22.2440    2.5010   -2.9970 hc        1 HM1      0.000980
     56 HBD3       21.2350    1.5980   -4.0770 hc        1 HM1      0.000980
     57 HBB1       12.7380   -0.7300    3.2030 ha        1 HM1      0.160888
     58 HBB2       10.8960   -1.0180    3.4940 ha        1 HM1      0.160888
     59 HBC1       16.4140    3.9510    5.6600 ha        1 HM1      0.165276
     60 HBC2       18.1770    4.4290    5.9190 ha        1 HM1      0.165276
     61 HMB1       11.5910   -2.6020   -1.1670 hc        1 HM1      0.080052
     62 HMB2       11.4270   -1.6610   -2.5590 hc        1 HM1      0.080052
     63 HMB3       10.2250   -1.3800   -1.2340 hc        1 HM1      0.080052
     64 HMC1       14.0960    3.0690    4.7580 hc        1 HM1      0.094296
     65 HMC2       14.0120    1.3520    4.4660 hc        1 HM1      0.094296
     66 HMC3       15.2930    1.9640    5.4270 hc        1 HM1      0.094296
```

```
     67 HMA1       14.9020   -2.4930   -5.4050 hc         1 HM1       0.122215
     68 HMA2       14.9360   -1.3450   -6.6980 hc         1 HM1       0.122215
     69 HMA3       13.5060   -1.3240   -5.6840 hc         1 HM1       0.122215
     70 HMD1       20.7160    5.3080   -1.1870 hc         1 HM1       0.117257
     71 HMD2       21.4780    4.1120   -0.1530 hc         1 HM1       0.117257
     72 HMD3       20.0130    5.0280    0.4460 hc         1 HM1       0.117257
@<TRIPOS>BOND
     1    1    5 1
     2    1   32 1
     3    1   43 1
     4    2    8 1
     5    2   15 1
     6    2   44 1
     7    3   18 1
     8    3   22 1
     9    3   45 1
    10    4   25 1
    11    4   29 1
    12    4   46 1
    13    5    6 1
    14    5   39 1
    15    6    7 1
    16    6   10 1
    17    7    8 1
    18    7    9 1
    19    8   39 1
    20    9   67 1
    21    9   68 1
    22    9   69 1
    23   10   11 1
    24   10   47 1
    25   10   48 1
    26   11   12 1
    27   11   49 1
    28   11   50 1
    29   12   13 1
    30   12   14 1
    31   15   16 1
    32   15   40 1
    33   16   17 1
    34   16   19 1
    35   17   18 1
    36   17   20 1
    37   18   40 1
    38   19   61 1
    39   19   62 1
    40   19   63 1
    41   20   21 1
    42   20   51 1
    43   21   57 1
    44   21   58 1
    45   22   23 1
    46   22   41 1
    47   23   24 1
    48   23   26 1
    49   24   25 1
    50   24   27 1
    51   25   41 1
    52   26   64 1
    53   26   65 1
    54   26   66 1
    55   27   28 1
    56   27   52 1
    57   28   59 1
    58   28   60 1
    59   29   30 1
    60   29   42 1
    61   30   31 1
    62   30   33 1
    63   31   32 1
    64   31   34 1
    65   32   42 1
    66   33   70 1
    67   33   71 1
    68   33   72 1
    69   34   35 1
    70   34   53 1
    71   34   54 1
    72   35   36 1
```

```
    73   35   55 1
    74   35   56 1
    75   36   37 1
    76   36   38 1
@<TRIPOS>SUBSTRUCTURE
     1 HM1         1 TEMP              0 ****  ****    0 ROOT
```

FE1.mol2

```
#Fe(III) in low spin
@<TRIPOS>MOLECULE
FE1
    1     0     1     0     0
SMALL
RESP Charge


@<TRIPOS>ATOM
      1 FE         15.9180    1.6090   -1.0190 M1         1 FE1      0.702556
@<TRIPOS>BOND
@<TRIPOS>SUBSTRUCTURE
     1 FE1         1 TEMP              0 ****  ****    0 ROOT
```

TO1.mol2

```
@<TRIPOS>MOLECULE
TO1
   20    20     1     0     0
SMALL
RESP Charge


@<TRIPOS>ATOM
      1 N1         13.8690   -5.3410    5.5330 n          1 TO1     -0.415700
      2 C2         14.5740   -4.8610    4.3630 c3         1 TO1      0.001400
      3 H3         14.0860   -5.3230    3.5050 h1         1 TO1      0.071697
      4 C4         14.5190   -3.3750    4.2260 c3         1 TO1     -0.623221
      5 H5         13.4520   -3.2110    4.0790 hc         1 TO1      0.214006
      6 H6         14.7600   -2.8190    5.1320 hc         1 TO1      0.214006
      7 C7         15.2620   -2.7570    3.0030 ca         1 TO1      0.140823
      8 C8         16.2700   -1.8550    3.1390 ca         1 TO1     -0.161026
      9 H9         16.5210   -1.5060    4.1300 ha         1 TO1      0.122614
     10 C10        16.8100   -1.1880    2.0060 ca         1 TO1     -0.277841
     11 H11        17.6620   -0.5380    2.1480 ha         1 TO1      0.144588
     12 C12        16.1820   -1.3720    0.7610 ca         1 TO1      0.569026
     13 O13        16.5110   -0.6690   -0.4170 Y6         1 TO1     -0.636697
     14 C14        15.1010   -2.3410    0.6140 ca         1 TO1     -0.277841
     15 H15        14.6050   -2.5020   -0.3320 ha         1 TO1      0.144588
     16 C16        14.7120   -3.0090    1.7890 ca         1 TO1     -0.161026
     17 H17        13.9130   -3.7250    1.6670 ha         1 TO1      0.122614
     18 C18        15.9880   -5.3540    4.3970 c          1 TO1      0.597300
     19 O19        16.6650   -5.3930    5.4020 o          1 TO1     -0.567900
     20 H21        14.1830   -5.0760    6.4550 hn         1 TO1      0.278316
@<TRIPOS>BOND
     1    1    2 1
     2    1   20 1
     3    2    3 1
     4    2    4 1
     5    2   18 1
     6    4    5 1
     7    4    6 1
     8    4    7 1
     9    7    8 1
    10    7   16 1
    11    8    9 1
    12    8   10 1
    13   10   11 1
    14   10   12 1
    15   12   13 1
    16   12   14 1
    17   14   15 1
    18   14   16 1
    19   16   17 1
    20   18   19 1
@<TRIPOS>SUBSTRUCTURE
     1 TO1         1 TEMP              0 ****  ****    0 ROOT
```

HD1.mol2

```
@<TRIPOS>MOLECULE
HD1
   17    17     1     0     0
SMALL
RESP Charge


@<TRIPOS>ATOM
      1 N          12.8770    7.4400   -3.1180 N         1 HD1     -0.415700
      2 CA         13.0650    7.5440   -1.6780 CX        1 HD1      0.018800
      3 C          12.8160    8.9770   -1.1800 C         1 HD1      0.597300
      4 O          13.0920    9.9530   -1.8520 O         1 HD1     -0.567900
      5 CB         14.5620    7.0950   -1.3590 CT        1 HD1     -0.022164
      6 CG         14.8790    5.7090   -1.7820 CC        1 HD1     -0.033029
      7 ND1        15.4800    5.3550   -2.9540 NA        1 HD1     -0.140508
      8 CD2        14.7930    4.5220   -1.0640 CV        1 HD1     -0.118517
      9 CE1        15.7540    3.9900   -2.8900 CR        1 HD1     -0.040546
     10 NE2        15.2850    3.4100   -1.7940 Y1        1 HD1     -0.114100
     11 H          13.5140    8.0670   -3.5890 H         1 HD1      0.287320
     12 HA         12.3960    6.8330   -1.1940 H1        1 HD1      0.085699
     13 HB2        15.1720    7.7890   -1.9390 HC        1 HD1      0.029159
     14 HB3        14.8240    7.2090   -0.3080 HC        1 HD1      0.029159
     15 HD2        14.3700    4.3530   -0.0850 H4        1 HD1      0.143576
     16 HE1        16.1980    3.4250   -3.6960 H5        1 HD1      0.157610
     17 HD1        15.6530    5.9780   -3.7300 H         1 HD1      0.289802
@<TRIPOS>BOND
     1    1    2 1
     2    1   11 1
     3    2    3 1
     4    2    5 1
     5    2   12 1
     6    3    4 1
     7    5    6 1
     8    5   13 1
     9    5   14 1
    10    6    7 1
    11    6    8 1
    12    7    9 1
    13    7   17 1
    14    8   10 1
    15    8   15 1
    16    9   10 1
    17    9   16 1
@<TRIPOS>SUBSTRUCTURE
     1 HD1         1 TEMP              0 ****  ****    0 ROOT
```

MCPB.frcmod

```
#For heme-Fe(III) in low spin, generated by MCPB.py
MASS
M1 55.85                              Fe ion
Y1 14.01         0.530               sp2 N in 5 memb.ring w/LP (HIS,ADE,GUA)
Y2 14.01         0.530               Sp2 N in non-pure aromatic systems, identical to nc
Y3 14.01         0.530               Sp2 N in non-pure aromatic systems, identical to nc
Y4 14.01         0.530               Sp2 N in non-pure aromatic systems, identical to nc
Y5 14.01         0.530               Sp2 N in non-pure aromatic systems, identical to nc
Y6 16.00         0.434                Oxygen with one connected atom

BOND
Y1-M1   46.8    2.0628      Created by Seminario method using MCPB.py
Y2-M1  103.3    2.0265      Created by Seminario method using MCPB.py
Y3-M1   98.1    2.0264      Created by Seminario method using MCPB.py
Y4-M1  104.8    2.0255      Created by Seminario method using MCPB.py
Y5-M1   96.0    2.0246      Created by Seminario method using MCPB.py
Y6-M1   92.9    1.8853      Created by Seminario method using MCPB.py
CR-Y1  488.0    1.335       JCC,7,(1986),230; HIS
CV-Y1  410.0    1.394       JCC,7,(1986),230; HIS
ca-Y6  598.1    1.2358      SOURCE4_SOURCE5      17  0.0088
cc-Y2  525.4    1.3172      SOURCE3_SOURCE5    4612  0.0083
cc-Y3  525.4    1.3172      SOURCE3_SOURCE5    4612  0.0083
cc-Y4  525.4    1.3172      SOURCE3_SOURCE5    4612  0.0083
cc-Y5  525.4    1.3172      SOURCE3_SOURCE5    4612  0.0083
cd-Y2  441.1    1.3694      SOURCE1_SOURCE5    2269  0.0086
cd-Y3  441.1    1.3694      SOURCE1_SOURCE5    2269  0.0086
cd-Y4  441.1    1.3694      SOURCE1_SOURCE5    2269  0.0086
cd-Y5  441.1    1.3694      SOURCE1_SOURCE5    2269  0.0086
```

```
ANGL
CR-Y1-M1   119.49      126.08    Created by Seminario method using MCPB.py
CV-Y1-M1   126.42      127.54    Created by Seminario method using MCPB.py
Y1-M1-Y2    91.10       88.51    Created by Seminario method using MCPB.py
Y1-M1-Y3    96.05       88.83    Created by Seminario method using MCPB.py
Y1-M1-Y4    93.89       88.88    Created by Seminario method using MCPB.py
Y1-M1-Y5    89.13       89.20    Created by Seminario method using MCPB.py
Y1-M1-Y6   127.79      176.26    Created by Seminario method using MCPB.py
Y2-M1-Y3    81.08       91.28    Created by Seminario method using MCPB.py
Y2-M1-Y4    71.60      177.37    Created by Seminario method using MCPB.py
Y2-M1-Y5    85.03       88.61    Created by Seminario method using MCPB.py
Y2-M1-Y6    89.59       88.23    Created by Seminario method using MCPB.py
Y3-M1-Y4    66.45       88.94    Created by Seminario method using MCPB.py
Y3-M1-Y5    75.26      178.02    Created by Seminario method using MCPB.py
Y3-M1-Y6    85.16       93.07    Created by Seminario method using MCPB.py
Y4-M1-Y5    68.71       91.08    Created by Seminario method using MCPB.py
Y4-M1-Y6    89.30       94.37    Created by Seminario method using MCPB.py
Y5-M1-Y6    84.90       88.90    Created by Seminario method using MCPB.py
ca-Y6-M1   109.88      129.73    Created by Seminario method using MCPB.py
cc-Y2-M1   191.96      128.06    Created by Seminario method using MCPB.py
cc-Y3-M1   187.77      125.48    Created by Seminario method using MCPB.py
cc-Y4-M1   192.30      127.63    Created by Seminario method using MCPB.py
cc-Y5-M1   187.36      125.75    Created by Seminario method using MCPB.py
cd-Y2-M1   191.24      125.89    Created by Seminario method using MCPB.py
cd-Y3-M1   190.78      127.99    Created by Seminario method using MCPB.py
cd-Y4-M1   193.65      126.05    Created by Seminario method using MCPB.py
cd-Y5-M1   189.74      128.09    Created by Seminario method using MCPB.py
CC-CV-Y1    70.0       120.00    AA his
CV-Y1-CR    70.0       117.00    AA his
H4-CV-Y1    50.0       120.00    AA his
H5-CR-Y1    50.0       120.00    AA his
NA-CR-Y1    70.0       120.00    AA his
Y2-cc-cc    71.6       112.56             SOURCE3          141    4.2871
Y3-cc-cc    71.6       112.56             SOURCE3          141    4.2871
Y4-cc-cc    71.6       112.56             SOURCE3          141    4.2871
Y5-cc-cc    71.6       112.56             SOURCE3          141    4.2871
ca-ca-Y6    71.4       123.26      SOURCE4_SOURCE5          35    1.2620
cc-Y2-cd    71.8       105.49         CORR_SOURCE5        1810    1.9032
cc-Y3-cd    71.8       105.49         CORR_SOURCE5        1810    1.9032
cc-Y4-cd    71.8       105.49         CORR_SOURCE5        1810    1.9032
cc-Y5-cd    71.8       105.49         CORR_SOURCE5        1810    1.9032
cd-cd-Y2    67.6       121.98         CORR_SOURCE5         141    1.9633
cd-cd-Y3    67.6       121.98         CORR_SOURCE5         141    1.9633
cd-cd-Y4    67.6       121.98         CORR_SOURCE5         141    1.9633
cd-cd-Y5    67.6       121.98         CORR_SOURCE5         141    1.9633
ce-cc-Y2    68.1       121.70         CORR_SOURCE5          58    1.4179
ce-cc-Y3    68.1       121.70         CORR_SOURCE5          58    1.4179
ce-cc-Y4    68.1       121.70         CORR_SOURCE5          58    1.4179
ce-cc-Y5    68.1       121.70         CORR_SOURCE5          58    1.4179
ce-cd-Y2    68.7       123.98      SOURCE4_SOURCE5          10    2.4097
ce-cd-Y3    68.7       123.98      SOURCE4_SOURCE5          10    2.4097
ce-cd-Y4    68.7       123.98      SOURCE4_SOURCE5          10    2.4097
ce-cd-Y5    68.7       123.98      SOURCE4_SOURCE5          10    2.4097

DIHE
X -CR-Y1-X     2       10.0      180.0   2.0         JCC,7,(1986),230
X -CV-Y1-X     2        4.8      180.0   2.0         JCC,7,(1986),230
X -cc-Y2-X     2        9.5      180.0   2.0      statistic value from parm94
X -cc-Y3-X     2        9.5      180.0   2.0      statistic value from parm94
X -cc-Y4-X     2        9.5      180.0   2.0      statistic value from parm94
X -cc-Y5-X     2        9.5      180.0   2.0      statistic value from parm94
X -cd-Y2-X     2        9.5      180.0   2.0      statistiv value from parm94
X -cd-Y3-X     2        9.5      180.0   2.0      statistiv value from parm94
X -cd-Y4-X     2        9.5      180.0   2.0      statistiv value from parm94
X -cd-Y5-X     2        9.5      180.0   2.0      statistiv value from parm94
CC-CV-Y1-M1    3       0.00       0.00   3.0    Treat as zero by MCPB.py
CR-Y1-M1-Y2    3       0.00       0.00   3.0    Treat as zero by MCPB.py
CR-Y1-M1-Y3    3       0.00       0.00   3.0    Treat as zero by MCPB.py
CR-Y1-M1-Y4    3       0.00       0.00   3.0    Treat as zero by MCPB.py
CR-Y1-M1-Y5    3       0.00       0.00   3.0    Treat as zero by MCPB.py
CR-Y1-M1-Y6    3       0.00       0.00   3.0    Treat as zero by MCPB.py
CV-Y1-M1-Y2    3       0.00       0.00   3.0    Treat as zero by MCPB.py
CV-Y1-M1-Y3    3       0.00       0.00   3.0    Treat as zero by MCPB.py
CV-Y1-M1-Y4    3       0.00       0.00   3.0    Treat as zero by MCPB.py
CV-Y1-M1-Y5    3       0.00       0.00   3.0    Treat as zero by MCPB.py
CV-Y1-M1-Y6    3       0.00       0.00   3.0    Treat as zero by MCPB.py
H4-CV-Y1-M1    3       0.00       0.00   3.0    Treat as zero by MCPB.py
H5-CR-Y1-M1    3       0.00       0.00   3.0    Treat as zero by MCPB.py
M1-Y2-cc-cc    3       0.00       0.00   3.0    Treat as zero by MCPB.py
```

```
M1-Y3-cc-cc    3       0.00       0.00   3.0    Treat as zero by MCPB.py
M1-Y4-cc-cc    3       0.00       0.00   3.0    Treat as zero by MCPB.py
M1-Y5-cc-cc    3       0.00       0.00   3.0    Treat as zero by MCPB.py
NA-CR-Y1-M1    3       0.00       0.00   3.0    Treat as zero by MCPB.py
Y1-M1-Y2-cc    3       0.00       0.00   3.0    Treat as zero by MCPB.py
Y1-M1-Y2-cd    3       0.00       0.00   3.0    Treat as zero by MCPB.py
Y1-M1-Y3-cc    3       0.00       0.00   3.0    Treat as zero by MCPB.py
Y1-M1-Y3-cd    3       0.00       0.00   3.0    Treat as zero by MCPB.py
Y1-M1-Y4-cc    3       0.00       0.00   3.0    Treat as zero by MCPB.py
Y1-M1-Y4-cd    3       0.00       0.00   3.0    Treat as zero by MCPB.py
Y1-M1-Y5-cc    3       0.00       0.00   3.0    Treat as zero by MCPB.py
Y1-M1-Y5-cd    3       0.00       0.00   3.0    Treat as zero by MCPB.py
Y1-M1-Y6-ca    3       0.00       0.00   3.0    Treat as zero by MCPB.py
Y2-M1-Y6-ca    3       0.00       0.00   3.0    Treat as zero by MCPB.py
Y3-M1-Y6-ca    3       0.00       0.00   3.0    Treat as zero by MCPB.py
Y3-cc-ce-cd    4        4.0      180.0   2.0      same as X -ce-ce-X , penalty
score=136.0
Y4-M1-Y6-ca    3       0.00       0.00   3.0    Treat as zero by MCPB.py
Y4-cc-ce-cd    4        4.0      180.0   2.0      same as X -ce-ce-X , penalty
score=136.0
Y5-M1-Y6-ca    3       0.00       0.00   3.0    Treat as zero by MCPB.py
Y5-cc-ce-cd    4        4.0      180.0   2.0      same as X -ce-ce-X , penalty
score=136.0
Y5-cd-ce-cc    4       26.6      180.0   2.0      same as X -ce-cf-X , penalty
score=136.0
ca-ca-Y6-M1    3       0.00       0.00   3.0    Treat as zero by MCPB.py
cc-Y2-M1-Y3    3       0.00       0.00   3.0    Treat as zero by MCPB.py
cc-Y2-M1-Y4    3       0.00       0.00   3.0    Treat as zero by MCPB.py
cc-Y2-M1-Y5    3       0.00       0.00   3.0    Treat as zero by MCPB.py
cc-Y2-M1-Y6    3       0.00       0.00   3.0    Treat as zero by MCPB.py
cc-Y3-M1-Y2    3       0.00       0.00   3.0    Treat as zero by MCPB.py
cc-Y3-M1-Y4    3       0.00       0.00   3.0    Treat as zero by MCPB.py
cc-Y3-M1-Y5    3       0.00       0.00   3.0    Treat as zero by MCPB.py
cc-Y3-M1-Y6    3       0.00       0.00   3.0    Treat as zero by MCPB.py
cc-Y4-M1-Y2    3       0.00       0.00   3.0    Treat as zero by MCPB.py
cc-Y4-M1-Y3    3       0.00       0.00   3.0    Treat as zero by MCPB.py
cc-Y4-M1-Y5    3       0.00       0.00   3.0    Treat as zero by MCPB.py
cc-Y4-M1-Y6    3       0.00       0.00   3.0    Treat as zero by MCPB.py
cc-Y5-M1-Y2    3       0.00       0.00   3.0    Treat as zero by MCPB.py
cc-Y5-M1-Y3    3       0.00       0.00   3.0    Treat as zero by MCPB.py
cc-Y5-M1-Y4    3       0.00       0.00   3.0    Treat as zero by MCPB.py
cc-Y5-M1-Y6    3       0.00       0.00   3.0    Treat as zero by MCPB.py
cc-ce-cd-Y2    4       26.6      180.0   2.0      same as X -ce-cf-X , penalty
score=136.0
cc-ce-cd-Y3    4       26.6      180.0   2.0      same as X -ce-cf-X , penalty
score=136.0
cc-ce-cd-Y4    4       26.6      180.0   2.0      same as X -ce-cf-X , penalty
score=136.0
cd-Y2-M1-Y3    3       0.00       0.00   3.0    Treat as zero by MCPB.py
cd-Y2-M1-Y4    3       0.00       0.00   3.0    Treat as zero by MCPB.py
cd-Y2-M1-Y5    3       0.00       0.00   3.0    Treat as zero by MCPB.py
cd-Y2-M1-Y6    3       0.00       0.00   3.0    Treat as zero by MCPB.py
cd-Y3-M1-Y2    3       0.00       0.00   3.0    Treat as zero by MCPB.py
cd-Y3-M1-Y4    3       0.00       0.00   3.0    Treat as zero by MCPB.py
cd-Y3-M1-Y5    3       0.00       0.00   3.0    Treat as zero by MCPB.py
cd-Y3-M1-Y6    3       0.00       0.00   3.0    Treat as zero by MCPB.py
cd-Y4-M1-Y2    3       0.00       0.00   3.0    Treat as zero by MCPB.py
cd-Y4-M1-Y3    3       0.00       0.00   3.0    Treat as zero by MCPB.py
cd-Y4-M1-Y5    3       0.00       0.00   3.0    Treat as zero by MCPB.py
cd-Y4-M1-Y6    3       0.00       0.00   3.0    Treat as zero by MCPB.py
cd-Y5-M1-Y2    3       0.00       0.00   3.0    Treat as zero by MCPB.py
cd-Y5-M1-Y3    3       0.00       0.00   3.0    Treat as zero by MCPB.py
cd-Y5-M1-Y4    3       0.00       0.00   3.0    Treat as zero by MCPB.py
cd-Y5-M1-Y6    3       0.00       0.00   3.0    Treat as zero by MCPB.py
cd-cd-Y2-M1    3       0.00       0.00   3.0    Treat as zero by MCPB.py
cd-cd-Y3-M1    3       0.00       0.00   3.0    Treat as zero by MCPB.py
cd-cd-Y4-M1    3       0.00       0.00   3.0    Treat as zero by MCPB.py
cd-cd-Y5-M1    3       0.00       0.00   3.0    Treat as zero by MCPB.py
cd-ce-cc-Y2    4        4.0      180.0   2.0      same as X -ce-ce-X , penalty
score=136.0
ce-cc-Y2-M1    3       0.00       0.00   3.0    Treat as zero by MCPB.py
ce-cc-Y3-M1    3       0.00       0.00   3.0    Treat as zero by MCPB.py
ce-cc-Y4-M1    3       0.00       0.00   3.0    Treat as zero by MCPB.py
ce-cc-Y5-M1    3       0.00       0.00   3.0    Treat as zero by MCPB.py
ce-cd-Y2-M1    3       0.00       0.00   3.0    Treat as zero by MCPB.py
ce-cd-Y3-M1    3       0.00       0.00   3.0    Treat as zero by MCPB.py
ce-cd-Y4-M1    3       0.00       0.00   3.0    Treat as zero by MCPB.py
ce-cd-Y5-M1    3       0.00       0.00   3.0    Treat as zero by MCPB.py
```

```
ha-ce-cc-Y2    4        4.0      180.0   2.0      same as X -ce-ce-X , penalty
score=136.0
ha-ce-cc-Y3    4        4.0      180.0   2.0      same as X -ce-ce-X , penalty
score=136.0
ha-ce-cc-Y4    4        4.0      180.0   2.0      same as X -ce-ce-X , penalty
score=136.0
ha-ce-cc-Y5    4        4.0      180.0   2.0      same as X -ce-ce-X , penalty
score=136.0
ha-ce-cd-Y2    4       16.0      180.0   2.0      same as X -cc-cd-X , penalty
score=136.0
ha-ce-cd-Y3    4       16.0      180.0   2.0      same as X -cc-cd-X , penalty
score=136.0
ha-ce-cd-Y4    4       16.0      180.0   2.0      same as X -cc-cd-X , penalty
score=136.0
ha-ce-cd-Y5    4       16.0      180.0   2.0      same as X -cc-cd-X , penalty
score=136.0

IMPR
Y2-cc-cc-ce         1.1          180.0         2.0          Using the default value
Y5-cd-cd-ce         1.1          180.0         2.0          Using the default value
Y2-cd-cd-ce         1.1          180.0         2.0          Using the default value
Y3-cc-cc-ce         1.1          180.0         2.0          Using the default value
Y3-cd-cd-ce         1.1          180.0         2.0          Using the default value
Y4-cc-cc-ce         1.1          180.0         2.0          Using the default value
Y4-cd-cd-ce         1.1          180.0         2.0          Using the default value
Y5-cc-cc-ce         1.1          180.0         2.0          Using the default value
Y6-ca-ca-ca         1.1          180.0         2.0          Using the default value

NONB
  M1          1.3910  0.0143067400       IOD set for Fe2+ ion for the OPC water model
from Li et al. JCTC, 2020, 16, 4429
  Y1          1.8240  0.1700             OPLS
  Y2          1.8240  0.1700             OPLS
  Y3          1.8240  0.1700             OPLS
  Y4          1.8240  0.1700             OPLS
  Y5          1.8240  0.1700             OPLS
  Y6          1.6612  0.2100             OPLS
```

HM1.frcmod

```
#For heme
MASS

BOND

ANGLE

DIHE
cd-cd-ce-cc   4   26.600       180.000           2.000      same as X -ce-cf-X , penalty
score=136.0
nd-cd-ce-cc   4   26.600       180.000           2.000      same as X -ce-cf-X , penalty
score=136.0
cc-cc-ce-cd   4    4.000       180.000           2.000      same as X -ce-ce-X , penalty
score=136.0
cc-cc-ce-ha   4    4.000       180.000           2.000      same as X -ce-ce-X , penalty
score=136.0
cd-cd-ce-ha   4   16.000       180.000           2.000      same as X -cc-cd-X , penalty
score=136.0
nd-cc-ce-cd   4    4.000       180.000           2.000      same as X -ce-ce-X , penalty
score=136.0
cc-cd-cf-c2   4    4.000       180.000           2.000      same as X -cf-cf-X , penalty
score=136.0
cc-cd-cf-ha   4    4.000       180.000           2.000      same as X -cf-cf-X , penalty
score=136.0
cd-cd-cf-c2   4    4.000       180.000           2.000      same as X -cf-cf-X , penalty
score=136.0
cd-cd-cf-ha   4    4.000       180.000           2.000      same as X -cf-cf-X , penalty
score=136.0
nd-cc-ce-ha   4    4.000       180.000           2.000      same as X -ce-ce-X , penalty
score=136.0
nd-cd-ce-ha   4   16.000       180.000           2.000      same as X -cc-cd-X , penalty
score=136.0

IMPROPER
cc-cd-ce-ha         1.1          180.0         2.0          Same as X -X -ca-ha, penalty
score= 46.8 (use general term))
cc-ce-cc-nd         1.1          180.0         2.0          Using the default value
c3-cc-cc-cd         1.1          180.0         2.0          Using the default value
```

```
c3-cc-cd-cd         1.1          180.0         2.0          Using the default value
cd-ce-cd-nd         1.1          180.0         2.0          Using the default value
c3-o -c -o          1.1          180.0         2.0          Using general improper
torsional angle  X- o- c- o, penalty score=  3.0)
cc-cd-cd-cf         1.1          180.0         2.0          Same as c2-ca-ca-ca, penalty
score=304.0)
c2-cd-cf-ha         1.1          180.0         2.0          Same as X -X -ca-ha, penalty
score= 46.8 (use general term))
cf-ha-c2-ha         1.1          180.0         2.0          Same as X -X -ca-ha, penalty
score= 47.1 (use general term))

NONBON
```

TO1.frcmod

```
#For TYR
MASS

BOND
c -N   427.6    1.3789
C -n   427.6    1.3789

ANGLE
n -c -o     74.2      123.05
C -n -c3    63.4      120.69
n -C -O     74.2      123.05
N -c -o     74.2      123.05
C -n -hn    48.3      117.55
CX-C -n     67.5      114.57
c -N -CX    63.7      122.15
C -N -H     50.0      119.20
c -N -H     50.0      119.20
c3-c -N     66.8      115.18

DIHE
X -C -n -X    4   10.000       180.000           2.000      AA,NMA (no c-n3, c-n4, c-nh)
X -c -N -X    4   10.000       180.000           2.000      AA,NMA (no c-n3, c-n4, c-nh)

IMPROPER
ca-ca-ca-ha         1.1          180.0         2.0          Using general improper
torsional angle  X- X-ca-ha, penalty score=  6.0)
ca-ca-ca-o          1.1          180.0         2.0          Using the default value
c3-n -c -o         10.5          180.0         2.0          Using general improper
torsional angle  X- X- c- o, penalty score=  6.0)
c -hn-n -hn         1.1          180.0         2.0          Using general improper
torsional angle  X- X- n-hn, penalty score=  6.0)
c3-N -C -o         10.5          180.0         2.0          Using general improper
torsional angle  X- X- c- o, penalty score=  6.0)
c -hn-N -hn         1.1          180.0         2.0          Using general improper
torsional angle  X- X- n-hn, penalty score=  6.0)

NONBON
```
